# Selumetinib in Combination with Anti Retroviral Therapy in HIV-associated Kaposi sarcoma (SCART): an open-label, multicentre, phase I/II trial

**DOI:** 10.1186/s12885-025-13890-x

**Published:** 2025-03-19

**Authors:** Robin J. Young, Amanda Kirkham, Joshua Savage, Charlotte Gaskell, Sarah Johnson, David H. Dockrell, Mark Bower, Sarah Westwell, Christine Bowman, Michael Leahy, Penella Woll, Lucinda Billingham

**Affiliations:** 1 University of Sheffield, Sheffield, UK; 2 Sheffield Teaching Hospitals NHS Foundation Trust, Sheffield, UK; 3 Cancer Research UK Clinical Trials Unit (CRCTU), University of Birmingham, Birmingham, UK; 4 Institute for Regeneration and Repair, Centre for Inflammation Research, University of Edinburgh, Edinburgh, UK; 5 National Centre for HIV Malignancy, Chelsea & Westminster Hospital, London, UK; 6 Brighton and Sussex University Hospitals NHS Trust, Brighton, UK; 7 The Christie NHS Foundation Trust, Manchester, UK

**Keywords:** Kaposi sarcoma, HIV, AIDS, MEK inhibitor, Selumetinib, Early phase clinical trial, ART

## Abstract

**Background:**

Kaposi sarcoma (KS) is the commonest HIV-associated malignancy. It is caused by co-infection with Kaposi sarcoma herpesvirus (KSHV), which upregulates the MAPK pathway. The aim of the SCART trial was to identify a safe dose for the MEK inhibitor selumetinib in combination with antiretroviral therapy (ART) and to establish evidence of the combination’s efficacy.

**Methods:**

SCART was a prospective, single arm, open-label, multi-centre, phase I/II trial, recruiting from four UK centres. Eligible patients were HIV positive, established on an ART regimen ≥ 3 months, had HIV viral load ≤ 200/ml, and had histologically confirmed KS with progressive disease. Phase I primary outcomes were occurrence of dose limiting toxicity (DLT) to determine the maximum tolerated dose/recommended phase II dose (RP2D), and pharmacokinetic assessments of selumetinib and N-desmethyl metabolite. Phase II primary outcome was occurrence of objective response (OR) as defined by AIDS Clinical Trials Group (ACTG) criteria.

**Results:**

Between 15-Jun-2012 and 25-Sep-2018, 19 patients were recruited; three did not start treatment and were not included in the final analysis. Ten eligible patients were treated in phase I and an additional six in phase II. There was one DLT at the 75 mg bd dose, which was deemed to be the RP2D. Of those patients receiving the RP2D (six within phase I, six within phase II), one achieved a partial response (OR 8.3%, 90% confidence interval: 0.4, 33.9). Further to the DLT, two serious adverse reactions, one unrelated serious adverse event (AE), and six non-serious grade 3 AEs were reported, together with 360 AEs graded 1 or 2. No detrimental impact on ART drug levels or HIV viral load were observed, with improvements in CD4 count and evidence of response in Angiopoietin-2 demonstrated.

**Conclusions:**

SCART was closed early due to slow recruitment, partly due to the rarity of KS because of improvements in HIV care, but also due to patients’ concerns about experiencing non-serious toxicity additional to those from ART. Although we cannot recommend the use of 75 mg bd selumetinib with ART in patients with HIV-associated KS, studies exploring selumetinib in combination with other agents including anti-angiogenic agents and/or immune checkpoint inhibitors are warranted.

**Trial registration:**

ISRCTN24921472.

**Supplementary Information:**

The online version contains supplementary material available at 10.1186/s12885-025-13890-x.

## Background

Since the introduction of antiretroviral therapy (ART), the risk of acquired immunodeficiency syndrome (AIDS)-defining cancers has reduced. However, Kaposi sarcoma (KS) still accounts for a substantial burden of malignancy in people living with HIV [1]. KS typically presents as multi-focal violaceous cutaneous lesions, often with associated lymphoedema. In HIV-associated KS, extra-cutaneous disease sites are not infrequent, including lymph nodes, gastrointestinal tract, and lungs.

The first-line treatment of HIV-associated KS is ART, and for early stage KS, this is usually sufficient to control the disease [2]. However, in patients with advanced KS, and particularly in those with visceral disease, additional treatment with systemic chemotherapy is indicated, usually with either liposomal danthracycline or paclitaxel [3]. Despite initial responses to chemotherapy, most patients eventually progress or relapse, and new treatment alternatives are required. Anti-angiogenic and immune-modulatory approaches have previously been studied including bevacizumab [4] and pomalidomide [5], and more recently immune checkpoint inhibitors [6].

In all forms of KS, the malignant spindle cells harbour Kaposi sarcoma herpesvirus (KSHV), also known as human herpesvirus-8 (HHV8). This virus is the aetiological agent responsible for KS. The MAPK pathway protein, mitogen-activated protein kinase kinase (MEK), is important in viral infections including KSHV/HHV8 and HIV during the early infection phase, and in reactivation following viral latency [7–11]. In KS, the KSHV/HHV8 encoded proteins viral interleukin-6 (vIL-6) and viral G protein-coupled receptor (vGPCR) upregulate the Ras/Raf/MEK/ERK pathway [10]. Furthermore, in KS this has been shown to stimulate expression of Angiopoietin (Ang)-2, a pro-angiogenic factor [10], which may further promote KS development, and plasma Ang-2 levels have been shown to correlate with disease burden [11]. The MAPK pathway therefore represents a potential therapeutic target in KS.

Selumetinib (AZD6244 Hyd-Sulphate; 6-(4-Bromo-2-chloro-phenylamino)-7-fluro-3-methyl-3 H-benzoimidazole-5-carboxylic acid (2-hydroxy-ethoxy)-amide hydrogen sulphate; ARRY-142886) is a selective inhibitor of MEK1/2, inhibiting the phosphorylation of ERK1/2 [12]. Its safety profile, dose, and efficacy have been established in phase I and II trials, both as monotherapy and combined with cytotoxic chemotherapy in patients with advanced solid malignancies including melanoma and non-small cell lung cancer [13–17]. Patients with significant co-morbidities such as HIV are routinely excluded from clinical trials of anti-cancer therapies and there have been few detailed studies of the interaction between oral targeted anti-cancer agents and ART. Given the potential for anti-cancer therapies to have an adverse impact on HIV control, a key consideration in our patient population, we decided to carefully test the combination of selumetinib with ART in HIV-associated KS using a phase I/II trial design.

The aims of the trial of selumetinib in combination with anti-retroviral therapy in HIV-associated Kaposi sarcoma (SCART) were to identify a safe dose and establish evidence of efficacy for selumetinib in combination with ART in patients with HIV-associated KS. The trial completed phase I but then closed early during phase II due to slow recruitment following the recommendation of the independent Data Monitoring Committee (DMC). This paper reports the final results from the trial.

## Methods

### Study design

The SCART trial was a single-arm, open label phase I/II clinical trial that recruited patients from four of six open hospitals in the UK. The current version of the protocol (version 12.0, 03-Feb-2017) is attached in Supplementary Appendix 1. The trial was prospectively registered: EudraCT Number: 2011-003099-35; ISRCTN24921472; ClinicalTrials.Gov: NCT01752569.

### Patients

Eligible patients were *≥* 18 years, WHO performance status ≤ 2 with adequate haematological, hepatic and renal function, HIV positive, established on a ART regimen for ≥ 3 months (the ART regimen was not pre-specified, details are available in Supplementary Appendix 2), had measurable disease according to AIDS Clinical Trials Group (ACTG) criteria (Supplementary Appendix 3), and progressive cutaneous or nodal KS not requiring chemotherapy or progressive KS following cytotoxic chemotherapy. Patients with poorly controlled HIV, defined as a viral load > 200viral copies/ml were excluded, as were patients with active opportunistic infections or those with active hepatitis B or C. Further eligibility criteria can be found in the protocol (Supplementary Appendix 1). All patients gave written informed consent.

Patient registration into the trial by the treating clinician was by telephone to the central registration service at the Cancer Research UK Clinical Trials Unit (CRCTU) at the University of Birmingham.

### Interventions

In the phase I component of the trial, patients were recruited to establish the dosage, safety, and pharmacokinetics of selumetinib in combination with ART. The trial used a standard 3 + 3 rule-based dose escalation algorithm with four pre-defined dose levels. Doses of selumetinib started at 50 mg with subsequent permitted dose levels of 75 mg and 100 mg, all taken orally twice daily (bd) in combination with ART, for 6 cycles of 21 days unless disease progression, unacceptable toxicity, or withdrawal of consent for any reason occurred. Patients recruited to 50 mg bd who experienced no toxicity in cycle 1 could be dose escalated to 75 mg bd selumetinib in subsequent cycles. A dose of 75 mg once daily (od) provided a dose level below the starting dose should de-escalation be required. A Safety Review Committee (SRC) of the study investigators and the trial team at the CRCTU met regularly to evaluate the data after every cohort of three patients had completed their first cycle of selumetinib, to evaluate the trial data including safety and dose limiting toxicities (DLTs), and to advise on dose escalation decisions as laid out in the protocol (Supplementary Appendix 1).

The aim of the phase I component of the trial was to determine the maximum tolerated dose (MTD) of selumetinib in combination with ART, which was defined as the highest dose producing one or fewer DLT in six patients. This dose was defined as the recommended phase II dose (RP2D) for the subsequent phase II component of the trial. Patients recruited to phase I who received the RP2D as their initial dose were included in the phase II population for response analysis. Having completed six cycles of treatment, patients who responded to treatment were permitted to continue selumetinib with ART at the discretion of the treating physician. Patients concurrently received best supportive therapy according to institutional guidelines.

### Clinical assessments

For registered patients, pre-treatment evaluation included: physical examination, ophthalmologic exam, electrocardiogram (ECG), echocardiogram or multi-gated acquisition (MUGA) scan, chest x-ray and CT thorax, abdomen, and pelvis. Adverse events according to CTCAE version 4.0 [18] were recorded every three weeks. The burden of cutaneous disease was estimated by summed tumour area sizes (mm^2^) from photographic image measurements and evaluated at baseline using ACTG criteria (Supplementary Appendix 3), with subsequent formal response assessments including clinical photographs taken every 6 weeks for 12 months or until disease progression. For patients with visceral or extensive disease, repeat CT scans at these time points were also performed.

### Sample collection and processing

Details of the schedule and methods for the pharmacokinetic analyses of plasma selumetinib and N-desmethyl metabolite, as well as therapeutic drug monitoring of the ART, and the pharmacodynamic analyses, are detailed in Supplementary Appendix 4.

### Outcome Measures

The primary outcome measures for phase I were the occurrence of DLTs and pharmacokinetic assessments of selumetinib and metabolite (N-desmethyl) plasma levels measured on days 1 (pre-dose, 2 and 6 h) and 15 (pre-dose only), and ART trough levels on days 1 and 15, and at 12 weeks. The occurrence of DLTs was used to evaluate the safety of each dose level and thereby determine the MTD which defined the RP2D. DLTs considered at least possibly related to selumetinib were assessed during cycle 1 using CTCAE version 4.0 [18] and defined as:

- Haematological.
- Absolute neutrophil count (ANC) < 1.0 × 10^9^/L.
- Febrile neutropenia (ANC < 1.0 × 10^9^/L, fever *≥* 38^o^C).
- Platelets < 50 × 10^9^/L.
- Bleeding thought to be due to thrombocytopenia.
- Non-haematological.
  - Diarrhoea *≥* grade 3 despite optimal loperamide use.
  - Rash *≥* grade 3.
  - Any grade 2 toxicity unacceptable to the patient that required a dose reduction.
  - Missing > 30% of treatment doses for toxicity reasons.
  - Any other grade 3 or 4 effects thought to be treatment related.
- HIV control.
  - Clinically necessary change in ART drug levels due to detrimental alteration in HIV viral load and/or CD4 cell count.

The primary outcome measure for phase II was the occurrence of an objective response (OR), defined as either a complete response (CR) or partial response (PR), assessed using ACTG criteria (Supplementary Appendix 3) following up to six cycles of treatment; this was also a secondary outcome for phase I. In addition, duration of response (DoR) was measured, defined as the time when measurement criteria for CR/PR were first met until the date that recurrent or progressive disease was objectively documented. Secondary outcomes for both phases were: incidence of adverse events assessed by CTCAE v 4.0; the number of selumetinib cycles completed; progression-free survival time (PFS) defined as the time from commencing treatment to either date of progression (defined as the first day when the ACTG criteria for progressive disease were met: Supplementary Appendix 3) or death from any cause, with patients who had not progressed or died at the time of analysis censored at their date last seen; HIV control measured by HIV-1 viral load pre-treatment and day 1 of cycles 2, 3, 5 and end of cycle 6, and CD4 cell counts pre-treatment and day 1 of cycle 4 and end of cycle 6. Secondary outcomes also included pharmacodynamic measures of selumetinib in combination with ART as assessed by serum concentration of angiogenesis markers, including serum Ang-1 and -2, and Tie-2, using enzyme-linked immunosorbent assays (ELISAs) (see Supplementary Appendix 4 for more details).

### Statistical analysis

The analysis included three different evaluable populations:

- Per-protocol population – defined as the response evaluable phase II patients (including those transferred over from phase I) who started selumetinib at the RP2D and received at least one cycle.
- MTD population – defined as patients from both phase I and phase II that received selumetinib at the MTD for at least one cycle.
- Any-dose/safety population – defined as the patients from both phase I and II that received at least one dose of selumetinib at any dose.

Adverse event data for phase I was reported descriptively using the safety population. In particular, the observed adverse events that constituted a DLT at each dose level have been reported to demonstrate the decisions made as part of the 3 + 3 dose-finding design described earlier. Pharmacokinetic data on selumetinib and metabolite plasma levels (specifically C_max_, AUC_0-24_, clearance, and t_1/2_) and ART drug levels (specifically percentage changes in antiretroviral drug levels) were described in this population.

The phase II component of the trial was designed as a Simon’s two stage minimax design, testing a null hypothesis that the OR rate was 10% against an alternate hypothesis that it was 30%, with a power of 0.9 and type I error probability of 0.1. Two ORs from the first 16 patients recruited into stage I were required to continue recruitment into a second stage. At least five ORs from the total of 25 patients were required to conclude that selumetinib showed evidence of efficacy using the per-protocol population.

OR rate was reported as the proportion of patients who achieved OR out of the total per-protocol population, with 90% confidence intervals (CI). Sensitivity analyses were also performed to compare this OR rate with ones that used the MTD population and the any-dose population as the denominator. The OR rate was also calculated for those who received at least six cycles of selumetinib.

The distribution of the number of selumetinib cycles was reported by median and range, along with the proportion of patients who completed 6 cycles. Reasons for discontinuation were reported in relation to the number of cycles received.

PFS was analysed for the per-protocol population using the Kaplan-Meier method with curves presented alongside estimates of the median, the 3- and 6-month rates with 90% CI.

HIV control was assessed by plotting longitudinal HIV-1 viral load and CD4 cell counts over time for the per-protocol population. Due to missing data at the pre-treatment cycle 1 day 2 visit, the CD4 cell counts from the screening visit were used as the baseline comparator. Paired t-tests were done to test the difference between CD4 cell counts at screening, cycle 3 day 22 and cycle 6 day 22. Due to multiple testing, a Bonferroni correction was applied to the significance threshold, calculated as 0.05 ÷ 2 = 0.025.

Pharmacodynamic measures were assessed by percentage changes in serum angiogenic markers and correlated with tumour response. The baseline used was that measured at cycle visit 1 day 1 pre-selumetinib treatment. Analysis of longitudinal data was performed using a mixed effects repeated measured regression, with a trend line and 90% CI derived using the fixed effect estimates from the model.

Date of database lock was 24-Aug-2020.

Analyses were performed using Stata 16.0.

An independent DMC reviewed interim data at the end of the phase I component of the trial to ensure patient safety and to agree on the MTD. There were no formal stopping rules.

## Results

**Fig. 1:**
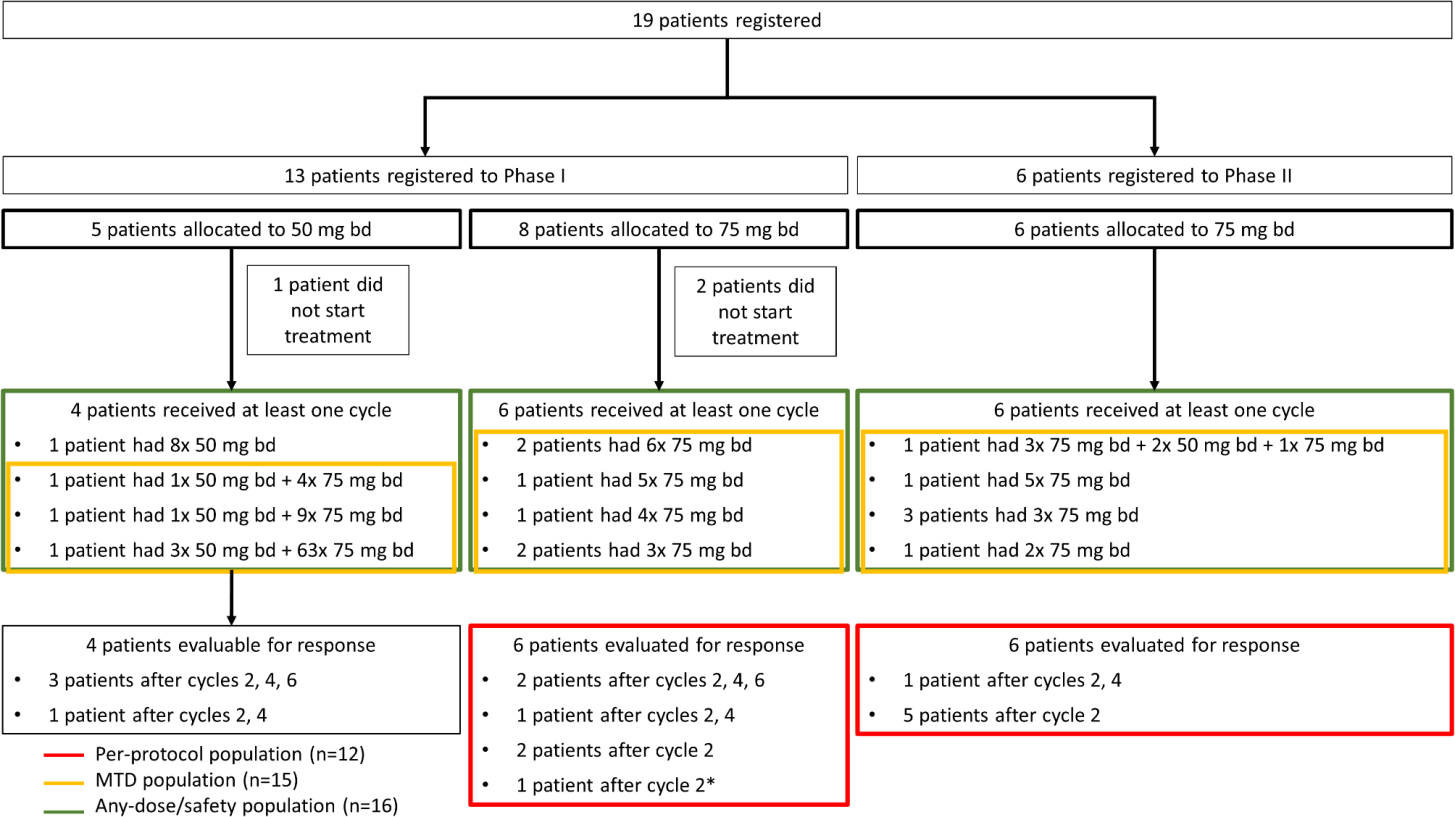
SCART trial profile. The figure shows patient recruitment, dose allocation, phase of trial recruited to, and patient-specific trial completions. All patients allocated to the maximum tolerated dose (MTD) of 75 mg were included in the phase II analyses of the trial. Total phase I patients: 10 (13 recruited^†^). Total phase II patients: 6. Total assessed at recommended phase II dose: 12. Total assessed for safety: 16. ^†^ Total excluded: 3 – one patient allocated to 50 mg bd was ineligible, one patient allocated to 75 mg bd was ineligible, and one allocated to 75 mg bd withdrew consent. None received any treatment * Disease evaluation for this patient used the ACTG criteria without additional clinical photographs

Between 15-Jun-2012 and 25-Sep-2018, 19 patients were recruited, 13 into phase I and 6 into phase II, of whom three in phase I did not start treatment and were not included in the final analysis (Fig. 1). Patient baseline characteristics of those who started treatment are described in Table 1. Median age was 44 years (range 33, 63) with 15/16 (94%) male, 13/16 (81%) performance status 0, and a median time from HIV diagnosis of 5.1 years (range 0.9, 25.4). Most patients had predominantly cutaneous disease, with one patient having a clinically significant abnormal CT, described as showing widespread pulmonary KS with irregular nodules, small volume axillary retroperitoneal lymph nodes and subcutaneous nodules (Table 1). A detailed list of the ART each patient was receiving at the time of registration is included in Supplementary Appendix 2. One patient in phase I died from disease-related causes having discontinued study treatment 251 days earlier due to disease progression.

**Table 1:** Patient baseline characteristics

	Dose Allocation			
	50 mg twice daily (*N* = 4)	75 mg twice daily (*N* = 12)		Overall (*N* = 16)
**Age (years)**				
Median	49	43		44
Range (min, max)	39, 63	33, 56		33, 63
**Sex [n (%)]**				
Male	4 (100.0)	11 (97.7)		15 (93.8)
Female	0 (0.0)	1 (8.3)		1 (6.3)
**Ethnicity [n (%)]**				
Caucasian	3 (75.0)	9 (75.0)		12 (75.0)
Black – African	1 (25.0)	2 (16.7)		3 (18.8)
Mixed	0 (0.0)	1 (8.3)		1 (6.3)
**WHO Performance Status [n (%)]**				
0	3 (75.0)	10 (83.3)		13 (81.3)
1	1 (25.0)	1 (8.3)		2 (12.5)
2	0 (0.0)	1 (8.3)		1 (6.3)
**Time from HIV Diagnosis to Trial Registration (years)**				
Median	10.2	4.2		5.1
Range (min, max)	4.4, 25.5	0.9, 9.4		0.9, 25.4
**Time from Starting ART to Trial Registration (years)**				
Median	1.8	3.4*		3.3^&^
Range (min, max)	0.8, 4.2	0.3, 5.9*		0.3, 5.9^&^
**Viral Load (copies/ml)**				
Median	40	40		40
Range (min, max)	40, 40	0, 108		0, 108
**CD4 Count (cell/mm** ^**3**^ **)**				
Median	503.0	552.0		549.0
Range (min, max)	129.0, 1247.0	138.0, 934.0		129.0, 1247.0
**Kaposi Sarcoma Burden**				
- **Kaposi sarcoma CT scan examination [n (%)]**				
Normal	1 (25.0)	7 (58.3)		8 (50.0)
Abnormal clinically insignificant	2 (50.0)	5 (41.7)		7 (43.8)
Abnormal clinically significant	1 (25.0)	0 (0.0)		1 (6.3)
- **Number of cutaneous lesions [n (%)]**				
*≤* 10	1 (25.0)	2 (16.7)		3 (18.8)
> 10 and *≤* 50	2 (50.0)	7 (58.3)		9 (56.3)
> 50	1 (25.0)	2 (16.7)		3 (18.8)
Missing	0 (0.0)	1 (8.3)		1 (6.3)
**Previous Systemic Treatment for Kaposi Sarcoma [n (%)]**				
Chemotherapy	2 (50.0)	4 (33.3)		6 (37.5)
Radiotherapy	1 (25.0)	0 (0.0)		1 (6.3)
Chemo- and radiotherapy	0 (0.0)	2 (16.7)		2 (12.5)
Chemotherapy and surgery	0 (0.0)	2 (16.7)		2 (12.5)
Missing	1 (25.0)	4 (33.3)		5 (31.3)

* Start date for one patient’s ART was not recorded. Therefore, *N* = 11 for these data

^&^ Start date for one patient’s ART was not recorded. Therefore, *N* = 15 for these data

Percentages may not add up to 100 due to rounding

ART, antiretroviral therapy

Of the ten patients treated within phase I, four were allocated to the initial 50 mg bd dose, and six allocated to the 75 mg bd dose (Fig. 1). Of the four patients who started on 50 mg bd, two patients had their doses increased to 75 mg bd at cycle 2, one patient had their dose increased to 75 mg bd at cycle 4, and one patient received the 50 mg bd dose only (Fig. 1). None experienced a DLT. In the first cohort of patients who started selumetinib at 75 mg bd, one DLT was observed. This led to an expansion of the cohort at this dose level to a total of six patients. The DLT consisted of asymptomatic concurrent increase in serum amylase and raised eosinophils, both grade 3, which were categorised as a single event and possibly related to treatment and resolved with no sequelae. No further DLTs were reported. In addition, no clinically significant changes were observed in circulating ART levels, and plasma levels of selumetinib and the active N-desmethyl metabolite levels were consistent with published reports from other selumetinib studies (Supplementary Appendix 5) [19–22].

In February 2014, the SRC concluded that selumetinib at 75 mg bd should be defined as the MTD/RP2D as it was the dose for which 1 out of 6 DLTs were observed and, as the trial design specified, the 100 mg bd dose level would only be tested if no DLTs were observed at the lower dose levels. This decision was ratified by the DMC in March 2014, and they approved continued recruitment into phase II. This MTD/RP2D was in accordance with the recommended treatment dose in monotherapy and combination studies in other advanced cancers [23–26].

A further ten participants were planned to be treated at the RP2D to analyse stage one of the Simon’s two stage trial design to evaluate treatment response. However, only six additional participants were treated before the study closed due to slow recruitment.

**Fig. 2:**
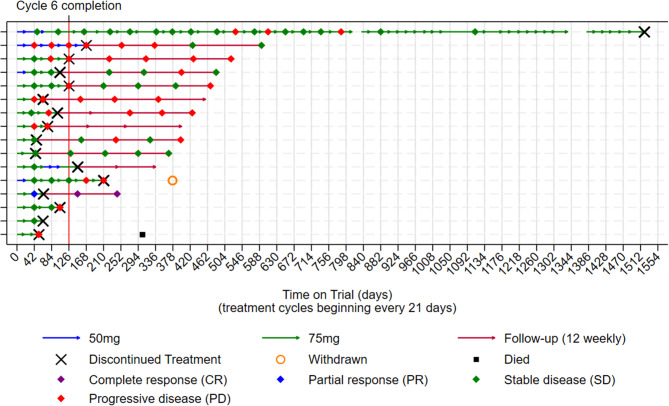
Patients’ trial journey. One patient allocated to 50 mg bd was ineligible, one patient allocated to 75 mg bd was ineligible, and one allocated to 75 mg bd withdrew consent. None received any treatment and are therefore not presented in this figure. Line interruptions are due to missing data at these time points. No treatment discontinuations or deviations were recorded at these time points

Table 2 presents the list of trial treatments completed, dose compliance, and the number of cycles completed for each patient. Across all doses given, the median number of completed treatment cycles was 5 (range 2–60). Six participants (37.5%) completed the scheduled six cycles of study treatment; three starting selumetinib at 50 mg bd, and three starting selumetinib at 75 mg bd. One participant on a starting dose of selumetinib 50 mg bd discontinued treatment after 5 cycles due to unacceptable toxicity having dose escalated to 75 mg bd from cycle 2, whilst five participants starting selumetinib at 75 mg bd discontinued treatment before completing the six scheduled cycles due to unacceptable toxicity. Four patients discontinued treatment before completing six cycles due to progressive disease (Table 2). Of note, one participant receiving selumetinib 50 mg bd experienced prolonged stable disease (SD) as best response, which was maintained over 25 cycles before developing progressing disease, and went on to complete a total of 60 cycles of treatment with clinical benefit before eventually discontinuing treatment. Response evaluations over time in relation to the treatment received for each patient are shown in Fig. 2.

**Table 2:** Patient treatments and outcomes

Duration on Trial (Days)	Starting Dose (mg bd)	Phase II Eligible	Cycles Completed	Mean Compliance (%)	Reason for discontinuing treatment
245	50	No	10	99.6	Patient choice (long journey time)
506	50	No	5	100.0	Toxicity
609	50	No	8	100.0	Completed scheduled 6 treatment cycles
1945	50	No	60	97.6	Progressive disease
68	75	Yes	3	99.7	Progressive disease
90	75	Yes	3	97.6	Toxicity
111	75	Yes	5	100.0	Progressive disease
224	75	Yes	3	81.0	Toxicity
345	75	Yes	6	97.6	Toxicity
379	75	Yes	2	96.4	Toxicity
418	75	Yes	3	73.2	Toxicity
426	75	Yes	4	86.9	Progressive disease
448	75	Yes	5	86.5	Toxicity
477	75	Yes	3	99.2	Progressive disease
498	75	Yes	6	98.0	Completed scheduled 6 treatment cycles
538	75	Yes	6	100.0	Completed scheduled 6 treatment cycles

Note: One patient allocated to 50 mg bd was ineligible, one patient allocated to 75 mg bd was ineligible, and one allocated to 75 mg bd withdrew consent. None received any treatment and are therefore not presented in this table

**Fig. 3:**
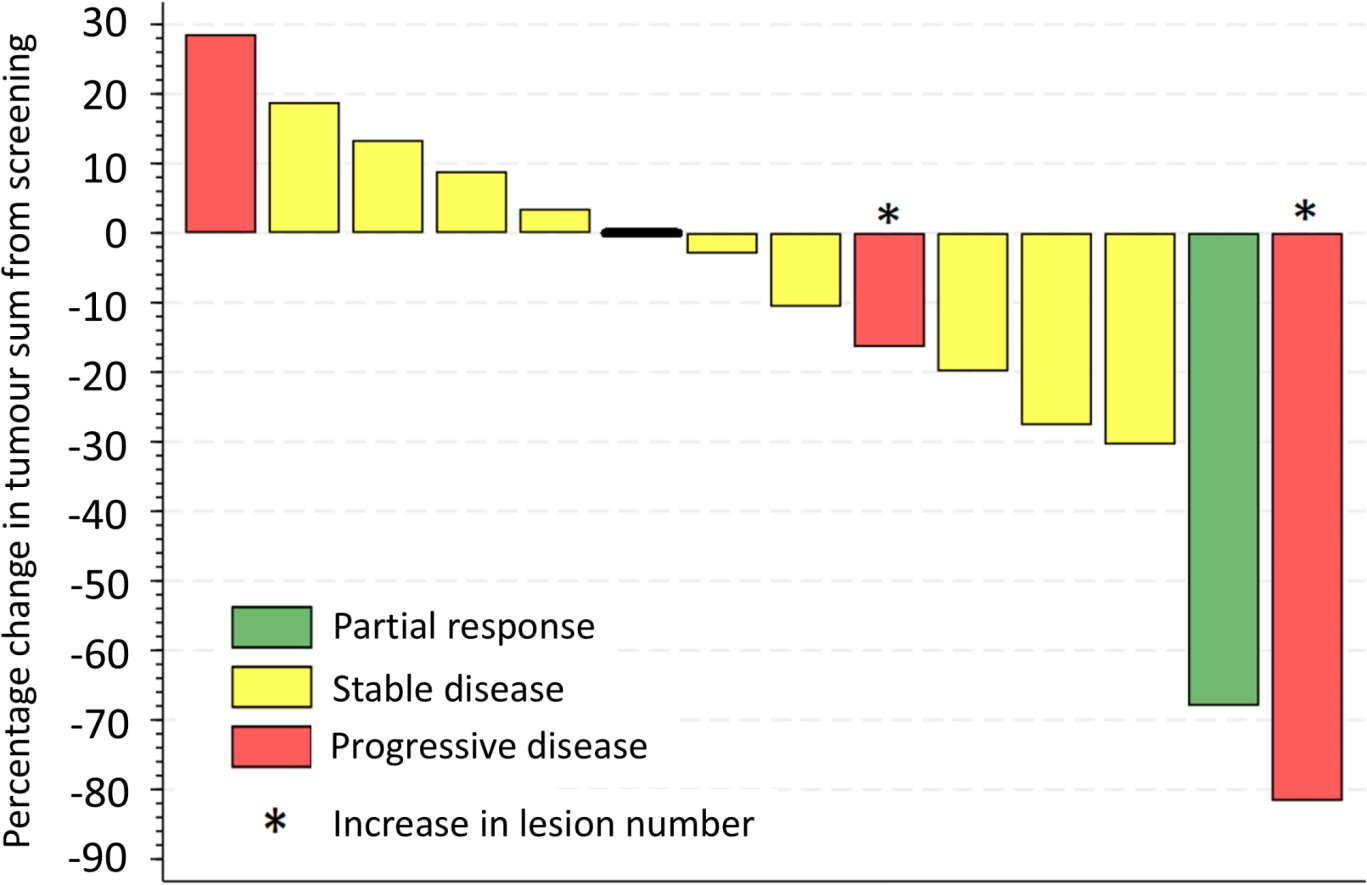
Best clinical response. Waterfall plot showing best clinical response recorded during the planned 6 treatment cycles in the any-dose/safety population. Fourteen of 16 patients are shown; two were omitted from this plot due to incomplete measurements; one with reported stable disease, one with progressive disease. Note two patients, both marked with *, one with stable disease and one with partial response in target lesions are marked as progressive disease as per ACTG criteria due to an increase in (off-target) lesion number. Overall, 1/16 partial response, 11/16 stable disease, 3/16 progressive disease as best response

Of the 12 patients analysed per protocol, only one had an OR which was a PR giving an OR rate of 8.3% (90% CI: 0.4%, 33.9%). Sensitivity analysis gave an OR rate of 6.7% (90%: 0.3–27.9) for the MTD population and 6.3% (90% CI: 0.3–26.4) for the any-dose/safety population. The only patient with a reported PR discontinued treatment after 3 cycles due to AEs but despite discontinuing treatment the response deepened to a CR and a response duration of 201 days was recorded. Due to the limited amount of data, no further DoR analyses were feasible. The clinical benefit rate (CR, PR and SD) in the any-dose/safety population was 12/16 (75%) (Fig. 3); the change in total tumour area during the course of treatment for the any-dose/safety population is shown in Supplementary Appendix 6. The 6-month PFS rate in the per-protocol population was 38% (90% CI: 16%, 61%) (Table 3).

**Table 3:** Progression-free survival times

	3-month PFS (90% CI)	6-month PFS (90% CI)	Median PFS in months (90% CI)
Per protocol*	57%(31%,76%)	38%(16%, 61%)	3.42 (1.74, -^$^)
MTD^#^	66%(42%,82%)	44% (22%, 64%)	5.53 (2.53, 12.80)
Any dose/safety^&^	62%(39%,78%)	41% (21%, 61%)	4.14 (2.53, 12.80)

CI, confidence intervals; MTD, maximum tolerated dose; PFS, progression-free survival time

*12 patients were included in the per protocol population

^#^ 15 patients were included in the MTD population

^&^ 16 patients were included in the any dose/safety population

^$^ The Kaplan-Meier curve for the upper limit of the 90% CI for the median did not reach 0.5

One patient experienced one serious adverse reaction (SAR) and one serious adverse event (SAE). The SAR comprised multiple events; the patient was hospitalised with anaemia and a non-skin related infection, both grade 3 events considered at least possibly related to trial treatment. The same patient also experienced an SAE of spinal cord compression (grade 4), along with faecal incontinence (grade 2) and tumour pain (grade 3), which were reported as unrelated to study treatment. A second patient experienced a grade 3 infection (cellulitis), which was considered possibly related to study treatment and was therefore reported as an SAR.

All 16 patients experienced at least one adverse event (AE). In addition to the DLT, two SARs and one SAE, there were a further 366 AEs reported, with the majority (98%) of events categorised as grade 1 or 2 and 69% deemed as ARs (Supplementary Appendix 7). The six grade 3 AEs that occurred in five separate patients included three ARs, all rash acneiform: one patient recorded an AR in cycle 3 of 75 mg bd selumetinib, which resolved with no sequelae; a second patient recorded an AR in cycle 1 of 75 mg bd selumetinib, which resolved with sequelae; and a third also an the AR in cycle 1 of 75 mg bd selumetinib, which resolved with sequelae. The other grade 3 events were increased creatine phosphokinase in one patient, and increased alkaline phosphatase and tumour pain in another. Of the AEs graded 1 or 2, the most common was patients experiencing a decrease in their red blood cell count (22 AEs experienced by seven patients; see Supplementary Appendix 8).

**Fig. 4:**
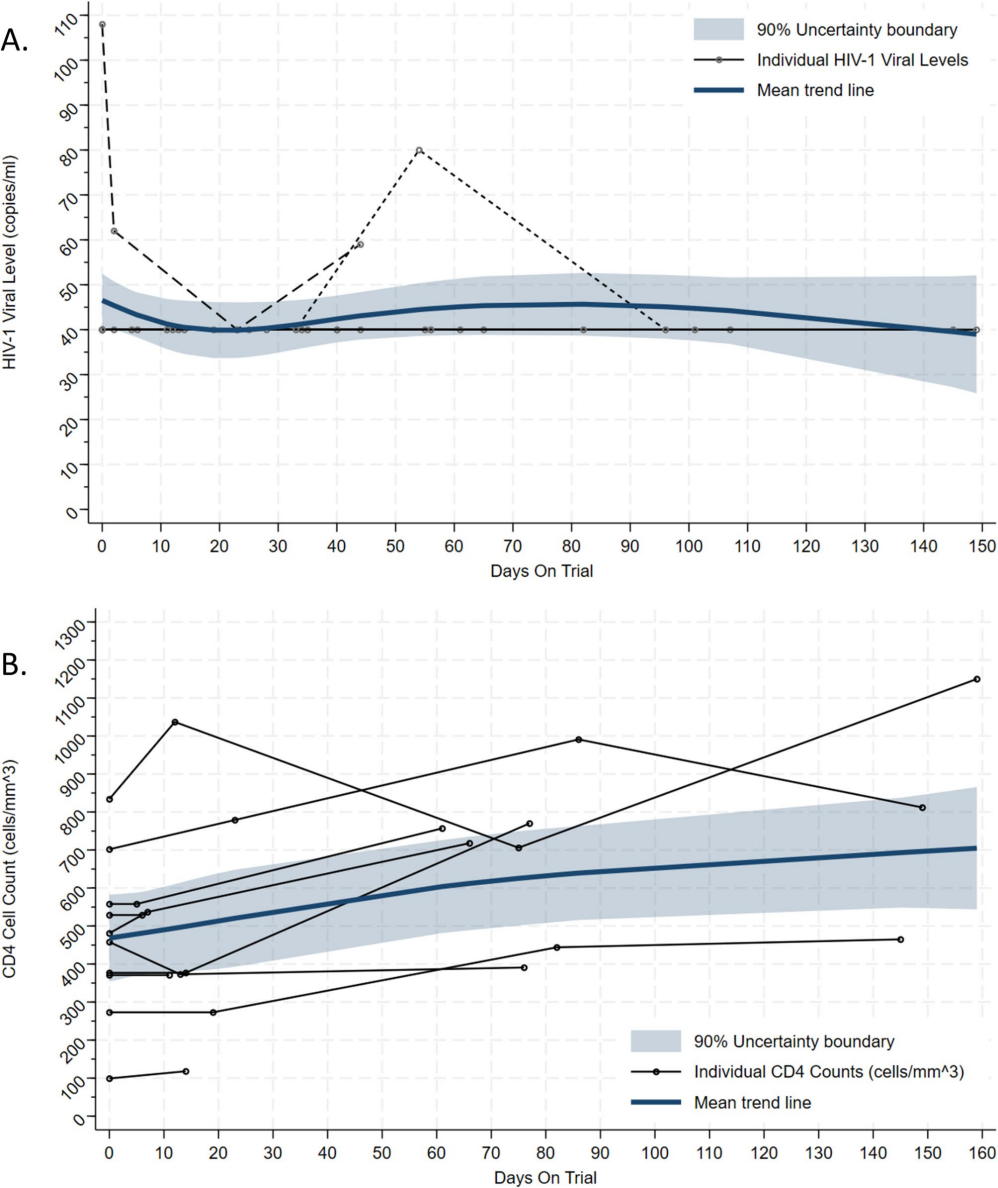
HIV-1 viral load and CD4 cell counts. Panel A shows HIV-1 viral load (copies/ml) measurements. Panel B shows CD4 cell count (cells/mm^3^) measurements. Trend line and 90% confidence intervals have been added. Those data from patients that fell outside of the mean trend line are shown as individual dashed lines. The per protocol population was used i.e., the response evaluable phase II patients (including those transferred over from phase I) who started selumetinib at the recommended phase II dose and received at least one cycle Note: dashed lines in A represent two patients who levels did not remain at 40 copies/ml

During the trial, HIV-1 viral load remained undetectable in patients receiving selumetinib (Fig. 4a). However, at cycle 3 day 22, a mean increase in CD4 cell counts of 161.27 cells/mm^3^ (95% CI: 41.91, 280.63; *p* = 0.013) was observed, this remained constant during the following cycles of treatment where, at cycle 6 day 22, an increase of 152.67 cells/mm^3^ (95% CI: 31.87, 273.47; *p* = 0.023) was observed compared to pre-treatment levels (Fig. 4b).

**Fig. 5:**
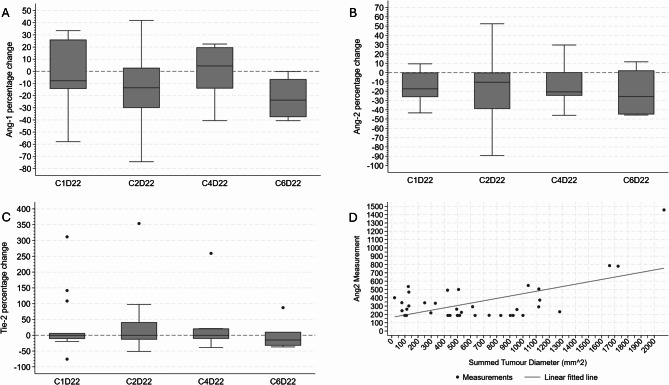
Pharmacodynamic changes. Box and whisker plots showing median with interquartile ranges of percentage change for the angiogenic markers Ang-1 (**A**), Ang-2 (**B**) and Tie-2 (**C**) at day 22 during cycles 1, 2, 4 and 6 compared to levels at pre-dose cycle 1, day 1 for those patients receiving any dose of selumetinib treatment. Panel D shows the correlation between levels of Ang-2 and tumour burden; Pearson correlation = 0.5902

Unfortunately, due to the incomplete collection of biopsy tissue and PBMC samples, only limited pharmacodynamic analyses were possible. Whilst serum levels of the angiogenic marker Ang-2 tended to be lower on selumetinib treatment compared to baseline measurements, no significant changes in the circulating levels of Ang-1, Ang-2, or Tie-2 were observed at the end of cycles 1, 2, 4 and 6 compared to levels at pre-dose cycle 1, day 1 (Fig. 5a-c). However, a positive relationship between the levels of Ang-2 and the tumour burden was observed (Pearson correlation = 0.5902) (Fig. 5d). Using an unplanned mixed effects model analyses, for every unit increase in Ang-2, the summed tumour diameter increased on average by 0.667 mm^2^ (95% CI: 0.263, 1.072; *p* = 0.001) with all other variables in the model constant.

## Discussion

The SCART trial was a small, single-arm, phase I/II clinical trial to define the dose of selumetinib to use in combination with ART and to assess safety and preliminary efficacy. The study closed prematurely due to slow recruitment and thus limits the conclusions that can be drawn. However, the phase I primary objective was met, defining 75 mg bd as the RP2D. Pharmacodynamic analysis demonstrated a positive correlation between the angiogenic marker Ang-2 and tumour burden, with improvements in CD4 cell counts observed and no detrimental impact on ART drug levels or HIV viral load. However, only one patient reported an OR, and therefore, we cannot recommend the use of selumetinib at 75 mg bd in combination with ART in patients with HIV-associated KS.

Although initially performing well, recruitment to the SCART trial was challenging, and as a result, SCART was closed before reaching the planned recruitment target. The difficulties in recruitment were partly due to the rarity of refractory HIV-associated KS because of improvements in HIV care. Additionally, the study design included stringent eligibility criteria including well controlled HIV; specifically, patients with poorly controlled HIV and active opportunistic infections were excluded. These ensured patient safety and the inclusion of patients in clinical need of new treatment but also narrowed the available patient population. Due to the rarity of the study population and the already available safety data on selumetinib at the time of the trial’s development [12, 23], a decision was made to employ a short dose escalation phase in the study design, and DLTs were restricted to grade ≥ 3 toxicities reported during the first treatment cycle of three weeks. However, a large number of predominantly grade 1 and grade 2 AEs were reported, and 37.5% of participants (6/16) discontinued selumetinib due to toxicity before completing six cycles of treatment. Furthermore, several potentially eligible patients declined study entry due to their concerns around the risk of experiencing additional treatment-related toxicity. Taken together, this suggests that multiple lower-grade toxicities were not acceptable within this relatively young, fit, patient population. The phase I design focused on PK data and short-term toxicities to establish the MTD and RP2D, but on reflection, evaluation of longer-term toxicities and patient reported outcomes measures, rarely included in phase I oncology trials [24], may have been valuable. With hindsight, the 50 mg bd dose level may have been a more acceptable dose to test in phase II.

Importantly, no adverse impact on HIV control was observed during SCART. Indeed, CD4 cell counts overall increased during selumetinib treatment. A CD4 cell count increase is anticipated in HIV patients with first initiation of ART. However, the inclusion criteria restricted patients to SCART with well controlled HIV; patients established on ART for a minimum of three months were included, whilst those with HIV viral load > 200 copies/ml and those with active opportunistic infection were excluded. On average, patients in SCART had been established on their ART regimen for three years before trial entry. We therefore conclude that the increase in CD4 cell count observed on study was an effect of selumetinib treatment although further studies would be needed to confirm this finding. Furthermore, MEK inhibitors can reduce RNA virus replication and reduce viral hyperinflammation [25], and thus may be of benefit in the treatment of HIV and other RNA viruses such as SARS-CoV-2.

The inclusion of ART drug level monitoring within SCART was a major strength of the study. Drug interactions with ART are common and can be detrimental. Cancer represents a significant cause of increased morbidity and mortality in people living with HIV [26]. However, this patient population is frequently excluded from oncology trials, and even studies within this patient population do not usually formally evaluate the impact of cancer treatment on ART drug levels.

Previous studies have suggested that circulating Ang-2 correlates with the burden of KS disease [11]; our study also reports this. A trend towards decreased Ang-2 with selumetinib treatment was observed, supporting the hypothesis that targeting the Ras/Raf/MEK/ERK pathway would influence Ang-2 expression. However, only one patient reported an objective tumour response. Of note, response reporting in cutaneous KS is particularly challenging. Cutaneous lesions can’t be assessed using standard RECIST methods. We adopted the reporting criteria developed by the AIDS Clinical Trials Group, which stipulated a > 50% reduction in the number and/or size of KS lesions for a partial response, and response evaluation also considered tumour nodularity and tumour-associated oedema. As shown in the waterfall plot (Fig. 3), responses insufficient to meet the requirements for a partial response were observed in several patients, with eleven patients reporting SD as best response.

Collection of paired tumour biopsies for translational studies were planned, but unfortunately the quality and quantity of the samples received were insufficient to allow analysis of cell signalling changes in response to selumetinib treatment. Of note, no changes were observed in circulating Tie-2 levels in response to selumetinib.

## Conclusions

In summary, whilst we cannot recommend further evaluation of selumetinib 75 mg bd as monotherapy in HIV-associated KS in combination with ART, the clinical benefit rate, improvements in CD4 cell count, and evidence of response in Ang-2 suggest that studies exploring selumetinib in combination with other agents including anti-angiogenic agents and/or immune checkpoint inhibitors are warranted.

## Electronic supplementary material

Below is the link to the electronic supplementary material.

Supplementary appendix 1. SCART trial protocol (ultimately version 12.0, dated 03-Feb-2017)

Supplementary appendix 2. Patients’ antiretroviral therapy at time of trial registration

Supplementary appendix 3. The AIDS Clinical Trials Group (ACTG) criteria

Supplementary appendix 4. Description of the pharmacokinetic and pharmacodynamic assessments, and sample collection and processing methods

Supplementary appendix 5. Antiretroviral drug levels, as well as selumetinib and N-desmethyl selumetinib levels in plasma collected as part of the phase I pharmacokinetic analysis

Supplementary appendix 6. Change in total tumour area during the course of treatment for the any-dose/safety population

Supplementary appendix 7. Number of patients experiencing adverse events and adverse reactions who received any dose of selumetinib

Supplementary appendix 8. Number of grade < 2 adverse events reported

## Data Availability

Participant data and the associated supporting documentation will be available within six months after the publication of this manuscript. Details of our data request process is available on the CRCTU website: https://www.birmingham.ac.uk/research/centres-institutes/cancer-research-uk-clinical-trials-unit/data-sharing-policy Only scientifically sound proposals from appropriately qualified research groups will be considered for data sharing. The decision to release data will be made by the CRCTU Director’s Committee, who will consider the scientific validity of the request, the qualifications and resources of the research group, the views of the Chief Investigator and the trial steering committee, consent arrangements, the practicality of anonymising the requested data and contractual obligations. A data sharing agreement will cover the terms and conditions of the release of trial data and will include publication requirements, authorship and acknowledgements and obligations for the responsible use of data. An anonymised encrypted dataset will be transferred directly using a secure method and in accordance with the University of Birmingham’s IT guidance on encryption of data sets.

## Abbreviations

ACTG: AIDS Clinical Trials Group
ANC: absolute neutrophil count
AR: adverse reaction
Bd: twice daily
BLQ: below the level of quantification
ART: antiretroviral therapy
CI: confidence intervals
CR: complete response
CRCTU: Cancer Research UK Clinical Trials Unit
DLT: dose limiting toxicities
DMC: data monitoring committee
DoR: duration of response
ECMC: Experimental Cancer Medicine Centres
HHV-8: human herpesvirus-8
HSHV: Kaposi sarcoma herpesvirus
KS: Kaposi sarcoma
MEK: mitogen-activated protein kinase kinase
MTD: maximum tolerated dose
NA: no assessment
Od: once daily
OR: objective response
PFS: progression-free survival time
PR: partial response
RP2D: recommended phase II dose
SAE: serious adverse event
SD: stable disease
vGPCR: viral G protein-coupled receptor
