# Supplementary material for: Selumetinib in Combination with Anti Retroviral Therapy in HIV-associated Kaposi sarcoma (SCART): an open-label, multicentre, phase I/II trial
Source: BMC Cancer. 2025 Mar 19;25:505. doi: 10.1186/s12885-025-13890-x (PMC11921695; doi:10.1186/s12885-025-13890-x)
Supplement: Supplementary file 1 — Supplementary appendix 1. SCART trial protocol (ultimately version 12.0, dated 03-Feb-2017) [file 12885_2025_13890_MOESM1_ESM.pdf]

Supplementary appendix 1 – SCART protocol

SCART

Phase I/II study of oral MEK inhibitor Selumetinib (AZD6244 Hyd-Sulphate) in Combination with Highly Active Anti-Retroviral Therapy (HAART) in AIDS-associated Kaposi's sarcoma (KS).

Protocol Version No.: 12.0

Protocol Version Date: 03_Feb_2017

Protocol No.: SA1001

EudraCT No.: 2011-003099-35

Sponsor No.: STH16059

Cancer Research UK Trial Number: CRUKD/11/005

AMENDMENTS

The following amendments and/or administrative changes have been made to this protocol since the implementation of the first approved version.

Amendment number	Date of amendment	Protocol version number	Type of amendment	Summary of amendment
1	15th Dec 2011	2.0	Substantial amendment	Rewording or amendment of text for clarification in the Treatment Details and Translational Research sections of the protocol.
N/A	1st Feb 2012	2.0a	Non-substantial amendment	Change in patient registration contact details
3	30th March 2012	3.0	Substantial amendment	• Trial Monitor contact details removed. Trials in the QA team are dealt with by various personnel within the team.<br>• Wording of exclusion criteria changed with reference to hepatitis B and C (section 4.2).<br>• Clotting Studies clarified in text and assessment table (section 7)<br>• Instructions for sites to send clinical photographs to the trial office mailbox added to section 7.10.2.<br>• Statistical section (13.2.2) has been redrafted for clarity.
N/A	12th June 2012	3.0a	Non-substantial amendment	Change in SCART Trial Coordinator's job title and telephone number
4	11th October 2012	4.0	Substantial amendment	• Updated Contact Details<br>• Clarified dose cohort safety teleconference (3.1)<br>• Updated information regarding the continuation of patients on treatment past 12 months (3.1 & 3.2).<br>• Amended Inclusion criterion to provide clarity (4.1)<br>• Updated the Informed Consent Procedure (5.2).<br>• Updated link for HAART drug interactions (7.8)<br>• Additional text added regarding HAART regimen and interactions with CYP3A (7.8)

				• Clarified the histology assessment (7.9.1)<br>• ECHO/MUGA, Ophthalmology assessment and tumour biopsy moved from within 2 weeks of treatment to within 4 weeks of treatment (7.9.6 & 7.9.7).<br>• Corrected error in Phase II assessment table (7.9.7)<br>• Clarified abnormal laboratory findings as adverse events (9.1.1)<br>• Updated Case Report Form table (10.1).<br>• Inserted the NHYA classification (Appendix 5).
5	26th February 2013	5.0	Substantial amendment	• Alter eligibility criteria to include patients on Atazanavir, who have asymptomatic elevated levels of bilirubin, but normal liver function (4.1)<br>• Clarify procedure for gaining approval from AstraZeneca for patient continuation past the standard 6 cycles of treatment<br>• Additional instructions added requesting that patients do not change HAART medication unless clinically necessary.
N/A	29th May 2013	5.0a	Non-substantial amendment	• Text and patient schedule changed for consistency as approved in amendment 4: ECHO/MUGA, Ophthalmology assessment and tumour biopsy moved from within 2 weeks of treatment to within 4 weeks of treatment (7.9.1).
6	2nd October 2013	6.0	Substantial amendment	• Updated Contact Details; Dr Young and Professor Dockrell named as Clinical Coordinator and Deputy Clinical Coordinator, respectively. Trial Coordinator and Trial Administrator updated accordingly.<br>• Addition to and amendment of text regarding the expedited reporting of DLTs (sections 3.1.1.2 and 7.4).<br>• Additional text added regarding the prompt return of Cycle 1 CRF copies (section 10).

				• Corrected a minor error regarding the PBMC sample collection to match the Laboratory Manual.<br>• Minor formatting of reference section
7	9th December 2013	7.0	Substantial amendment	• Altered eligibility criteria to exclude patients of Japanese ethnicity (section 4.2)<br>• Metabolic side effects updated to include hypoalbuminaemia (section 7.3.1).<br>• Date of Amendment 6 added to table<br>• Updated contact details for Registration of patients
8	10th December 2014	8.0	Substantial Amendment	• Updated Trial Contacts details.<br>• Amended page numbering.<br>• Clarified that progression free survival data will be collected for each patient for 6 months from commencing treatment for analysis and for 12 months from completing treatment as supportive data (trial synopsis and sections 2.1.2, 2.2.2, 12, 13.2.1 and 13.2.2).<br>• Removed Japanese ethnicity from exclusion criteria (trial synopsis and section 4.2).<br>• Amended trial duration<br>• Rearranged a sentence to clarify the conditions required to raise the dose to level 3 during phase I (section 3.1).<br>• Additional text added to phase I section to include DLT recording window and reflect that the MTD for selumetinib has been found as part of the completion of phase I (section 3.1.1 and 3.1.2).<br>• Amended exclusion criteria as per AstraZeneca's guidance (4.2).<br>• Added a section on the eligibility of Asian patients (section 4.2.1).<br>• Added a section on Asian Pharmacokinetic Data Associated with Selumetinib (section 7.3.1.1).

				• Amended text regarding vitamin E exposure (section 7.8).<br>• Removed phase I assessments list and schedule table and amended text to reflect the successful completion of phase I (Section 7.9.2.1 and 7.9.7).<br>• Added windows for visits in phase II (section 7, 8 and schedule table).<br>• Clarified assessments patients should undergo when discontinuing selumetinib treatment (section 7.9.2.2, 7.9.3, 7.9.4, 7.9.5 and 7.9.6).<br>• Clarified definition of the follow-up period (section 7.11).<br>• Clarified reporting requirements for adverse events for conditions which change CTCAE grade since the previous visit (section 9.2.1.1).<br>• Added Ad hoc Haematology/ Clinical Chemistry Assessments Form and Post Treatment Adverse Event Follow-Up Form to CRF table (section 10.1).<br>• Clarified end of trial definition (section 12).<br>• Changed Trial Steering Committee to Safety Review Committee (section 14.4 and all other references in text).<br>• Added Frequency of DMC meetings (section 14.5) and amended a spelling mistake.<br>• Correction of grammatical, formatting/ spelling errors.<br>• Added AEs of interstitial lung disease-like events and updated information regarding visual events (7.3.1).<br>• Updated guidance for management of LVEF, dyspnoea, vision disorders, CK, diarrhoea and rashes (Appendix 7, 8, 9, 10, 11 & 12).
10	29th June 2015	9.0	Substantial Amendment	• Updated Trial Contacts details<br>• Changed ECOG to WHO in line with IRAS form<br>• Updated Trial Office Contact Details

				• Removed registration of patients by email from Trial Entry. Registration is by phone or Fax and updated telephone number for registration (section 6)<br>• Updated SCART Trial office telephone number for Dose Modification (section 7.4)<br>• Added note to check Appendix 10 after Non-Haematologic Adverse Events section regarding elevated CK (section 7.4.2)<br>• Added bullet point to 7.9.6 Post-Treatment Follow-Up: CT scans every 12 weeks for 12 months post end of treatment if visceral or extensive disease identified on baseline CT.<br>• Added sentence to 7.11 Patient Follow Up: plus additional CT scans if visceral or extensive disease identified on baseline CT.
11	01st Dec 2015	10.0	Substantial Amendment	• Removal of Co-Investigator from Trial Contacts and addition of Senior Trial Manager<br>• Removal of HIV physician from trial management team (section 3.1.1.3)<br>• Added note clarifying reasons for discontinuation of treatment after one year: if disease progression and in opinion of Chief Investigator (sections 3.2 and 7.2)<br>• Added bullet point to 7.8 Conmeds about interaction of grapefruit juice and St John's wort with HAART and selumetinib
12	10-Mar-2016	11.0	Substantial Amendment	• Change of Chief Investigator
13	03-Feb-2017	12	Substantial Amendment	• Change of End of Trial definition

TRIAL CONTACTS

**Chief Investigator:**<br>**Dr Robin Young**<br>Weston Park Hospital<br>Whitham Rd<br>Sheffield<br>S10 2SJ<br>Phone: 0114 271 2133<br>Fax: 0114 2265242<br>E-mail: r.j.young@sheffield.ac.uk	**Co-investigator (Deputy Clinical Coordinator):**<br>**Prof David Dockrell**<br>Department of Infection and Immunity<br>The Medical School<br>Beech Hill Road<br>Sheffield S10 2RX<br>Phone: 0114 2261427<br>Fax: 0114 2268898<br>E-mail: d.h.dockrell@sheffield.ac.uk
**CRCTU Lead**<br>**Prof Lucinda Billingham**<br>Cancer Research UK Clinical Trials Unit (CRCTU)<br>Institute of Cancer and Genomic Sciences, Vincent Drive,<br>University of Birmingham<br>Edgbaston B15 2TT<br>Phone: 0121 414 3790<br>Email: l.j.billingham@bham.ac.uk	**Senior Trial Manager**<br>**Dr Laura Llewellyn**<br>Cancer Research UK Clinical Trials Unit<br>Institute of Cancer and Genomic Sciences, Vincent Drive,<br>University of Birmingham<br>Edgbaston B15 2TT<br>Phone: 0121 414 8421<br>Fax: 0121 4143529<br>E-mail: L.R.Llewellyn@bham.ac.uk
**Trial Coordinator:**<br>**Dr Gillian McNab**<br>Cancer Research UK Clinical Trials Unit<br>Institute of Cancer and Genomic Sciences, Vincent Drive,<br>University of Birmingham<br>Edgbaston B15 2TT<br>Phone: 0121 414 6788<br>Fax: 0121 4143529<br>E-mail: g.l.mcnab.1@bham.ac.uk	**Trial Administrator**<br>**Mark Gallagher**<br>Cancer Research UK Clinical Trials Unit<br>Institute of Cancer and Genomic Sciences, Vincent Drive,<br>University of Birmingham<br>Edgbaston B15 2TT<br>Phone: 0121 414 6788<br>Fax: 0121 4143529<br>E-mail: m.gallagher@bham.ac.uk
**Biostatistician**<br>**Amanda Kirkham**<br>Cancer Research UK Clinical Trials Unit,<br>Institute of Cancer and Genomic Sciences, Vincent Drive,<br>University of Birmingham<br>Edgbaston B15 2TT<br>Phone: 0121 4148876<br>E-mail: a.j.kirkham@bham.ac.uk	**Sponsor:**<br>**Sheffield Teaching Hospital NHS Foundation Trust**<br>D Floor,<br>Royal Hallamshire Hospital, Glossop Road, Sheffield S10 2JF

Coordinating Centre:
SCART Trial Office
Cancer Research UK Clinical Trials Unit

Institute of Cancer and Genomic Sciences, Vincent Drive,
University of Birmingham
Edgbaston B15 2TT
Email: SCART@contacts.bham.ac.uk

TRIAL SYNOPSIS

Title:

SCART – Phase I/II study of oral MEK inhibitor Selumetinib (AZD6244 Hyd-Sulphate) in Combination with Highly Active Anti-Retroviral Therapy (HAART) in AIDS-associated Kaposi's sarcoma (KS).

Trial Design:

An open-label multi-centre phase I/II trial.

Primary Objectives:

- To identify a safe dose for selumetinib in combination with HAART.
- To establish evidence of the efficacy of selumetinib in combination with HAART in patients with AIDS-associated KS.

Secondary Objectives:

- To describe the pharmacokinetics of selumetinib in combination with HAART, including the effects of selumetinib on HAART metabolism.
- To assess the toxicity of selumetinib in combination with HAART.
- To describe the pharmacodynamic effects of selumetinib in combination with HAART.
- Progression free survival rate at 6 months from commencing treatment.

Outcome Measures:

Phase I

- Selumetinib and metabolite serum levels.
- HAART drug levels.

Phase I & II

- Toxicity.
- Number of selumetinib cycles completed.
- Objective response rates.
- Progression free survival rate at 6 months from commencing treatment.
- Human immunodeficiency virus (HIV) control – viral load and CD4 counts.
- Pharmacodynamic measures of selumetinib in combination with HAART – serum angiogenic biomarker levels and pERK in tumour tissue.
- Analysis of peripheral blood mononuclear cells collected in a sub-study at selected centres.

Patient Population:

Patients with HIV-associated Kaposi's sarcoma.

Sample Size:

Expected recruitment of 12 patients to Phase I and 25 to Phase II.

Main Inclusion Criteria (not exhaustive)

- HIV positive and established on a HAART regimen for ≥ 3 months.
- Histologically confirmed KS with measurable disease.
- Progressive cutaneous or nodal KS not requiring chemotherapy OR progressive KS following cytotoxic chemotherapy.
- Adequate haematological, hepatic and renal function
- Age ≥ 18 years.
- World Health Organisation (WHO) performance status ≤ 2.

Main Exclusion Criteria (not exhaustive)

- HIV viral load > 200 copies/ml.
- Previous treatment with a Ras, Raf or MEK inhibitor.
- Active opportunistic infections.
- Clinical evidence of uncontrolled hypertension, heart failure, atrial fibrillation or unstable ischaemic heart disease.
- Evidence of any psychological, familial, sociological or geographical condition potentially hampering protocol compliance.
- Pregnant or breast-feeding women.

Trial Duration:

3 years including 12 month follow up period.

Trial Office Contact Details:

Gillian McNab
SCART Trial Co-ordinator
Cancer Research UK Clinical Trials Unit,
Institute of Cancer and Genomic Sciences
University of Birmingham,
Vincent Drive
Edgbaston,
Birmingham,
B15 2TT

Tel: 0121 414 6788

Fax: 0121 414 3529

Email: SCART@contacts.bham.ac.uk

ABBREVIATIONS

ACTG	AIDS Clinical Trials Group
AE	Adverse Event
AJCC	American Joint Committee on Cancer
ALP	Alkaline phosphatase
ALT	Alanine transaminase
Ang	Angiopoeitin
AST	Aspartate aminotransferase
AUC	Area under curve
bd	Bis die (twice a day)
BP	Blood pressure
CHI	Community Health Index
C_{max}	Maximal concentration
CR	Complete Response
CRCTU	Cancer Research UK Clinical Trials Unit
CT	Computerised Tomography
CTC	Common Terminology Criteria
CTCAE	Common Terminology Criteria for Adverse Events
CYP	Cytochrome P450
DCF	Dichlorofluorescein
DLT	Dose limiting toxicity
DMC	Data Monitoring Committee
DSUR	Development Safety Update Report
ECG	Electrocardiogram
ECOG	Eastern Co-operative Oncology Group
EGF	Epidermal growth factor
ELISA	Enzyme-linked immunosorbent assay
ERK	Extracellular signal regulated kinase
GCP	Good Clinical Practice
GP	General Practitioner
HAART	Highly active anti-retroviral therapy
HHV-8	Human herpesvirus-8
HIV	Human immunodeficiency virus
HRCT	High Resolution Computerised Tomography
ICF	Informed Consent Form
IGF	Insulin like growth factor
IL	Interleukin
IMP	Investigational Medicinal Product
INR	International Normalised Ratio
ISF	Investigator Site File
KS	Kaposi's sarcoma
LVEF	Left ventricular ejection fraction
MAPK	Mitogen-activated protein kinase
MEK	MAPK/extracellular signal-regulated kinase
mg	Milligram
MHRA	Medicines and Healthcare Products Regulatory Agency
MI	Myocardial Infarction
MTD	Maximum tolerated dose

MUGA	Multi gated acquisition
NHS	National Health Service
NNRTI	Non-nucleoside reverse transcriptase inhibitor
NRTI	Nucleoside reverse transcriptase inhibitor
NtRTI	Nucleotide reverse transcriptase inhibitor
NYHA	New York Heart Association
od	Omne in die (once daily)
PBMC	Peripheral Blood Mononuclear Cells
PD	Progressive disease
PDGF	Platelet derived growth factor
PFS	Progression free survival
PIS	Patient Information Sheet
PK	Pharmacokinetics
PLD	Pegylated liposomal doxorubicin
PO	Per oral
PR	Partial Response
REC	Research Ethics Committee
ROS	Reactive oxygen species
RPTD	Recommended phase II dose
SAE	Serious Adverse Event
SAR	Serious Adverse Reaction
SD	Stable disease
SUSAR	Suspected Unexpected Serious Adverse Reaction
TKI	Tyrosine kinase inhibitor
TLR	Toll-like receptor
TMG	Trial Management Group
TNF	Tumour necrosis factor
VEGF	Vascular endothelial growth factor
vGPCR	Viral G-protein coupled receptor
vIL	Viral interleukin

TABLE OF CONTENTS

1 Background and Rationale ... 15
1.1 HIV and KS ... 15
1.2 Mitogen-activated protein kinase (MAPK) Pathways and KS ... 15
1.3 Selumetinib ... 15
1.4 Highly Active Anti-Retroviral Therapy (HAART) ... 16
1.5 Trial Rationale ... 17
1.5.1 Justification for patient population ... 17
1.5.2 Justification for design ... 17
1.5.3 Choice of treatment ... 17
2 Aims, Objectives and Outcome Measures ... 18
2.1 Aims and Objectives ... 18
2.1.1 Primary Objectives ... 18
2.1.2 Secondary Objectives ... 18
2.2 Outcome Measures ... 18
2.2.1 Phase I ... 18
2.2.2 Phase I & II ... 18
3 Trial Design ... 19
3.1 Phase I ... 19
3.1.1 Dose Limiting Toxicity (DLT) ... 20
3.1.2 Maximum Tolerated Dose (MTD) ... 20
3.2 Phase II ... 20
4 Eligibility ... 21
4.1 Inclusion Criteria ... 21
4.2 Exclusion Criteria ... 21
4.2.1 Asian Patients ... 22
5 Patient Screening and Consent ... 22
5.1 Screening ... 22
5.1.1 Patient Screening Log ... 22
5.2 Informed Consent ... 22
6 Trial Entry ... 23
7 Treatment Details ... 23
7.1 Selumetinib ... 23
7.2 Treatment Schedule ... 23
7.3 Expected Toxicity ... 24
7.3.1 Adverse Events Associated With Selumetinib ... 24
7.3.2 Adverse Events Associated With HAART ... 26
7.3.3 Management of nausea and vomiting ... 26
7.3.4 Management of diarrhoea ... 26
7.5 Management of hypertension ... 26
7.3.5 Management of skin toxicities associated with Selumetinib ... 26
7.4 Dose Modifications ... 27
7.4.1 Haematologic Adverse Events ... 27
7.4.2 Non-Haematologic Adverse Events (including biochemistry/hepatic/renal function) ... 27
7.5 Treatment Compliance ... 27
7.6 Overdose ... 28
7.7 Treatment Supply and Labelling ... 28

7.7.1 Storage 28
7.8 Concomitant Medication 28
7.9 Assessments 29
7.9.1 Pre-treatment Screening Assessments 29
7.9.2 Assessments during Treatment 29
7.9.3 Off Study assessments 30
7.9.4 30 Day Post-Discontinuation assessments 31
7.9.5 Ad Hoc Follow-Up assessments 31
7.9.6 Post-Treatment Follow-Up 31
7.9.7 Phase II: Assessments Summary Table 32
7.9.8 Patient Safety Monitoring 34
7.10 Evaluation Criteria 34
7.10.1 Efficacy 34
7.10.2 Measurability of Tumour Lesions at Baseline 34
7.10.3 Tumour Response Evaluation 34
7.10.4 Frequency of Tumour Re-evaluation 34
7.10.5 Date of Progression 34
7.10.6 Reporting of Tumour Response 34
7.10.7 Duration of Tumour Response 35
7.10.8 Progression Free Survival 35
7.11 Patient Follow Up 35
7.12 Patient Withdrawal 35
8 Translational Research 35
8.1 Objectives 35
8.2 Tissue Samples 36
8.3 Blood samples 36
8.3.1 Angiogenic growth factors 36
8.3.2 Pharmacokinetics 37
8.3.3 PBMC Sub-study 37
9 Adverse Event Reporting 38
9.1 Reporting Requirements 38
9.1.1 Adverse Events 38
9.1.2 Serious Adverse Events 38
9.1.3 Reporting period 38
9.2 Reporting Procedure 38
9.2.1 Site 38
9.2.2 Trial Office 39
9.2.3 Reporting to the Competent Authority and main Research Ethics Committee 39
9.2.4 Manufacturer of Investigational Medicinal Product (IMP) 40
10 Data Handling and Record Keeping 40
10.1 Data Collection 40
10.2 Archiving 42
11 Quality Management 42
11.1 Site Set-up and Initiation 42
11.2 On-site Monitoring 42
11.3 Central Monitoring 42
11.4 Audit and Inspection 43
11.5 Notification of Serious Breaches 43

12 End of Trial Definition ... 43
13 Statistical Considerations ... 44
13.1 Statistical Design ... 44
13.1.1 Phase I ... 44
13.1.2 Phase II ... 44
13.2 Statistical Analysis ... 44
13.2.1 Phase I ... 44
13.2.2 Phase II ... 45
14 Trial Organisational Structure ... 45
14.1 Sponsor ... 45
14.2 Coordinating Centre ... 45
14.3 Trial Management Group (TMG) ... 45
14.4 Safety Review Committee ... 45
14.5 Data Monitoring Committee ... 45
14.6 Finance ... 46
15 Ethical Considerations ... 46
16 Confidentiality and Data Protection ... 46
17 Insurance and Indemnity ... 47
18 Publication Policy ... 47
19 Reference List ... 47
20 Appendices ... 48
20.1 Appendix 1 - AIDS Clinical Trials Group Oncology Committee Documentation of Disease and Definitions of Response ... 48
20.2 Appendix 2 - WMA Declaration of Helsinki ... 52
20.3 Appendix 3 - Definition of Adverse Events ... 55
20.4 Appendix 4 - Common Toxicity Criteria Gradings ... 56
20.5 Appendix 5 – New York Heart Association Classification – Stages of Heart Failure ... 56
20.6 Appendix 6 – Canadian Cardiovascular Society Grading of Angina Pectoris ... 56
20.7 Appendix 7 – Guidance for management of patients with a reduction in LVEF ... 57
20.8 Appendix 8 – Guidance for investigation of patients with new/worsening dyspnoea* (not considered related to disease under study) ... 58
20.9 Appendix 9 – Guidance for management of patients with visual symptoms ... 59
20.10 Appendix 10 – Guidance for management of creatine kinase elevation ... 60
20.11 Appendix 11 – Guidance for the management of patients with diarrhoea ... 61
20.12 Appendix 12 – Guidance for the management of patients with rash ... 62

1 BACKGROUND AND RATIONALE

1.1 HIV and KS

The prevalence of HIV in the UK is rising with about 83,000 living with HIV and 7,000 new cases per annum (pa). At diagnosis a third of patients have severe immune-suppression with a CD4 count less than 200/mm^3 (HPA 2009), which is associated with opportunistic infections and an increase in various tumours. Cancer is a leading cause of death in individuals living with HIV, and KS remains the commonest AIDS-associated malignancy. 5.5% of HIV positive patients developed KS in a UK prospective cohort followed in the HAART era (Stebbing *et al*. 2006).

KS is associated with co-infection with HIV and human herpesvirus-8 (HHV-8). Patients typically present with multi-focal cutaneous disease often with associated lymphoedema. Extra-cutaneous disease commonly involves the gastrointestinal tract, lung, liver and spleen. For early KS, initiation of HAART may be sufficient to control the disease and radiotherapy is of benefit for localised disease (Di Lorenzo, *et al*. 2007). Currently the only alternative for progressive localised disease is cytotoxic chemotherapy.

Cytotoxic chemotherapy with liposomal anthracycline or taxanes, is indicated in patients with widespread cutaneous KS, extensive oral disease or symptomatic visceral involvement (Bower *et al.* 2008). Pegylated liposomal doxorubicin (PLD) 20mg/m^2 q 3 weeks as first-line therapy in combination with HAART is reported to give tumour response in 55% of patients and median progression free survival (PFS) of 22 weeks (Cooley, *et al*. 2007). Second-line therapy with low dose paclitaxel (100mg/m2 q 2 weeks) is reported to give a response rate of 56% with median PFS of 39 weeks (Tulpule, *et al*. 2002). However the majority of patients progress despite chemotherapy and new treatment alternatives are required.

1.2 Mitogen-activated protein kinase (MAPK) Pathways and KS

MAPK pathways are signal transduction pathways utilised by many growth factor receptors including epidermal growth factor (EGF), insulin-like growth factor-1 (IGF) and platelet-derived growth factor (PDGF), which play essential roles in cell survival, proliferation and differentiation (Adjei *et al*. 2008). Genetic mutations and/or over expression of these growth factors, their receptors, downstream signalling proteins, or protein kinases involved in the Ras/Raf/MEK/ERK MAPK pathway have been implicated in a variety of tumours including lung cancer, melanoma, thyroid cancer and pancreatic tumours. The MAPK Extracellular signal-regulated kinase Kinase (MEK) is also stimulated by a variety of chemokines including interleukin 6 (IL-6), IL-1β, interferon-γ and vascular endothelial growth factor (VEGF) and is important in mediating immune responses. MEK has also been identified as important in viral infections including HHV-8 and human immunodeficiency virus type 1 (HIV-1) during the early infection phase and reactivation following viral latency (Yang and Gabuzda 1999; Sharma-Walia, *et al.* 2005; Xie, *et al*. 2008). In KS, the HHV-8 encoded proteins viral interleukin-6 (vIL-6) and viral G protein coupled receptor (vGPCR) upregulate the Ras/Raf/MEK/ERK pathway (Vart, *et al*. 2007). In KS this has been shown to stimulate expression of Angiopoeitin-2 (Ang-2), a pro-angiogenic factor (Vart, *et al.* 2007), which may further promote KS development. Plasma Ang-2 levels have been shown to correlate with volume of disease (Wang, *et al*. 2004). The MAPK pathway therefore provides an attractive therapeutic target in KS.

1.3 Selumetinib

Selumetinib (AZD6244 Hyd-Sulphate; 6-(4-Bromo-2-chloro-phenylamino)-7-fluro-3-methyl-3H-benzoimidazole-5-carboxylic acid (2-hydroxy-ethoxy)-amide hydrogen sulphate; ARRY-142886) is an orally bioavailable, selective inhibitor of MEK 1/2, inhibiting the phosphorylation of ERK 1/2 (Banerji, *et al.*). It is inactive, or minimally active against other kinases including p38 and JNK. N-desmethyl AZD6244, a pharmacologically active metabolite, is 3 to 5-fold more active than the parent compound. However, the amide metabolite is up to 50-fold less active, and is unlikely to significantly contribute to biological activity. AZD6244 is metabolised by cytochrome P450 (CYP) enzymes 1A2, 2C19 and 3A4.

CYP1A2 is primarily responsible for the formation of the N-desmethyl metabolite. AZD6244 is a weak inducer of CYPs 3A, 1A and 2C9. AZD6244 does not inhibit CYPs 1A2, 2C8, 2C19, 2D6 and 3A4. Direct conjugation and biliary excretion is the primary route for elimination of AZD6244.

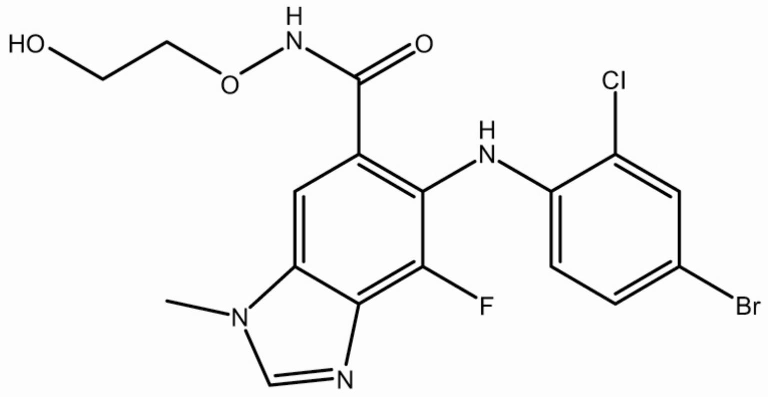

AZD6244 is available in oral suspension and the AZD6244 Hyd-Sulphate salt (selumetinib) was developed in order to provide the drug in capsule form. A phase I trial of selumetinib monotherapy identified 75 mg bd as both safe and tolerable (Banerji, *et al.*). AZD6244 exposure was significantly higher with the 75 mg capsule relative to 100 mg free-base suspension; C_{max} and AUC_{0-24} were estimated to be 252% (90% CI 182-348%) and 197% (90% CI 161-242%) respectively. The exposure of the active metabolite N-desmethyl AZD6244 was reported as similar for both formulations as was clearance (CL/F) and $t_{1/2}$ values were similar. The adverse event profile observed in this phase I study was broadly consistent with that previously seen with AZD6244. The most frequently reported adverse events were dermatitis acneiform, diarrhoea, nausea, vomiting, peripheral oedema, blurred vision and fatigue. The majority of these events were Common Terminology Criteria for Adverse Events (CTCAE) grade 1 or 2. There was a trend for increased systolic and diastolic blood pressure which had resolved by week 12 of the study and a trend for decrease in left ventricular ejection fraction (LVEF) with no obvious dose dependency. The mean decrease at week 8 was -7.2% (range -25% to +10%). Small increases in alanine transaminase (ALT), aspartate aminotransferase (AST) and alkaline phosphatase (ALP) were observed within 1 week of initiation of therapy but did not rise beyond 28 days of dosing. A phase II randomised open-label study comparing selumetinib with capecitabine for advanced pancreatic cancer has recently reported (Bodoky, *et al.*). 37 patients received selumetinib; toxicity and overall survival were similar to the capecitabine arm. Selumetinib is under investigation in phase II monotherapy studies in comparison with temozolomide in patients with unresectable American Joint Committee on Cancer (AJCC) stage 3 or 4 malignant melanoma (NCT00338130). Selumetinib is under investigation in phase I combination studies with docetaxel, dacarbazine, erlotinib or temsirolimus in patients with advanced solid tumours (NCT00600496), and in two phase II double-blind, placebo controlled studies - dacarbazine 1000mg/m^2 iv 3 weekly +/- selumetinib 75mg bd in patients with BRAF mutation positive advanced melanoma (NCT00936221), and docetaxel 75mg/m^2 iv 3 weekly +/- selumetinib 75mg bd in patients with KRAS mutation positive stage IIIB/IV non-small cell lung cancer (NCT00890825). Preliminary data from the combination studies indicate adverse event profiles are consistent with individual monotherapy profiles. Full details are available in the Investigator Brochure.

1.4 Highly Active Anti-Retroviral Therapy (HAART)

The commonest HAART regimen currently used in the UK is Atripla (Gilead Sciences Ltd), a combination of Efavirenz, Emtricitabine and Tenofovir. This is a once daily tablet taken on an empty stomach. Common side effects include dizziness, headache, insomnia, drowsiness, rash, fatigue, vomiting and diarrhoea. Efavirenz (a non-nucleoside reverse transcriptase inhibitor, NNRTI) is metabolised by CYP3A and CYP2B6 and excreted in the urine or excreted unchanged in faeces. It is

known to induce CYP3A. Emtricitabine (a nucleoside reverse transcriptase inhibitor, NRTI) is principally excreted unchanged in urine and has no apparent interaction with CYP450. Tenofovir (a nucleotide reverse transcriptase inhibitor, NtRTI) is not a substrate for CYP450 and is excreted unchanged in the urine. At concentrations 300-fold higher than found in vivo a small but significant inhibition of substrates for CYP1A metabolism was observed. No other interactions with CYPs have been reported.

The protease inhibitors are another important class of anti-retroviral agents. They may be associated with CYP3A inhibition particularly Ritonavir (Abbott Laboratories). Inhibition of CYP1A (responsible for metabolizing AZD6244 to the more active N-desmethyl metabolite) is not reported.

1.5 Trial Rationale

1.5.1 Justification for patient population

HAART remains fundamental to the treatment of AIDS-associated KS. Treatment alternatives to cytotoxic chemotherapy are required for patients on HAART with **locally** progressive KS in whom chemotherapy is not indicated, and patients with **advanced** KS who have progressed despite chemotherapy. Eligibility criteria for this study therefore include patients with AIDS-associated KS established on HAART specifically in order to investigate the effect of selumetinib on HAART pharmacokinetics.

1.5.2 Justification for design

Selumetinib has been tested in a number of phase I and phase II trials as both monotherapy and combined with cytotoxic chemotherapy in patients with advanced solid malignancies. A toxicity profile and recommended dose has been established in these patients. Selumetinib has not been tested in combination with HAART. No significant interactions are predicted between selumetinib and HAART however a phase I study is required to investigate the pharmacokinetic effects of combining these drugs. In particular we wish to establish that selumetinib will not reduce the efficacy of HAART. An accelerated phase I trial is proposed commencing at 1 dose level below that recommended for monotherapy or in combination with cytotoxic chemotherapy. Evidence of efficacy will be collected by objective response rate in an open label single arm phase II study. Evidence of efficacy will be used to develop a protocol for a future randomised phase II/III study.

1.5.3 Choice of treatment

There is a strong rationale for targeting the MAPK pathway in KS, as described in Section 1.2 selumetinib is an orally bioavailable, selective inhibitor of MEK 1/2. Its safety profile, recommended dose and evidence of efficacy have been established in phase I and phase II trials both as monotherapy and combined with cytotoxic chemotherapy in patients with advanced solid malignancies. This study will determine safety and the recommended dose of selumetinib in combination with HAART and assess the response rate in patients with AIDS-associated KS.

2 AIMS, OBJECTIVES AND OUTCOME MEASURES

2.1 Aims and Objectives

2.1.1 Primary Objectives

- To identify a safe dose for selumetinib in combination with HAART.
- To establish evidence of the efficacy of selumetinib in combination with HAART in patients with AIDS-associated KS.

2.1.2 Secondary Objectives

- To describe the pharmacokinetics of selumetinib in combination with HAART, including the effects of selumetinib on HAART metabolism.
- To assess the toxicity of selumetinib in combination with HAART.
- To describe the pharmacodynamic effects of selumetinib in combination with HAART.
- Progression free survival rate at 6 months from commencing treatment.

2.2 Outcome Measures

2.2.1 Phase I

- Selumetinib and metabolite serum levels pre-dose and at 2 and 6 hours on day 1 and pre-dose on day 15.
- HAART drug levels pre-dose on days 1, 15 and at 12 weeks.

2.2.2 Phase I & II

- Toxicity assessed by CTCAE v 4.0 criteria (see Appendix 4).
- Number of selumetinib cycles completed and PFS data will be collected as supportive data out to 12 months from the last patient completing study treatment.
- Objective response rates assessed using ACTG criteria (see Appendix 1).
- Progression free survival rate 6 months from the start of study treatment assessed using ACTG criteria.
- Human immunodeficiency virus (HIV) control by HIV-1 viral load pre-treatment and weeks 3, 6, 12 and 18, and CD4 counts pre-treatment and at weeks 9 and 18.
- Pharmacodynamic measures of selumetinib in combination with HAART assessed by:
    - Serum concentrations of angiogenesis markers including serum Ang-2, IL-6 and VEGF assessed by ELISA pre-study and on weeks 3, 6, 12 and 18.
    - Biopsies pre-study and at week 6 to assess pERK levels in tumour tissue and other downstream markers including c-myc, c-fos, changes in apoptosis markers such as Bad and Bcl-2, and adaptive changes in PI3K/Akt and other MAPK pathways such as JNK and p38.
- Analysis of peripheral blood mononuclear cells (PBMCs) collected in a sub-study at selected centres pre-treatment and at weeks 9 and 18.
    - Levels of pERK, down-stream targets c-fos and c-myc, and key apoptotic proteins Bad and Bcl-2 will be assessed by Western blotting of lysed viable PBMCs.
    - PBMCs will be challenged with TLR4 and 9 agonists and production of IL-1β, TNF-α, IL-6, IL-10, IL-12 and type 1 interferon will be measured by cytometric bead array of culture supernatants.
    - ROS production by PBMCs will be measured by flow cytometry using dichlorofluorescein (DCF) and cell survival assessed by Annexin V-PE/TO-PRO3 staining and flow cytometry.

3 TRIAL DESIGN

This is an open-label multi-centre phase I/II study. In phase I, patients will be recruited to establish the dosage, safety, pharmacokinetics and pharmacodynamics of selumetinib in combination with HAART in KS using a 3 + 3 dose escalation algorithm. In phase II, efficacy will be assessed by best objective tumour response in a single patient cohort according to a two stage Simon design. Potential patients will be identified by the treating oncologist or referring communicable disease team (i.e. Infectious Disease or Genito-Urinary Medicine).

3.1 Phase I

75 mg bd is the recommended dose for phase II monotherapy studies. Interactions between selumetinib and HAART are not anticipated. Therefore for this study the starting dose of selumetinib is 50 mg PO bd in combination with HAART. The dose of selumetinib will be escalated in fixed increments thus:

Dose level	Dose of Selumetinib PO, (total daily dose)	Minimum number of patients
-1	75 mg od (75 total)	--
1 (starting)	50 mg bd (100 total)	3
2	75 mg bd (150 total)	3
3	100 mg bd (200 total)	3

Three patients will be enrolled at the starting dose of 50mg bd. All three will be followed for at least one completed cycle (21 days) and a Safety Review Committee teleconference to discuss the dose escalation will occur before opening new cohorts. Subsequent enrolment will be based on observed Dose Limiting Toxicities (DLT):

- If 0/3 patients exhibit DLT, dose escalate to level 2 in a new cohort of patients
- If 1/3 patients exhibit DLT at the starting dose, expand the cohort to a total of 6 patients
  - If no further DLT events are seen, dose escalates to level 2 in a new cohort of patients.
  - If 1 or more further DLT events are seen (i.e. 2 or more from 6 patients) the dose of selumetinib will be de-escalated and 3 patients recruited to a new cohort at the -1 dose level.
- If 2/3 patients exhibit dose limiting toxicity at the starting dose level
  - The dose will be de-escalated to dose level -1, and 3 patients recruited to the lower dose.
  - If 1/3 patients exhibit DLT at the new lower dose level this cohort will expand to a total of 6 patients.
- The dose will be escalated to dose level 3 only if no patient exhibits a DLT at dose level 1 or 2 and pharmacokinetic data show inadequate selumetinib serum levels at lower dose levels.

Patients recruited to 50mg bd who experience no toxicity in cycle 1 can be dose escalated to 75mg bd selumetinib in subsequent cycles.

Patients who continue to respond to selumetinib following completion of 6 cycles of therapy will be offered the option of continuing selumetinib. In this instance a clinical investigator at sites should email the SCART Trial Office, outlining the clinical benefit experienced by the patient when the patient has reached the C5D22/C6D1 visit. This will be forwarded to AstraZeneca for approval. The approval will then be communicated to sites. Patients who have been shown to derive benefit from the selumetinib and HAART combined therapy at one year should be continued on this regimen as long as their risk: benefit analysis, in the opinion of the treating physician, favours the continuation of treatment and with approval from AstraZeneca. In this instance sites should contact the SCART Trial Office.

3.1.1 Dose Limiting Toxicity (DLT)

For determination of the Maximum Tolerated Dose (MTD), DLTs will be assessed during Cycle 1 in Phase I only, (i.e. up to the time of Cycle 2 day 1 assessment). Toxicity will be graded using CTCAE version 4.0 (Appendix 4). Any DLT must be considered at least possibly related to selumetinib. DLT is defined as follows:

3.1.1.1 Haematologic

- Absolute neutrophil count (ANC) < 1.0 x 10^9/L
- Febrile neutropenia (ANC < 1.0 x 10^9/L, fever ≥ 38°C)
- Platelets < 50 x 10^9/L
- Bleeding thought to be due to thrombocytopenia

3.1.1.2 Non-Haematologic

- Diarrhoea ≥ Grade 3 despite optimal loperamide use
- Rash ≥ Grade 3
- Any grade 2 toxicity unacceptable to the patient that requires a dose reduction
- Missing >30% of treatment doses for toxicity reasons
- **Any other grade 3 or 4 effects thought to be treatment related**

3.1.1.3 HIV control

- The study subjects' HAART PK drug levels, HIV viral load and CD4 counts will be made available to their principal investigator and HIV physician immediately following analysis. Detrimental changes in HIV control considered at least possibly related to the study drug must be discussed with the trial management team HIV physician (Prof Dockrell) and chief investigator (Dr Young). Persistent increases in HIV viral load above 200 copies/ml and/or clinically significant decreases in CD4 count will result in the subject discontinuing the study drug.
- As an early phase clinical trial with specific pharmacokinetic and pharmacodynamic endpoints, it is important that patients' HAART regimen is not be changed for their first 3 months on the trial (i.e. until after the C4D22/C5D1 visit) unless clinically necessary.

3.1.2 Maximum Tolerated Dose (MTD)

The MTD is defined as the highest dose producing one or less DLT in 6 patients. This dose will be used as the recommended phase II dose (RPTD). Phase I is now complete and the recommended phase II was determined to be 75 mg bd.

3.2 Phase II

Patients will be recruited in a single-arm study at the RPTD, in order to determine objective tumour response using AIDS Clinical Trials Group Oncology Committee (ACTG) criteria (see Appendix 1). Patients recruited to the RPTD during phase I will be included in the phase II study. KS will be evaluated using clinical photographs of target lesions and bi-dimensional measurements every 3 weeks and/or CT imaging of assessable disease sites every 6 weeks during treatment. As in phase I, patients who continue to respond to selumetinib following completion of 6 cycles of therapy will be offered the option of continuing selumetinib. Patients who have been shown to derive benefit from selumetinib and HAART combined therapy at one year should be continued on this regimen as long as there has been no disease progression and their risk: benefit analysis, in the opinion of the Chief Investigator and treating physician, favours the continuation of treatment and with approval from AstraZeneca. To continue treatment beyond 6 cycles sites should contact the SCART Trial Office as described in section 3.1. Patients' HAART regimen should not be changed for their first 3 months on the trial (i.e. until after the C4D22/C5D1 visit) unless clinically necessary. If deemed clinically necessary, sites should contact the SCART Trial office to facilitate discussion with the Chief Investigator and Clinical Co-Investigators.

4 ELIGIBILITY

4.1 Inclusion Criteria

- HIV positive and established on a HAART regimen for ≥ 3 months.
- Histologically confirmed KS
- Measurable disease according to ACTG criteria.
- Progressive cutaneous or nodal KS not requiring chemotherapy **OR** progressive KS following cytotoxic chemotherapy.
- Evidence of disease progression in the past 6 months. No anti-cancer treatment within one month prior to commencing trial treatment.
- Adequate haematological function:
  - Haemoglobin ≥ 9 g/dL
  - Absolute neutrophil count ≥ 1.5 x 10^9/L
  - Platelets ≥ 100 x 10^9/L
- Adequate hepatic function:
  - Serum bilirubin ≤ 1.5 x upper limit of normal (ULN), except if the patient is established on the anti-retroviral drug atazanavir (no upper limit) and has AST and ALT levels ≤ 2.5 x ULN
  - ALT ≤ 2.5 x ULN
  - AST ≤ 2.5 x ULN
- Adequate renal function:
  - Serum creatinine clearance > 50 ml/min (Cockcroft-Gault formula or 24 hour urine collection).
- Age ≥ 18 years.
- World Health Organisation (WHO) performance status ≤ 2.
- For selumetinib, women of child bearing age and child bearing potential **MUST** have a negative pregnancy test prior to study entry **AND** be using an adequate contraception method, which must be continued while on treatment and for at least 4 weeks after the study treatment has ended.
- Male patients must agree to use an effective contraception method while on treatment and for at least 16 weeks after the study treatment has ended (barrier contraception is recommended for all individuals living with HIV).
- Written informed consent.

4.2 Exclusion Criteria

- HIV viral load > 200 copies/ml.
- Active opportunistic infections.
- Active hepatitis B, hepatitis C.
- Any prior exposure to MEK, Ras, or Raf inhibitors or history of hypersensitivity to selumetinib, or any excipient agents.
- Any unresolved toxicity > CTCAE Grade 2 from previous anti-cancer therapy, except for alopecia
- Cardiac conditions as follows:
  - Uncontrolled hypertension (BP ≥150/95 mmHg despite medical therapy)
  - Left ventricular ejection fraction <55% measured by echocardiography
  - Atrial fibrillation with a ventricular rate >100 bpm on ECG at rest
  - Symptomatic heart failure (NYHA grade II-IV)
  - Prior or current cardiomyopathy
  - Severe valvular heart disease
  - Uncontrolled angina (Canadian Cardiovascular Society grade II-IV despite medical therapy)
  - Acute coronary syndrome within 6 months prior to starting treatment
- Major surgery within 4 weeks prior to starting selumetinib.
- Refractory nausea, vomiting, chronic gastrointestinal diseases (e.g. inflammatory bowel disease) or significant bowel resection that would preclude adequate absorption.

- Ophthalmological conditions as follows:
    - Intra-ocular pressure >21 mmHg, or uncontrolled glaucoma (irrespective of intra-ocular pressure)
    - Current or past history of central serous retinopathy or retinal vein occlusion
- Evidence of any psychological, familial, sociological or geographical condition potentially hampering protocol compliance.
- Treatment with any investigational product within 28 days of registration.
- Clinical judgement by the Investigator that the patient should not participate in the study.
- Pregnant or breast-feeding women.

4.2.1 Asian Patients

Due to higher PK exposure, patients of Asian ethnicity may be at a higher risk of adverse events with selumetinib treatment. Selumetinib is not contra-indicated in patients of Asian ethnicity, but the potential increased risk of toxicity should be considered and included as part of the discussion with the patient prior to receiving informed consent (see section 7.3.1.1 for further details).

5 PATIENT SCREENING AND CONSENT

5.1 Screening

Potentially eligible HIV positive patients with confirmed progressive KS on an established HAART regimen (≥ 3 months) can be screened for entry into this trial. For patients who appear to meet the criteria for participation in the trial, the Investigator will provide information to allow them to make an informed decision regarding their participation. If informed consent is given, the Investigator will conduct a full screening evaluation to ensure that the patient satisfies all inclusion and exclusion criteria. A patient who gives written informed consent and who satisfies all the inclusion and exclusion criteria may be entered into the trial. Patients should only be approached about the trial if there is a slot available. Investigators must keep a screening log of all patients who receive a patient information sheet.

5.1.1 Patient Screening Log

The following details of all patients screened for participation in this study will be collected:

- Date screened
- Date of birth
- Gender
- Whether the patient was eligible and reasons for ineligibility
- Whether the patient gave written informed consent
- Whether the patient was registered
- Reason(s) patient was not registered

5.2 Informed Consent

It is the responsibility of the Investigator to obtain written informed consent for each patient prior to performing any trial related procedure. A Patient Information Sheet (PIS) is provided to facilitate this process. Investigators must ensure that they adequately explain the aim, trial treatment, anticipated benefits and potential hazards of taking part in the trial to the patient. The Investigator should also stress that the patient is completely free to refuse to take part or withdraw from the trial at any time. The patient should be given ample time (at least 24 hours) to read the PIS and to discuss their participation with others outside of the site research team. The patient must be given an opportunity to ask questions which should be answered to their satisfaction. The right of the patient to refuse to participate in the trial without giving a reason must be respected.

If the patient wishes to participate in the trial they should be asked to sign and date the latest version of the Informed Consent Form (ICF) in the presence of the Investigator or designee who must then co-sign

and date the form. The patient must personally initial all the boxes. A copy of the ICF should be given to the patient, a copy should be filed in the hospital notes, and the original placed in the Investigator Site File (ISF). Once the patient is entered into the trial the patient's registration number should be entered on the ICF maintained in the ISF.

Details of the informed consent discussions should be recorded in the patient's medical notes, this should include date of, and information regarding, the initial discussion, the date consent was given, with the name of the trial and the version number of the PIS and ICF. Throughout the trial, the patient should have the opportunity to ask questions about the trial and any new information that may be relevant to the patient's continued participation should be shared with them in a timely manner. On occasion it may be necessary to re-consent the patient in which case the process above should be followed and the patient's right to withdraw from the trial respected.

Electronic copies of the PIS and ICF are available from the Trial Office and should be printed or photocopied onto the headed paper of the local institution. Details of all patients approached about the trial should be recorded on the Patient Screening/Enrolment Log and with the patient's prior consent the physician co-ordinating their medical care (i.e. their General Practitioner (GP) and/or HIV Physician) should also be informed that they are taking part in the trial. If the patient does not consent to their GP being informed, then their HIV clinician should be informed instead. A GP/HIV clinician Letter is provided electronically for this purpose.

6 TRIAL ENTRY

Prior to recruitment of patients into the study, the Principal Investigator for each site, or their designee, should have returned all required documentation to the SCART Trial Office, and the site personnel involved with the SCART study must have received appropriate training from the Trial Coordinator.
Patients must be registered with the CRCTU at the University of Birmingham. Before approaching a potential patient, Investigators should check that there is a slot available with the SCART Trial Office. The patient's eligibility will be confirmed at registration. If eligible for the study, the patient will be allocated a Registration Number and dose. Study drug prescriptions must include the patient's Registration Number and allocated dose level. The registration line will be open during office hours (9am-5pm, Monday-Friday).

Registration Line: 0121 414 6788 or 0121 414 6754

7 TREATMENT DETAILS

7.1 Selumetinib

The Investigational Medicinal Product in this trial is selumetinib. The starting dose of selumetinib for each patient will be released with their Registration Number. Selumetinib should be taken twice daily approximately 12 hours apart with water, at least 2 hours after a meal and 1 hour before the next meal. Selumetinib capsules will be administered in doses as described in section 3 in a continuous 21 day cycle, unless disease progression (see section 7.4) or unacceptable toxicity is encountered. The trial design, dose levels and numbers of required patients are shown in section 3.1.

7.2 Treatment Schedule

Six cycles, each of 21 days, will be given unless disease progression or unacceptable toxicity is encountered. Patients who are responding after six cycles of selumetinib will be offered the option of continuing on selumetinib in combination with HAART. Patients who have had no disease progression and have been shown to derive benefit from the selumetinib and HAART combined therapy at one year should be continued on this regimen in an extension of the study as long as their risk: benefit analysis, in the opinion of the Chief Investigator and treating physician, favours the continuation of treatment and with approval from AstraZeneca. However, if after one year there is disease progression, treatment

should be discontinued and the patient should come off study. It should be noted that there is no compassionate use or access to selumetinib once off study.
Patients may also discontinue protocol therapy in the following instances:

- Intercurrent illness which would in the judgment of the investigator affect patient safety, the ability to deliver treatment or the primary study endpoints
- Request by patient

7.3 Expected Toxicity

7.3.1 Adverse Events Associated With Selumetinib

The following adverse events have been associated with administration of selumetinib and are regarded as expected for regulatory reporting purposes. These are listed to help define SAEs and SUSARs, but must still be reported as AEs.

- Eye disorders: vision blurred.
- Gastrointestinal disorders: diarrhoea, nausea and vomiting.
- Respiratory disorders: dyspnoea
- Skin and subcutaneous tissue disorders: dry skin and rash, including dermatitis acneiform and exfoliative rash.
- Vascular disorders: hypertension.
- General disorders: facial and/or peripheral oedema, fatigue, mucositis.
- Metabolic disorders: creatine phosphokinase elevation, hypoalbuminaemia, hyperphosphataemia, which may be associated with an increase in calcium:phosphate product requiring therapeutic intervention.
- Investigations: increases in serum AST and ALT.

In ongoing studies of selumetinib (Hyd-AZD6244) and the free-base suspension (AZD6244), the most commonly reported adverse events (all grades) among 300 patients were:

Incidence > 30%	Incidence 10-20%
Rash (acneiform or maculo-papular)<br>Fatigue<br>Diarrhoea<br>Nausea<br>Vomiting	Oedema (peripheral and facial)<br>Hypertension (increased SBP and DBP)<br>Anorexia<br>Dry mouth<br>Oral mucositis<br>Dry skin<br>Dyspnoea<br>Abdominal pain<br>Constipation<br>Aspartate aminotransferase increased<br>Alanine aminotransferase increased<br>Alkaline phosphatase increased<br>Hypoalbuminaemia<br>Anaemia<br>Platelet count decreased<br>Hypomagnesemia

- The most commonly reported treatment-related adverse events of at least grade 3 were rash (9%), fatigue (7%), diarrhoea (5%), vomiting and nausea (4%) and transaminitis (≤4%).Asymptomatic decreases in left ventricular ejection fraction (LVEF) have been observed in patients receiving AZD6244. LVEF assessments on study are defined in Section 7.9, and an investigational algorithm shown in Appendix 7.

- A small number of patients with advanced cancer have reported pneumonitis adverse events during AZD6244 treatment, and interstitial lung disease-type events have been reported in patients with advanced NSCLC receiving selumetinib in combination with docetaxel. The main symptoms include shortness of breath, fatigue and fever. An association with AZD6244 treatment has not been established however AZD6244 cannot be definitively excluded as a cause of these events. All new dyspnoea AEs or worsening of pre-existing dyspnoea AEs should be followed according to the dyspnoea algorithm Appendix 8.

- Blurred vision, generally CTCAE Grade 1, has been reported in some patients enrolled in studies of selumetinib monotherapy or in combination with other anti-cancer agents. AEs consistent with central serous retinopathy have been reported in a small number of patients receiving treatment with selumetinib, generally in combination with other anti-cancer agents targeting AKT inhibition or IGFR-IR inhibition, or with cytotoxic chemotherapies. Rare AEs of retinal vein occlusion have been reported, although a relationship between selumetinib and retinal vein occlusion has not been established. Guidance for the management of changes in visual function is shown in Appendix 9. Retinal changes should be followed up to resolution to evaluate the potential for reversibility.

- A recent study of selumetinib (75mg bd) in combination with docetaxol (75mg/m^2 iv day 1, every 21 days) for non-small cell lung cancer reported febrile neutropenia in 8/44 patients on the combination arm compared to 0/42 in the chemotherapy alone arm. 6/44 had febrile neutropenia CTCAE grade 3, 2/44 CTCAE grade 4. No patients died from febrile neutropenia.

- Creatine phosphokinase (creatine kinase) elevation has been reported with some MEK inhibitors. Creatine phosphokinase is released from a number of tissue types, including skin and muscle; the origin of elevations reported in patients receiving MEK inhibitors is unknown. A causal relationship between selumetinib and creatine phosphokinase elevation has not been established. Guidance for the management of elevated creatine phosphokinase is shown in Appendix 10.

- Reproductive toxicology data indicate that selumetinib has adverse effects on embryofoetal development and survival at dose levels that do not induce maternal toxicity in mice. Subsequently, selumetinib should not be administered to pregnant or breast-feeding women and conception while on treatment must be avoided. Female patients of child-bearing potential will be required to use reliable methods of contraception for the duration of the study and until 4 weeks after the last dose of selumetinib. Male patients with sexual partners who are pregnant or who could become pregnant (i.e. women of child-bearing potential) should use acceptable methods of contraception for 16 weeks after completing the study to avoid pregnancy and/or potential adverse effects on the developing embryo. In any case barrier contraception is recommended for individuals living with HIV.

7.3.1.1 Asian Pharmacokinetic Data Associated with Selumetinib

The pharmacokinetics of selumetinib were investigated in study D1532C00086, conducted in the UK involving healthy volunteers of Asian ethnicity (defined as being born in an Asian country, and expatriate for not longer than 5 years, and with maternal and paternal grandparents of Asian ethnicity). The subjects who received selumetinib in study D1532C00086 were of the following ethnicities: Japanese, Chinese, Filipino, Malay, Malaysian, Maldivian, Singaporean, Thai, Indian and Vietnamese, and it is not known in these groups whether selumetinib exposure will be similar to Western subjects or to subjects of the specific Asian ethnicities included in Study D1532C00086.

The pharmacokinetic findings from study D1532C00086 do not support excluding subjects of Asian ethnicity from studies of selumetinib. However, as it is possible that Asian subjects may experience higher selumetinib plasma exposure (than would be expected in Western subjects receiving the same dose of selumetinib), there could be a potential for a higher risk of adverse events.

The number of Asian patients with advanced cancer who have received treatment with selumetinib is very low. Emerging information from ongoing study D1532C00067 of Japanese patients receiving selumetinib + Docetaxel for second-line treatment of NSCLC suggests that febrile neutropenia may occur more commonly in Japanese patients (3 of 8 patients treated, although comparative data in Japanese patients receiving Docetaxel monotherapy is not available) than might be predicted from studies conducted in Western subjects.

Patients of Asian ethnicity are not excluded from studies evaluating selumetinib. However, when considering enrolling an individual of Asian ethnicity to a selumetinib clinical study, investigators should make a clinical judgment as to whether the potential risk of experiencing higher selumetinib plasma levels outweighs the potential benefit of treatment with selumetinib. The Patient Information Sheet for studies of selumetinib includes information on the possibility of higher selumetinib plasma levels and occurrence of adverse events in Asian subjects than in subjects who are not of Asian origin. Investigators should be aware of the potentially higher risk of adverse events when monitoring patients of Asian ethnicity receiving treatment in clinical studies of selumetinib.

7.3.2 Adverse Events Associated With HAART

Adverse effects of HAART vary with specific regimens. Details are available at: http://hivinsite.ucsf.edu/InSite?page=ar-05-01

7.3.3 Management of nausea and vomiting

Nausea and vomiting should be graded using CTCAE criteria. Selumetinib dose modifications are defined in section 7.4. Symptoms should be managed according to local treatment protocols.

Nausea and vomiting should be actively managed including regular anti-emetics if required in order to maintain HAART compliance. If compliance with HAART medication is at risk due to uncontrolled nausea or vomiting the patient should be reviewed and alternative anti-emetics tried. If compliance or absorption of anti-retrovirals remains at risk despite this selumetinib should be discontinued.

If a patient does vomit and HAART medication is clearly visible in the vomitus, anti-emetics should be taken and HAART dosing repeated. Repeat dosing to supplement vomited selumetinib capsules is not advised.

7.3.4 Management of diarrhoea

Diarrhoea should be graded using CTCAE criteria. Selumetinib dose modifications are defined in section 7.4. Early initiation of treatment for diarrhoea is strongly recommended to minimise the duration and severity of the adverse event. A suggested algorithm for the management of diarrhoea is shown in Appendix 11.

7.5 Management of hypertension

Hypertension should be graded using CTCAE criteria. Selumetinib dose modifications are defined in section 7.4. Hypertension should be managed according to local treatment protocols.

7.3.5 Management of skin toxicities associated with Selumetinib

Skin toxicities should be graded using CTCAE criteria. Selumetinib dose modifications are defined in section 7.4. Early initiation of treatment for rashes is strongly recommended to minimise the duration and severity of the adverse event. A suggested algorithm for the management of skin rashes is shown in Appendix 12.

7.4 Dose Modifications

Selumetinib doses will be adjusted for haematological and other adverse events considered at least partly due to administration of selumetinib in combination with HAART. Dose adjustments are to be made according to the greatest degree of toxicity. Adverse events will be graded using the CTCAE (see Appendix 4). Dose escalation outside of the dose finding study in phase I is not permitted.

The guidelines which follow outline dose adjustments for several of these toxic effects. **If a patient experiences several adverse events and there are conflicting recommendations, please use the recommended dose adjustment that reduces the dose to the lowest level.**

Treatment with selumetinib should be withheld if one of the following toxicities considered related to treatment with selumetinib are observed, despite optimal supportive care:

- Any intolerable adverse event regardless of grade
- Any adverse event CTCAE grade ≥ 3 (should be reported as a DLT, see section 3.1.1 for definition).

In the event of a DLT, report to the SCART Trial office within 24 hours of the investigator becoming aware of the event, via phone or email **0121 414 6788 or SCART@contacts@bham.ac.uk**

7.4.1 Haematologic Adverse Events

**Grade of Event**	Management/Next Dose for Selumetinib
grade 1	No change in dose
grade 2	No change in dose
grade 3	Hold* until < grade 2 – resume at 1 dose level lower if indicated** (unless defined as a DLT).
grade 4	Off protocol therapy
* Patients requiring a delay of > 2 weeks should go off protocol therapy.<br>** Patients requiring > 2 dose reductions should go off protocol therapy.	

7.4.2 Non-Haematologic Adverse Events (including biochemistry/hepatic/renal function)

**Grade of Event**	Management/Next Dose for Selumetinib
grade 1	no change in dose
grade 2	Hold until ≤ grade 1∞ – resume at same dose, except for rash where CTCAE grade 2 rash is acceptable
grade 3	Hold* until < grade 2 – resume at 1 dose level lower if indicated,** except for rash where patients with CTCAE grade 2 rash may restart treatment (unless defined as a DLT).
grade 4	Off protocol therapy
* Patients requiring a delay of > 2 weeks should go off protocol therapy.<br>** Patients requiring > 2 dose reductions should go off protocol therapy.<br>∞Please refer to Appendix 10 for management of elevated creatine kinase	

The dose modification algorithm allows for the following dose reductions:

75mg bd → 50mg bd → 75mg od

7.5 [illegible]

Treat[illegible]es at the [illegible]ug returns will be disposed of by Pharmacy according to local hospital practice. Further information can be found in the Pharmacy Manual (supplied separately).

7.6 Overdose

There is currently no known antidote to selumetinib. Adverse reactions associated with overdose should be supportive for the underlying symptoms. Doses of study treatment in excess of that specified in the clinical study protocol are considered to be an overdose. Should an overdose (accidental or deliberate) occur, all symptoms associated with the overdose should be reported as AEs.

7.7 Treatment Supply and Labelling

The IMP will be supplied as capsules each containing 25mg of selumetinib, free of charge. Patients enrolled in the study will be dispensed bottles of selumetinib containing sufficient medication for dosing up to their next visit. No placebo will be used. When required, supplies can be ordered by completing the SCART Drug Request Form found in the Pharmacy File. Please note that drug will be supplied within 5 working days of ordering. All information regarding selumetinib supply can be found in the Pharmacy Manual (supplied separately).

7.7.1 Storage

Selumetinib capsules should be stored below 30°C. A full description of the appropriate storage conditions are specified on the investigational labels. Further details regarding treatment supply, storage and labelling can be found in the Pharmacy Manual (supplied separately).

7.8 Concomitant Medication

Patients are required to have been established on a HAART regimen for at least 3 months prior to study entry. Their HAART regimen should not be changed while they are on study, except after discussion with the CIs. Throughout the study, patients should avoid changes to, or the addition of all concomitant medications. A wide range of drug interactions can occur with HAART. Any proposed additional medications should be checked for potential interactions using the Liverpool HIV Drug Interaction charts: http://www.hiv-druginteractions.org/Interactions.aspx. In addition, drugs that might affect the metabolism of selumetinib (CYP1A2 or 3A4 inhibitors/inducers) should be avoided: http://medicine.iupui.edu/clinpharm/ddis/table.aspx. Pre-clinical studies predict minimal significant metabolism by CYP3A4, and that, as per protocol, HAART levels are monitored in phase I, and HIV viral loads and CD4 counts in both phase I & II. No other anti-cancer treatment (including radiotherapy) or investigational therapy is permitted whilst patients are on study therapy.

Increased monitoring may be necessary if patients are started on medications which may potentially interact with HAART or selumetinib. If changes to medications are necessary the treating physician should consider whether it is in the patient's best interest to remain on study. Any changes to medications should be reported on the Concomitant Medications Form.

In addition:

- Patients should avoid excessive sun exposure and use adequate sunscreen protection if sun exposure is anticipated.
- Patients should avoid grapefruit juice and St John's Wort because of the possible interaction with selumetinib metabolism through inhibition and induction of CYP3A4 respectively and the potential effects they can have on the metabolism of HAART.
- Selumetinib capsules contain vitamin E in the form of D-α-tocopheryl polyethylene glycol 1000 succinate (TPGS), a water-soluble form of vitamin E which acts as a formulation excipient. The maximum daily dose of vitamin E that a study subject may receive from selumetinib is approximately 261.6mg/day. Therefore:
    - Patients should not take any supplemental vitamin E. High doses of vitamin E have been reported to cause bleeding and interrupt blood coagulation processes.
    - Selumetinib/placebo should be administered with caution in patients who are also receiving coumarin anticoagulant medications, e.g. warfarin. These patients should have

their International Normalised Ratio (INR) monitored/ anticoagulant assessments conducted more frequently and the dose of the anticoagulant should be adjusted accordingly,

7.9 Assessments

7.9.1 Pre-treatment Screening Assessments

Within 4 weeks prior to starting trial treatment, the following are required:

- CT scan of thorax, abdomen & pelvis
- Pregnancy test in females of child-bearing potential
- Echo/MUGA.
- Ophthalmologic exam to include slit lamp examination, measurement of visual acuity, assessment of visual fields, assessment of colour vision, and overall visual assessment.
- Tumour biopsy (archived diagnostic material is acceptable if available)

Within 2 weeks prior to starting trial treatment, the following are required:

- Medical history and examination, body weight, WHO performance status, pulse rate & blood pressure (if ≥150/95 mmHg repeat 1 hour later, if still ≥ 150/95 mmHg, the patient is ineligible).
- Document evidence of histologically confirmed KS (archived diagnostic biopsy or fresh biopsy if required), HIV positivity and HAART regimen for past 3 months.
- Blood tests for:
    - Full blood count including Hb, WBC, neutrophils and platelets
    - Clotting studies including INR and Prothrombin time
    - Biochemistry profile including urea, creatinine & electrolytes, liver function tests (bilirubin, ALT, AST and ALP), albumin, total protein, creatine phosphokinase, calcium and phosphate.
    - HIV-1 viral load
    - CD4 count
- Chest X-Ray.
- ECG (to include heart rate, PR, QRS Duration, QT and QTc).

Pre-treatment, day 1, the following are required:

- Medical history and examination, including symptoms and signs, body weight, WHO performance status, pulse rate & blood pressure
- Full examination of oral and gingival mucosa
- Document all oral and cutaneous disease using clinical photographs including a ruler where possible (See ACTG criteria, Appendix 1)
- Blood tests for:
    - Full blood count including Hb, WBC, neutrophils and platelets
    - Clotting studies including INR and Prothrombin time
    - Biochemistry profile including urea, creatinine & electrolytes, liver function tests (bilirubin, ALT, AST and ALP), albumin, total protein, creatine phosphokinase, calcium and phosphate.
    - HIV-1 viral load.
    - CD4 count.
    - Angiogenic biomarkers.
    - Pre-treatment anti-retroviral drug levels (Phase I only).
    - Pregnancy test in females of child-bearing age.

7.9.2 Assessments during Treatment

7.9.2.1 Phase I

Phase I is now complete and the recommended phase II was determined to be 75 mg bd.

7.9.2.2 Phase II

The following assessments will be performed every 3 weeks (+/- 1 day), on day 1 of every cycle:

- Physical examination & weight
- Pulse rate & BP.
- Adverse events using CTCAE version 4.0 criteria (see Appendix 4).
- Blood tests for:
    - Full blood count
    - Biochemistry including urea, creatinine & electrolytes, liver function tests (bilirubin, ALT, AST and ALP), albumin, total protein, creatine phosphokinase, calcium and phosphate.

Blood Samples:

- Angiogenic biomarkers on Cycle 1 Day 1 Pre-treatment, Cycle 1 Day 22/Cycle 2 Day 1, Cycle 2 Day 22/Cycle 3 Day 1, Cycle 4 Day 22/Cycle 5 Day 1 and Cycle 6 Day 22 (all +/- 1 day except Cycle 1 Day 1 Pre-treatment).
- HIV-1 viral load on Cycle 1 Day 1 Pre-treatment, Cycle 1 Day 22/Cycle 2 Day 1, Cycle 2 Day 22/Cycle 3 Day 1, Cycle 4 Day 22/Cycle 5 Day 1 and Cycle 6 Day 22, then at the end of every 3 cycles in patients continuing on selumetinib beyond 6 cycles (all +/- 1 day except Cycle 1 Day 1 Pre-treatment).
- CD4 count on Cycle 1 Day 1 Pre-treatment, Cycle 3 Day 22/Cycle 4 Day 1 and Cycle 6 Day 22, then at the end of every 3 cycles in patients continuing on selumetinib beyond 6 cycles (all +/- 1 day except Cycle 1 Day 1 Pre-treatment).
- PBMC sub-study on Cycle 1 Day 1 Pre-treatment Cycle 3 Day 22/Cycle 4 Day1 and Cycle 6 Day 22 (participating centres only, all +/- 1 day except Cycle 1 Day 1 Pre-treatment).

Further details can be found in the Laboratory Manual.

Additional Assessments:

- Tumour biopsy – End of Cycle 2 (+/- 1 day).
- Disease evaluation on Cycle 2 Day 22/Cycle 3 Day 1, Cycle 4 Day 22/Cycle 5 Day 1 and Cycle 6 Day 22 then at the end of every 2 cycles (all +/- 1 day) in patients continuing selumetinib using ACTG criteria (see Appendix 1), with clinical photographs.
- Echocardiogram or multi gated acquisition (MUGA) scan (measurement of LVEF) – to be performed on Cycles 2 Day 22/Cycle 3 Day 1 and Cycle 4 Day 22/Cycle 5 Day 1 (both +/- 1 day). If a decrease of LVEF of >10% is observed, an additional assessment may be performed at the 30 Day Post-Discontinuation Visit (+/- 3 days) and every 6 weeks thereafter (+/- 7 days) whilst LVEF continues to remain >10% from baseline. Decreases in LVEF from baseline may be investigated according to the algorithm (**Appendix 7**).
- All new dyspnoea AEs or worsening of pre-existing dyspnoea AEs should be followed according to the dyspnoea algorithm (**Appendix 8**).
- Ophthalmologic exam to include slit lamp examination, measurement of visual acuity, assessment of visual fields, assessment of colour vision, and overall visual assessment – to be performed in patients experiencing visual disturbances whilst in the trial as clinically indicated (**Appendix 9**).
- CT imaging of assessable disease (where present) at the end of every 2 cycles (+/- 1 day).

7.9.3 Off Study assessments

The following assessments will be conducted in all patients upon discontinuing selumetinib treatment:

- Patients who have a drop in LVEF>10% from baseline (measured at screening) during the course of selumetinib treatment should have an echocardiogram or MUGA scan performed after permanent discontinuation of selumetinib.
- Patients will have blood tests for full blood count, biochemistry including urea, creatinine & electrolytes, liver function tests (bilirubin, ALT, AST and ALP), albumin, total protein, creatine phosphokinase, calcium and phosphate.

7.9.4 30 Day Post-Discontinuation assessments

The following assessments will be conducted in all patients 30 days (+/- 3 days) after discontinuing selumetinib treatment:

- Patients who have a drop in LVEF>10% from baseline (measured during screening) during the course of selumetinib treatment which persists at the time of discontinuation of selumetinib should have an echocardiogram or MUGA performed to document reversibility (Appendix 71).
- Patients who have clinically significant full blood count or biochemistry laboratory value AEs in the off study assessments should have a further full blood count and biochemistry profile performed.
- AE and Concomitant Medication assessment (end of reporting period).

7.9.5 Ad Hoc Follow-Up assessments

- Patients who have experienced a drop in LVEF>10% from baseline (measured during screening) during the course of selumetinib treatment which persists at the 30 Day Post-Discontinuation visit should have repeat echocardiograms/MUGAs at 6 weekly intervals (+/- 7 days) whilst LVEF continues to remain >10% from baseline (**Appendix 7**).
- Patients who have clinically significant full blood count or biochemistry laboratory value AEs in the off study assessments which persist at the 30 Day Post-Discontinuation visit should have repeat further full blood count and biochemistry profiles performed at 3 weekly intervals whilst these parameters remain above ULN or baseline values.
- Patients experiencing unresolved AEs at the 30 Day Post-Discontinuation visit should have these followed up every 3 weeks until resolved.

7.9.6 Post-Treatment Follow-Up

- Physical examination every 12 weeks (+/- 7 days) for 12 months or until disease progression, whichever is sooner.
- Disease evaluation using ACTG criteria every 12 weeks (+/- 7 days) for 12 months (see Appendix 1), with clinical photographs every 12 weeks (+/- 7 days and on disease response or progression for 12 months or until progression, whichever is sooner.
- If visceral or extensive disease was identified at baseline, CT scans should be done every 12 weeks for 12 months post end of treatment, or until progression, whichever is sooner.

7.9.7 Phase II: Assessments Summary Table

	Screening		Pre-treatment Day 1	Cycle 1 Day 22/Cycle 2 Day 1 (+/- 1 day)	Cycle 2 Day 22/Cycle 3 Day 1 (+/- 1 day)	Cycle 3 Day 22/Cycle 4 Day 1 (+/- 1 day)	Cycle 4 Day 22/Cycle 5 Day 1 (+/- 1 day)	Cycle 5 Day 22/Cycle 6 Day 1 (+/- 1 day)	Cycle 6 Day 22 (+/- 1 day)	Patients continuing Selumetinib (every 3 weeks, +/- 1 day)	Off Study	30-days post discontinuation Selumetinib (+/- 3 days)	Post treatment (every 12 weeks, +/- 7 days)
	Within 4 weeks	Within 2 weeks											
**Physical**													
History & physical examination		X	X	X	X	X	X	X	X	X			X
Disease evaluation and clinical photographs		X			X		X		X	X (end of every 2 cycles)			X
BP		X	X	X	X	X	X	X	X	X			
ECG		X											
Echocardiogram (ECHO)/multi-gated acquisition (MUGA) scan	X				X		X				$X	$X	
Ophthalmologic exam	#X												
**Haematology**													
Full blood count		X	X	X	X	X	X	X	X	X	X		
Clotting Studies		X	X										
**Biochemistry**													
Urea & electrolytes		X	X	X	X	X	X	X	X	X	X		
Liver function tests		X	X	X	X	X	X	X	X	X	X	*X	
Calcium and phosphate		X	X	X	X	X	X	X	X	X	X		
**Radiology**													
Chest X-Ray		X											
CT	X				†X		†X		†X				†X

Phase II Assessments Continued

	Screening		Pre-treatment Day 1	Cycle 1 Day 22/Cycle 2 Day 1 (+/- 1 day)	Cycle 2 Day 22/Cycle 3 Day 1 (+/- 1 day)	Cycle 3 Day 22/Cycle 4 Day 1 (+/- 1 day)	Cycle 4 Day 22/Cycle 5 Day 1 (+/- 1 day)	Cycle 5 Day 22/Cycle 6 Day 1 (+/- 1 day)	Cycle 6 Day 22 (+/- 1 day)	Patients continuing Selumetinib (every 3 weeks, +/- 1 day)	Off Study	30-days post discontinuation Selumetinib (+/- 3 days)	Post treatment (every 12 weeks, +/- 7 days)
	Within 4 weeks	Within 2 weeks											
**Other Investigations**													
HIV-1 viral load		X	X	X	X		X		X	X (end of every 3 cycles)			
CD4 count		X	X			X			X	X (end of every 3 cycles)			
Angiogenic serum biomarkers			X	X	X		X		X				
Tumour biopsy	X				X								
††PBMC Sub-study			X			X			X	X (end of every 3 cycles)			
**Adverse Events**													
Toxicity reporting	Monitor throughout the course of the study												
Concomitant Medications	Monitor throughout the course of the study												

PK –Pharmacokinetics; PBMC – Peripheral Blood Mononuclear Cell. $ required in patients who have a drop in LVEF >10% from baseline; # subsequent assessments to be performed in patients experiencing visual disturbances as clinically indicated; patients with AST, ALT or bilirubin value above ULN or baseline values at the time of the last dose of selumetinib; † required if visceral or extensive nodal disease identified on baseline CT; †† participating centres only.*

7.9.8 Patient Safety Monitoring

Toxicity will be assessed every 21 days (+/- 1 day) whilst on study medication (and weekly during cycle 1 of phase I). Toxicity will be measured according to CTCAE v 4.0 (Appendix 4).

7.10 Evaluation Criteria

7.10.1 Efficacy

Objective tumour response and time to progression will be measured according to the ACTG criteria (Appendix 1).

7.10.2 Measurability of Tumour Lesions at Baseline

Accurate documentation of the size and number of cutaneous lesions may be difficult.
An estimate of the number of lesions is recommended (see Appendix 1 for algorithm). The location of three to five marker lesions with the inclusion of bi-dimensional measurements where possible, should be selected by the Principal Investigator or a delegated clinician, recorded on standard body diagrams showing their relationship to body landmarks.
Documentation of tumour-associated oedema is also important. Photographs are an essential part of the evaluation including photographs of uninvolved areas of skin and allow assessment of changes in lesion colour and nodularity. Rulers must be included in the photographs.

Anonymised photographs, labelled only with patient's RNO, tumour site (labelled A – E as per the Kaposi Evaluation Form), visit and date of photograph, should be uploaded and emailed to the SCART Trial Mailbox within 2 weeks of being taken.

7.10.3 Tumour Response Evaluation

All patients will have their BEST RESPONSE from the start of study treatment until the end of 6 cycles of treatment classified as outlined in Appendix 1.

7.10.4 Frequency of Tumour Re-evaluation

Tumours (cutaneous, oral and visceral) will be re-evaluated every 6 weeks (+/- 1 day) during treatment. After 6 cycles of treatment responding patients will be offered the option of continuing selumetinib in combination with HAART. Following discontinuation of protocol treatment and the 30 Day Post-Discontinuation visit, patients who have not progressed will continue to be re-evaluated every 12 weeks (+/- 7 days) for up to 12 months.

7.10.5 Date of Progression

This is defined as the first day when the ACTG criteria for PD are met.

7.10.6 Reporting of Tumour Response

All patients included in the study must be assessed for response to treatment, even if there is a major protocol treatment deviation or if they are ineligible, or not followed/re-evaluated. Each patient will be assigned one of the following categories: complete response, partial response, stable disease, progressive disease, early death from malignant disease, early death from toxicity, early death from other cause or unknown (not assessable, insufficient data).
Early death is defined as any death occurring before the first per protocol time point of tumour re-evaluation. The responsible investigator will decide if the cause of death is malignant disease, toxicity or other cause.
Patients for whom response is not confirmed will be classified as "unknown", unless they meet the criteria for stable disease (or the criteria for partial response in case of an unconfirmed complete

response). Patients' response will also be classified as "unknown" if insufficient data were collected to allow evaluation per these criteria.

7.10.7 Duration of Tumour Response

Response duration will be measured from the time measurement criteria for CR/PR (whichever is first recorded) are first met until the first date that recurrent or progressive disease is objectively documented.

7.10.8 Progression Free Survival

Progression free survival will be measured from the time of start of treatment until the criteria for progression are met (see ACTG criteria, Appendix 1).

7.11 Patient Follow Up

- All patients will be followed-up on study until death or for 12 months after the end of treatment, whichever is sooner. Every 12 weeks (+/- 7 days) patients will have a physical examination and clinical photographs to assess disease response or progression.
- If visceral or extensive disease was identified at baseline, CT scans should be done every 12 weeks for 12 months post end of treatment, or until progression, whichever is sooner.

7.12 Patient Withdrawal

The Investigator will make every reasonable effort to keep each patient on study. However, if the Investigator removes a patient from the study treatment or if he or she declines further participation final assessments will be performed, if possible. These include a physical examination, laboratory samples, an assessment of AEs and disease response (if appropriate), recording concomitant medication and drug accountability. All the results of the evaluations and observations, together with a description of the reasons for study withdrawal, must be recorded in the CRF.

Patients who are removed from the study due to adverse experiences (clinical or laboratory) will be treated and followed according to accepted medical practice. All pertinent information concerning the outcome of such treatment must be recorded in the CRF.
The following are justifiable reasons for the Investigator to withdraw a patient from study:

- Unacceptable toxicity.
- Unforeseen events: any event which in the judgement of the Investigator makes further treatment inadvisable.
- SAE requiring discontinuation of treatment.
- Withdrawal of consent.
- Serious violation of the study protocol (including persistent patient attendance failure and persistent non-compliance).
- Withdrawal by the Investigator for clinical reasons not related to the study drug treatment.

Where patient is withdrawn early from trial treatment and is not evaluable, additional patients will be recruited to replace them.

8 TRANSLATIONAL RESEARCH

8.1 Objectives

- To relate clinical responses to selumetinib therapy in combination with HAART by evidence of substrate inhibition in tumour tissue by measurement of pERK.

- To investigate compensatory changes to accessory pathways including JNK, p38 MAPK and PI-3K/Akt pathways in tumour tissue.
- To demonstrate changes in expression of circulating pro-angiogenic factors in association with selumetinib therapy in combination with HAART.
- A sub-study using PBMCs will explore whether susceptibility to inhibition of ERK phosphorylation in KS tumour tissue can be predicted by measurement of pERK in peripheral blood mononuclear cells (PBMCs) and further identify changes in accessory MAPK pathways.
- To determine whether these changes modify constitutive or Toll-like receptor (TLR)-induced expression of pro-inflammatory cytokines, type 1 interferons and reactive oxygen species (ROS) in PBMCs and to repeat key findings in tumour specimens.
- To determine whether selumetinib in combination with HAART modifies PBMC susceptibility to apoptosis or levels of apoptotic cells in tumour samples.

8.2 Tissue Samples

Paired tumour biopsies will be collected pre-study (diagnostic biopsy) and at the end of Cycle 2, whenever possible. At the end of Cycle 2, half the sample will be placed in RNALater for storage at -80^{0}C. Half will be fixed in 10% formalin and embedded in paraffin. Immunohistochemistry will be used to measure pERK, down-stream targets of ERK signalling and key substrates in the JNK, p38 MAPK and PI-3K/Akt pathways.

Immunohistochemistry will also assess the expression of angiogenic factors and activated caspase 3 with TUNEL as a marker for apoptosis. Changes in pERK and key members of other cell signalling pathways identified in the sub-study of PBMCs will be assessed by Western blot using protein lysates formed by homogenization of frozen tissue biopsy sections.

8.3 Blood samples

8.3.1 Angiogenic growth factors

Serum will be collected to measure changes in pro-angiogenic growth factors including angiopoietin-(Ang)-2, vascular endothelial growth factor-(VEGF)-A and VEGF-C using commercially available Enzyme-linked immunosorbent assay (ELISA) kits. Interleukin (IL)-6, and other key cytokines identified in the sub-study below of PBMCs will also be measured by ELISA.

- Blood samples will be collected at Cycle 1 Day 1 Pre-treatment, Cycle 1 Day 22/Cycle 2 Day 1, Cycle 2 Day 22/Cycle 3 Day 1, Cycle 4 Day 22/Cycle 5 Day 1 and Cycle 6 Day 22 (all +/- 1 day, except Cycle 1 Day 1 Pre-treatment).
- 8.5mls of blood should be collected in a BD vacutainer no-additive serum tube.
- Samples should be kept at room temperature for 30 minutes before centrifugation to ensure complete clotting.
- Samples should then be centrifuged at 2000g for 20minutes at room temperature.
- Supernatant should be decanted into a new tube and transferred into 5 x 200µl in 0.5ml eppendorf tubes and any remaining serum aliquoted into 1.5ml eppendorf tubes.
- All tubes should be individually labelled and logged with the patient's study number and treatment cycle.
- Samples should be transferred to a -80^{0}C freezer for storage as soon as possible.
- Samples should be transferred to the central laboratory (University of Sheffield) on dry ice (see Laboratory Manual for courier details).

8.3.2 Pharmacokinetics

Pharmacokinetic studies will be performed in phase I only.
The objectives are:

- To describe the pharmacokinetics of selumetinib in combination with HAART. Non compartmental analysis will estimate C_{max}, AUC_{0-24}, clearance and $t_{1/2}$ from peripheral blood measurements of both AZD6244 and its metabolites pre-dose, and at 2 and 6 hours post dose on day 1 and pre-dose day 15. Laboratory bioanalysis will be performed by Quotient BioResearch.
  Peripheral blood will be sent to Lab21 for anti-retroviral drug levels on Cycle 1 Day 1 Pre-treatment, Cycle 1 Day 15 and Cycle 4 Day 22/Cycle 5 Day 1.

Selumetinib drug levels

- Samples to be collected Cycle 1 Day 1 Pre-treatment, 2 hours and 6 hours post dose (after first set of tablets) pre-dose Cycle 1 Day 15.
- 5mls of venous blood should be collected into a K2-EDTA BD vacutainer
- Samples should be inverted several times to ensure adequate mixing.
- Samples should be centrifuged at 2000g for 20minutes at room temperature.
- 2x 500µl of plasma should be aliquoted to **polypropylene** eppendorf tubes.
- Tubes should be individually labeled with the patient's study number and sample time-point.
- Samples should be stored at -20^{0}C.
- 1 x sample to be transferred on dry ice to Quotient Bioresearch for bioanalysis (all four sample time-points day 1 pre-dose, and at 2 and 6 hours post dose and pre-dose day 15 can be shipped together – please refer to the Laboratory Manual for courier details).
- 1 x sample to be stored at the study centre as back-up.

Anti-retroviral drug levels

- Samples to be collected Cycle 1 Day 1 Pre-treatment and Cycle 1 Day 15 and Cycle 4 Day 22/Cycle 5 Day 1.
- 5mls of venous blood should be collected into a K2-EDTA BD vacutainer
- Samples should be inverted several times to ensure adequate mixing.
- Samples should be centrifuged at 2000g for 20 minutes at room temperature.
- 2x 1000 µl of plasma should be aliquoted to 1.5 ml plastic screw top tubes.
- Samples should be stored at -20^{0}C (or below) for 24 hours before sending.
- Samples should be sent to Lab21 for bio-analysis (please refer to the Laboratory Manual for courier details.

8.3.3 PBMC Sub-study

A sub-study will be undertaken in selected centres in 12 patients in total. In this sub-study requiring viable PBMCs, 50 mls of venous blood will be collected in Lithium Heparin tubes at Cycle 1 Day 1 Pre-treatment, Cycle 3 Day 22/Cycle 4 Day 1 and Cycle 6 Day 22 (all +/- 1 day except Cycle 1 Day 1 Pre-treatment) and couriered to Sheffield for analysis (please refer to the Laboratory Manual for courier details). Viable PBMCs obtained by Ficoll-Paque density centrifugation will be cultured in 24 well plates. Samples will be split and aliquots will be lysed to obtain protein for Western blotting to measure levels of pERK in relation to ERK and of phosphorylation of key substrates in the JNK, p38 MAPK and PI-3K/Akt pathways. Changes to down-stream targets of ERK signalling, including c-fos and c-myc will be assessed along with key apoptotic proteins such as Bad and Bcl-2. An aliquot of PBMCs will be challenged with TLR4 and 9 agonists or left untreated and production of IL-1β, tumour necrosis factor-(TNF)-α, IL-6, IL-10, IL-12 and type 1 interferons will be measured by cytometric bead array of culture supernatants. Reactive oxygen species (ROS) production by PBMCs will be measured by flow cytometry

using dichlorofluorescein (DCF) and cell survival assessed by Annexin V-PE/TO-PRO3 staining and flow cytometry.

9 ADVERSE EVENT REPORTING

The collection and reporting of Adverse Events (AEs) will be in accordance with the Medicines for Human Use Clinical Trials Regulations 2004 and its subsequent amendments. Definitions of different types of AE are listed in Appendix 3. The Investigator should assess the seriousness and causality (relatedness) of all AEs experienced by the patient (this should be documented in the source data) with reference to the Investigator Brochure.

9.1 Reporting Requirements

9.1.1 Adverse Events

All medical occurrences which meet the definition of an AE (see Appendix 3 for definition) should be reported. Please note in Phase I this includes all abnormal laboratory findings. In Phase II abnormal laboratory findings need only be recorded if the local investigator deems the finding as clinically significant. A pre-existing condition (including abnormal laboratory finding which meet CTCAE criteria) must not be reported as an AE unless the condition worsens by at least one CTCAE grade during the trial. The condition, however, must be reported on the Screening Case Report Form.

9.1.2 Serious Adverse Events

Investigators should report AEs that meet the definition of an SAE (see Appendix 3 for definition).

9.1.2.1 Monitoring pregnancies for potential Serious Adverse Events

If a patient becomes pregnant on study selumetinib should be discontinued. It is important to monitor the outcome of pregnancies in order to provide SAE data on congenital anomalies or birth defects.

If a patient or their partner becomes pregnant during the SAE reporting period please complete a Pregnancy Notification Form (providing the patient's details) and return to the Trial Office within 30 days. If it is the patient who is pregnant provide outcome data on a follow-up Pregnancy Notification Form. Where the patient's partner is pregnant consent must first be obtained and the patient should be given a Release of Medical Information Form to give to their partner. If the partner is happy to provide information on the outcome of their pregnancy they should sign the Release of Medical Information Form. Once consent has been obtained provide details of the outcome of the pregnancy on a follow-up Pregnancy Notification Form. If appropriate also complete an SAE Form as detailed below.

9.1.3 Reporting period

Details of all AEs will be documented and reported from the date of commencement of protocol defined treatment until 30 days after the last administration of selumetinib.

9.2 Reporting Procedure

9.2.1 Site

9.2.1.1 Adverse Events

AEs should be reported on an AE Form (and where applicable on an SAE Form). An AE Form should be completed at each visit and returned to the Trial Office. AEs will be reviewed using the Common Terminology Criteria for Adverse Events (CTCAE), version 4.0 (see Appendix 4). Any AEs experienced by the patient but not included in the CTCAE should be graded by an Investigator and recorded on the AE Form using a scale of (1) mild, (2) moderate or (3) severe. For each sign/symptom, the highest grade

observed since the last visit should be recorded. When an adverse event changes CTCAE grade it should be recorded as an individual AE.

9.2.1.2 Serious Adverse Events

For more detailed instructions on SAE reporting refer to the SAE Form Completion Guidelines contained in section 5 of the Investigator Site File (ISF). AEs defined as serious and which require reporting as an SAE should be reported on an SAE Form. When completing the form, the Investigator will be asked to define the causality and the severity of the AE which should be documented using the CTCAE version 4.0. On becoming aware that a patient has experienced an SAE, the Investigator (or delegate) must complete, date and sign an SAE Form. The form should be faxed together with a SAE Fax Cover Sheet to the Trial Office using one of the numbers listed below as soon as possible and no later than 24 hours after first becoming aware of the event:

To report an SAE, fax the SAE Form with an SAE Fax Cover Sheet to:
0121 414 3529 or 0121 414 3700

On receipt the Trial Office will allocate each SAE a unique reference number. This number will be transcribed onto the SAE Fax Cover Sheet which will then be faxed back to the site as proof of receipt. If confirmation of receipt is not received within 1 working day please contact the Trial Office. The SAE reference number should be quoted on all correspondence and follow-up reports regarding the SAE. The SAE Fax Cover Sheet completed by the Trial Office should be filed with the SAE Form in the ISF.
For SAE Forms completed by someone other than the Investigator the Investigator will be required to countersign the original SAE Form to confirm agreement with the causality and severity assessments. The form should then be returned to the Trial Office in the post and a copy kept in the ISF. Investigators should also report SAEs to their own Trust in accordance with local practice.

9.2.1.3 Provision of follow-up information

Patients should be followed up until resolution or stabilisation of the event. Follow-up information should be provided on a new SAE Form (refer to the SAE Form Completion Guidelines for further information).

9.2.2 Trial Office

On receipt of an SAE Form seriousness and causality will be determined independently by a Clinical Coordinator. An SAE judged by the Investigator or Clinical Coordinator to have a reasonable causal relationship with the trial medication will be regarded as a Serious Adverse Reaction (SAR). The Clinical Coordinator will also assess all SARs for expectedness. If the event meets the definition of a SAR that is unexpected (i.e. is not defined in the Investigator Brochure) it will be classified as a Suspected Unexpected Serious Adverse Reaction (SUSAR).

9.2.3 Reporting to the Competent Authority and main Research Ethics Committee

9.2.3.1 Suspected Unexpected Serious Adverse Reactions (SUSARs)

The Trial Office will report a minimal data set of all individual events categorised as a fatal or life threatening SUSAR to the Medicines and Healthcare products Regulatory Agency (MHRA) and main Research Ethics Committee (REC) within 7 days. Detailed follow-up information will be provided within an additional 8 days. All other events categorised as SUSARs will be reported within 15 days.

9.2.3.2 Serious Adverse Reactions

The Trial Office will report details of all SARs (including SUSARs) to the MHRA and main REC annually from the date of the Clinical Trial Authorisation, in the form of a Development Safety Update Report (DSUR).

9.2.3.3 Adverse Events
Details of all AEs will be reported to the MHRA on request.

9.2.3.4 Other safety issues identified during the course of the trial
The MHRA and main REC will be notified immediately if a significant safety issue is identified during the trial. Details of all SUSARs and any other safety issue which arises during the course of the trial will be reported to Principal Investigators. A copy of any such correspondence should be filed in the ISF.

9.2.4 Manufacturer of Investigational Medicinal Product (IMP)
All SAEs will be reported to the manufacturer of the IMP, AstraZeneca, by fax within 24 hours of receipt at the SCART Trial Office.

10 DATA HANDLING AND RECORD KEEPING

10.1 Data Collection
The Case Report Form (CRF) will comprise of the following forms:

Standard Forms	Summary of data recorded	Schedule for submission
Eligibility Form.	Confirmation of eligibility and satisfactory staging investigations where necessary.	Within 2 weeks of registration.
Registration Form.	Patient details and Registration Number.	Within 2 weeks of registration.
Baseline Assessment Form.	Details of baseline assessments inc. physical exam, clinical photography, blood pressure, ECG, baseline LVEF, ophthalmological assessment, haematology and biochemistry, tumour biopsy, HIV-1 viral load, CD4 count, PBMC collection (for sub study). HAART regimen.	Within 2 weeks of registration.
Treatment Cycle Forms (separate forms for cycles 1-6).	Details of 21-day cycle inc. physical exam, clinical photography, blood pressure, ECG, haematology and biochemistry, toxicities.	End of every cycle. Within 1 week.
Adverse Events Form.	List of all adverse events.	End of every cycle. Within 2 weeks
Concomitant Medications Form.	List of concomitant medication.	End of every cycle. Within 2 weeks.
Treatment Continuation Forms	Details of additional treatment cycles post completion of 6 cycles.	End of every additional cycle. Within 1 week.
Off Study Form	Details of haematology and biochemistry assessments, potential ECHO, tumour response and no. of completed cycles	Within 2 weeks.
30-Day Post Discontinuation Follow-Up Form	Details of potential biochemistry and ECHO/MUGA assessments, plus follow up of Adverse Events and Concomitant Medications.	Within 2 weeks.
12-Weekly Follow-up Form.	Details of physical exam and clinical photography (every 12 weeks for 12 months post end of treatment).	Annually following completion of treatment.
**Ad Hoc Forms**		

Additional ECHO/MUGA Scan	Details of ECHO or MUGA (to be performed in patients with symptoms (AEs) suggestive of cardiac impairment).	Within 2 weeks.
Additional Ophthalmologic Exam	Details of ophthalmologic exam (to be performed in patients experiencing visual disturbances)	Within 2 weeks.
Additional Haematology/ Clinical Chemistry Assessments	Haematology and biochemistry assessments performed until all AEs are resolved to baseline upon completion of treatment	Within 2 weeks.
Serious Adverse Event Form.	Details of SAEs	Immediately (within 24 hours) upon notification of an SAE.
Post Treatment Adverse Event Follow-Up Form	Record AEs that have not resolved to baseline at 30 Day Post-Discontinuation visit.	Within 2 weeks.
Pregnancy Notification Form.	Record pregnancy of patient or patient's partner	Immediately upon notification of pregnancy.
Treatment Discontinuation Form.	Date and reason for discontinuation.	Within 1 week.
Death Form.	Date and cause of death.	Immediately upon notification of patient's death.
Withdrawal Form.	Date and reason for patient withdrawal.	Immediately upon notification of a patient's decision to withdraw.
Deviation Form.	Completed in the event of deviation from the protocol.	Immediately upon discovering deviation.
**Cycle 1 Forms**		
Copy of Adverse Events Form *	List of all adverse events.	End of Cycle 1. Within 1 week
Copy of Concomitant Medications Form *	List of concomitant medication.	End of Cycle 1. Within 1 week

*Adverse Events Form and Concomitant Medications Form must be photocopied and sent to the Trial Office within 1 week of the End of Cycle 1 (Cycle 1 Day 22 visit).

The CRF must be completed, signed/dated and returned to the Trial Office by the Investigator or an authorised member of the site research team (as delegated on the Site Signature and Delegation Log) within the timeframe listed above.

Entries on the CRF should be made in ballpoint pen, in blue or black ink, and must be legible. Any errors should be crossed out with a single stroke, the correction inserted and the change initialled and dated. If it is not obvious why a change has been made, an explanation should be written next to the change. Data reported on each form should be consistent with the source data or the discrepancies should be explained. If information is not known, this must be clearly indicated on the form. All missing and ambiguous data will be queried. All sections are to be completed before returning.

In all cases it remains the responsibility of the Investigator to ensure that the CRF has been completed correctly and that the data are accurate. The completed originals should be sent to the Trial Office and a copy filed in the ISF. Trial forms may be amended by the Trial Office, as appropriate, throughout the duration of the trial. Whilst this will not constitute a protocol amendment, new versions of the form must be implemented by participating sites immediately on receipt.

10.2 Archiving

It is the responsibility of the Principal Investigator to ensure all essential trial documentation and source records (e.g. signed ICF, ISFs, Pharmacy Files, patients' hospital notes, copies of CRFs etc) at their site are securely retained for at least 15 years after the end of the trial or following the processing of all biological material collected for research, whichever is the later. Do not destroy any documents without prior approval from the CRCTU Document Storage Manager.

11 QUALITY MANAGEMENT

11.1 Site Set-up and Initiation

All sites will be required to sign a Clinical Study Site Agreement prior to participation. In addition all participating Investigators will be asked to sign the necessary agreements and supply a current CV to the Trial Office. All members of the site research team will also be required to sign the Site Signature and Delegation Log, which should be returned to the Trial Office. Prior to commencing recruitment all sites will undergo a process of initiation. Key members of the site research team will be required to attend a meeting covering aspects of the trial design, protocol procedures, Adverse Event reporting, collection and reporting of data and record keeping. Sites will be provided with an Investigator Site File and a Pharmacy File containing essential documentation, instructions, and other documentation required for the conduct of the trial. The Trial Office must be informed immediately of any change in the site research team.

11.2 On-site Monitoring

Monitoring will be carried out as required following a risk assessment and as documented in the SCART Quality Management Plan. Additional on-site monitoring visits may be triggered for example by poor CRF return, poor data quality, low SAE reporting rates, excessive number of patient withdrawals or deviations. If a monitoring visit is required the Trial Office will contact the site to arrange a date for the proposed visit and will provide the site with written confirmation. Investigators will allow the SCART trial staff access to source documents as requested.

11.3 Central Monitoring

Trials staff will be in regular contact with the site research team to check on progress and address any queries that they may have. Trials staff will check incoming Case Report Forms for compliance with the protocol, data consistency, missing data and timing. Sites will be sent Data Clarification Forms requesting missing data or clarification of inconsistencies or discrepancies.
Sites may be suspended from further recruitment in the event of serious and persistent non-compliance with the protocol and/or Good Clinical Practice (GCP), and/or poor recruitment. Any major problems identified during monitoring may be reported to the Trial Management Group and the relevant regulatory bodies. This includes reporting serious breaches of GCP and/or the trial protocol to the main Research Ethics Committee (REC) and the Medicines for Healthcare products Regulatory Agency (MHRA).

11.4 Audit and Inspection

The Investigator will permit trial-related monitoring, audits, ethical review, and regulatory inspection(s) at their site, providing direct access to source data/documents. Sites are also requested to notify the Trial Office of any MHRA inspections.

11.5 Notification of Serious Breaches

In accordance with Regulation 29A of the Medicines for Human Use (Clinical Trials) Regulations 2004 and its amendments the Sponsor of the trial is responsible for notifying the licensing authority in writing of any serious breach of:

- The conditions and principles of GCP in connection with that trial or;
- The protocol relating to that trial, within 7 days of becoming aware of that breach

For the purposes of this regulation, a "serious breach" is a breach which is likely to effect to a significant degree:

- The safety or physical or mental integrity of the subjects of the trial; or
- The scientific value of the trial

Sites are therefore requested to notify the Trial Office of a suspected trial-related serious breach of GCP and/or the trial protocol. Where the Trial Office is investigating whether or not a serious breach has occurred sites are also requested to cooperate with the Trial Office in providing sufficient information to report the breach to the MHRA where required and in undertaking any corrective and/or preventive action.

12 END OF TRIAL DEFINITION

The end of trial is defined as 18 months after closure to recruitment to allow for completion of translational research.

This definition allows completion of the secondary end-point of progression free survival rate at 6 months from commencing treatment. Patients will continue to be followed up beyond 6 months with data collected by the CRCTU to 12 months for supporting evidence for future trial planning. This will allow sufficient time for the completion of protocol procedures, data collection and data input. The Trial Office will notify the MHRA and main REC that the trial has ended and will provide them with a summary of the clinical trial report within 12 months of the end of trial.

Patients responding on selumetinib will be offered to continue on treatment. Patients who have been shown to derive benefit from the selumetinib and HAART combined therapy at one year should be continued on this regimen in an extension study as long as their risk: benefit analysis, in the opinion of the treating physician, favours the continuation of treatment and with approval from AstraZeneca. The number of treatment cycles completed and progression free survival data will be collected by the Trial Office 12 months after the last patient completes study treatment to provide supporting evidence of response for future trial development.

After closure of the trial with the MHRA the Sponsor is no longer required to notify the MHRA and main REC of changes of Principal Investigator. However, sites should continue to notify the Trial Office of changes in Principal Investigator by completing and returning (where required) an Investigator Registration Form together with a current signed and dated CV.
The Trial Office will notify the MHRA and main REC that the trial has ended at the appropriate time and will provide them with a summary of the clinical trial report within 12 months of the end of trial.

13 STATISTICAL CONSIDERATIONS

13.1 Statistical Design

13.1.1 Phase I

- This is a dose finding study using the classic 3 + 3 design with fixed dose increments.
- The primary outcome measure is to identify the safe recommended phase II dose using CTCAE version 4.0 criteria to assess dose limiting toxicity.
- The expected number of patients in phase I will be 12.

13.1.2 Phase II

- This is a single-arm study requiring 25 patients.
- The primary outcome measure is the objective response using ACTG criteria following up to 6 cycles of treatment (see Appendix 1).
- A Simon two stage minimax design (Early Phase Clinical Trials Software Version 1.0b) was used.
- The hypothesis (H_a) under investigation is that the recommended phase II dose will produce an objective response in more than 30% of patients.
- The null hypothesis (H_0) is that the recommended phase II dose will produce an objective response in less than 10% of patients.
- Stage 1 of accrual: 16 response evaluable patients will be entered in the first stage. Using response hypotheses as stated above, we would reject the drug at the end of the first stage of accrual if only 1 or less objective responses were seen. Otherwise, an additional 9 patients will be accrued.
- Stage 2 of accrual: An additional 9 patients will be accrued. We would accept the drug as active if 5 or more objective responses were observed from the 25 patients accrued.
- Significance level and power: The significance level is set at 0.10 (i.e. the probability of incorrectly rejecting H_0 given it is true) and the power is set at 0.90 (i.e. the probability of correctly deciding the regimen is active given the true response rate is greater than 30%).

13.2 Statistical Analysis

13.2.1 Phase I

Phase I consists of a dose finding study in the classic 3 + 3 design with fixed dose increments. Phase I data from monotherapy studies of selumetinib enable an initial dose estimate close to the MTD.
The expected number of patients recruited to phase I is 12.

- A descriptive analysis will be undertaken to describe toxicity per cycle and dose intensity.
- Non compartmental analysis will estimate C_{max}, AUC_{0-24}, clearance and $t_{1/2}$ from peripheral blood measurements of both AZD6244 and its metabolites.
- Percentage changes in anti-retroviral drug levels will be calculated.
- Percentage inhibition of pERK in tumour tissue will be calculated and correlated with tumour response. Changes to downstream proteins, apoptotic pathways and adaptive changes to other MAPK pathways will be assessed.
- Percentage changes in serum angiogenic markers will be calculated and correlated with tumour response.
- Percentage changes in HIV-viral load and CD4 count will be calculated.
- Response rate will be estimated with 90% confidence intervals.

- Number of treatment cycles completed, duration of response and progression free survival at 6 months from commencing treatment will be reported.

13.2.2 Phase II

Phase II will investigate the efficacy of selumetinib in combination with HAART in patients with KS. Patients recruited to the recommended phase II dose in phase I will be included in phase II.
In order to keep the trial the trial size as small as possible a Simon two-stage design will be used for patient accrual.
Phase II will accrue up to a total of 25 response evaluable patients (including those from phase I).
The primary outcome measure of phase II is best objective tumour response up to the end of the 6th cycle. Objective tumour response is as defined by the ACTG criteria (refer to appendix 1 for details). Both CR and PR will count as responses for the purpose of the primary outcome.

- Response rate will be estimated with 90% confidence intervals.
- Toxicity per cycle and dose intensity will be reported.
- Number of treatment cycles completed, duration of response and progression free survival at 6 months from commencing treatment will be reported
- Percentage inhibition of pERK in tumour tissue will be calculated and correlated with tumour response. Changes to downstream proteins, apoptotic pathways and adaptive changes to other MAPK pathways will be assessed.
- Percentage changes in serum angiogenic markers will be calculated and correlated with tumour response.
- Percentage changes in HIV-viral load and CD4 count will be calculated.

14 TRIAL ORGANISATIONAL STRUCTURE

14.1 Sponsor

The sponsor of this trial is the Sheffield Teaching Hospitals NHS Foundation Trust.

14.2 Coordinating Centre

The trial is being conducted under the auspices of the CRCTU, University of Birmingham according to their local procedures.

14.3 Trial Management Group (TMG)

The TMG will consist of the Chief Investigator, Co-Investigators, Trial Co-ordinator and Statistician. The TMG will be responsible for the clinical set-up, on-going management, promotion of the study, and for the interpretation of the results.

14.4 Safety Review Committee

The Safety Review Committee will consist of the Chief Investigator, Co-Investigators, other trial investigators from recruitment centres and the trial team at CRCTU. After completion of each cohort, there will be a teleconference between all Principal Investigators and the CI to discuss toxicity levels, adverse events etc.

14.5 Data Monitoring Committee

A Data Monitoring Committee (DMC) will be established to assess any safety issues and will report to the Trial Management Group. The DMC will convene annually.

14.6 Finance

This is a clinician-initiated and clinician-led trial funded by Cancer Research UK together with a grant from AstraZeneca. AstraZeneca will be providing selumetinib free of charge.

15 ETHICAL CONSIDERATIONS

The trial will be performed in accordance with the recommendations guiding physicians in biomedical research involving human subjects, adopted by the 18th World Medical Association General Assembly, Helsinki, Finland, June 1964, amended at the 48th World Medical Association General Assembly, Somerset West, Republic of South Africa, October 1996 (See **Appendix 2**).
(website: http://www.wma.net/en/30publications/10policies/b3/index.html).

The trial will be conducted in accordance with the Research Governance Framework for Health and Social Care, the applicable UK Statutory Instruments, (which include the Medicines for Human Use Clinical Trials 2004 and subsequent amendments and the Data Protection Act 1998 and Human Tissue Act 2008*)* and the principles of GCP. This trial will be carried out under a Clinical Trial Authorisation in accordance with the Medicines for Human Use Clinical Trials regulations. The protocol will be submitted to and approved by the main REC prior to circulation.

Before any patients are enrolled into the trial, the Principal Investigator at each site is required to obtain local R&D approval. Sites will not be permitted to enrol patients until written confirmation of R&D approval is received by the Trial Office. It is the responsibility of the Principal Investigator to ensure that all subsequent amendments gain the necessary local approval. This does not affect the individual clinicians' responsibility to take immediate action if thought necessary to protect the health and interest of individual patients.

16 CONFIDENTIALITY AND DATA PROTECTION

Personal data recorded on all documents will be regarded as strictly confidential and will be handled and stored in accordance with the Data Protection Act 1998. With the patient's consent, their full name, date of birth, National Health Service (NHS) number, or in Scotland the Community Health Index (CHI), address, post code, hospital number, general practitioner details will be collected at trial entry to assist with long-term follow-up via other health care professionals (e.g. patient's GP). Patients will be identified using only their unique registration number, initials and date of birth on the Case Report Form and correspondence between the Trial Office and the participating site. However patients are asked to give permission for the Trial Office to be sent a copy of their signed Informed Consent Form which will not be anonymised. This will be used to perform in-house monitoring of the consent process and may also be forwarded to other health care professionals involved in the treatment of the patient (e.g. patient's GP).

The Investigator must maintain documents not for submission to the Trial Office (e.g. Patient Identification Logs) in strict confidence. In the case of specific issues and/or queries from the regulatory authorities, it will be necessary to have access to the complete trial records, provided that patient confidentiality is protected.

The Trial Office will maintain the confidentiality of all patients' data and will not disclose information by which patients may be identified to any third party. Representatives of the SCART trial team may be required to have access to patient's notes for quality assurance purposes but patients should be reassured that their confidentiality will be respected at all times.

17 INSURANCE AND INDEMNITY

This trial is a clinician-initiated and clinician-led trial sponsored by Sheffield Teaching Hospitals NHS Foundation Trust.
No provision has been made for indemnity in the event of a claim for non-negligent harm.
In terms of liability, NHS Trust and Non-Trust Hospitals have a duty of care to patients treated, whether or not the patient is taking part in a clinical trial. Compensation is only available via NHS indemnity in the event of clinical negligence being proven.

18 PUBLICATION POLICY

Results of this trial will be submitted for publication in a peer reviewed journal. The manuscript will be prepared by the Trial Management Group (TMG) and authorship will be determined by mutual agreement. Co-authors will include members of the TMG and PIs recruiting more than 10% of the eligible patients enrolled on the trial. Any secondary publications and presentations prepared by Investigators must be reviewed by the TMG. Manuscripts must be submitted to the TMG in a timely fashion and in advance of being submitted for publication, to allow time for review and resolution of any outstanding issues. Authors must acknowledge that the trial was performed with the support of Sheffield Teaching Hospital NHS Foundation Trust. Intellectual property rights will be addressed in the Clinical Study Site Agreement between Sponsor and site.

19 REFERENCE LIST

- Adjei, A. A., R. B. Cohen, *et al*. (2008). "Phase I pharmacokinetic and pharmacodynamic study of the oral, small-molecule mitogen-activated protein kinase kinase 1/2 inhibitor AZD6244 (ARRY-142886) in patients with advanced cancers." J Clin Oncol **26**(13): 2139-46.
- Banerji, U., D. R. Camidge, *et al*. "The first-in-human study of the hydrogen sulfate (Hyd-sulfate) capsule of the MEK1/2 inhibitor AZD6244 (ARRY-142886): a phase I open-label multicenter trial in patients with advanced cancer." Clin Cancer Res **16**(5): 1613-23.
- Bodoky, G., C. Timcheva, *et al*. "A phase II open-label randomized study to assess the efficacy and safety of Selumetinib (AZD6244 [ARRY-142886]) versus capecitabine in patients with advanced or metastatic pancreatic cancer who have failed first-line gemcitabine therapy." Invest New Drugs.
- Bower, M., S. Collins, *et al*. (2008). "British HIV Association guidelines for HIV-associated malignancies 2008." HIV Medicine 9: 336-88.
- Cooley, T., D. Henry, *et al*. (2007). "A randomized, double-blind study of pegylated liposomal doxorubicin for the treatment of AIDS-related Kaposi's sarcoma." Oncologist **12**(1): 114-23.
- Di Lorenzo, G., P. A. Konstantinopoulos, *et al*. (2007). "Management of AIDS-related Kaposi's sarcoma." Lancet Oncol **8**(2): 167-76.
- Health Protection Agency. (2009). "HIV in the United Kingdom: 2009 report." www.hpa.org.uk
- Krown, S. E., C. Metroka, *et al*. (1989). "Kaposi's sarcoma in the acquired immune deficiency syndrome: a proposal for uniform evaluation, response, and staging criteria. AIDS Clinical Trials Group Oncology Committee." J Clin Oncol **7**(9): 1201-7.
- Sharma-Walia, N., H. H. Krishnan, *et al*. (2005). "ERK1/2 and MEK1/2 induced by Kaposi's sarcoma-associated herpesvirus (human herpesvirus 8) early during infection of

target cells are essential for expression of viral genes and for establishment of infection." J Virol **79**(16): 10308-29.
- Stebbing, J., A. Sanitt, *et al*. (2006). "A prognostic index for AIDS-associated Kaposi's sarcoma in the era of highly active antiretroviral therapy." Lancet 367: 1495-502.
- Tulpule, A., J. Groopman, *et al*. (2002). "Multicenter trial of low-dose paclitaxel in patients with advanced AIDS-related Kaposi sarcoma." Cancer **95**(1): 147-54.
- Vart, R. J., L. L. Nikitenko, *et al*. (2007). "Kaposi's sarcoma-associated herpesvirus-encoded interleukin-6 and G-protein-coupled receptor regulate angiopoietin-2 expression in lymphatic endothelial cells." Cancer Res **67**(9): 4042-51.
- Wang, H. W., M. W. Trotter, *et al*. (2004). "Kaposi sarcoma herpesvirus-induced cellular reprogramming contributes to the lymphatic endothelial gene expression in Kaposi sarcoma." Nat Genet **36**(7): 687-93.
- Xie, J., A. O. Ajibade, *et al*. (2008). "Reactivation of Kaposi's sarcoma-associated herpesvirus from latency requires MEK/ERK, JNK and p38 multiple mitogen-activated protein kinase pathways." Virology **371**(1): 139-54.
- Yang, X. and D. Gabuzda (1999). "Regulation of human immunodeficiency virus type 1 infectivity by the ERK mitogen-activated protein kinase signaling pathway." J Virol **73**(4): 3460-6.

20 APPENDICES

20.1 Appendix 1 - AIDS Clinical Trials Group Oncology Committee Documentation of Disease and Definitions of Response

Documentation of Disease

Accurate documentation of the number of lesions may be difficult. An estimate only is required:

None; > 0 ≤ 10; > 10 ≤ 50; or > 50.

Documentation of tumour-associated oedema and tumour nodularity is also important.

The location of three to five body markers and bi-dimensional measurements where possible, should be drawn on standard body diagrams showing their relationship to body landmarks.

Photographs are an essential part of the evaluation including photographs of uninvolved areas of skin.

Evaluation of Response

Complete Response (CR):

The absence of any detectable residual disease, including tumour-associated oedema, persisting for at least 4 weeks.

Partial Response (PR):

A 50% or greater decrease in the number and/or size of previously existing lesions (skin, oral, measurable or evaluable visceral disease) lasting for at least 4 weeks without the appearance of new lesions or the appearance or worsening of tumour-associated oedema or effusions, or an increase of 25% or more in the product of bi-dimensional diameters of any indicator lesion.

A 50% decrease in the sum of the products of perpendicular diameters of bi-dimensionally measurable marker lesions.

Complete flattening of at least 50% of the lesions (i.e., 50% of previously nodular or plaque-like lesions become macules). In those patients with predominantly nodular lesions, flattening to an indurated plaque of 75% or more of the nodules will also be considered a PR.

Whenever possible, responses should be documented with photographs.

Patients with residual tumour-associated oedema or effusion who otherwise meet the criteria for CR will be classified as having a PR.

Stable disease (SD):

Any response not meeting the criteria for progression or PR.

Progressive Disease (PD):

The appearance of new lesions or new sites of disease.

An increase of 25% or more in the size of previously existing lesions.

A change in the character of 25% of more of the skin or oral lesions from macular to plaque-like or nodular.

The development of new or increasing tumour-associated oedema or effusion also represents disease progression.

(Adapted from Krown, Metroka *et al*. 1989).

SCART KAPOSI'S SARCOMA EVALUATION

Patient Number ☐☐☐☐☐☐☐☐ Institution Code ☐☐☐☐

This form is to be used to evaluate KS at baseline

At baseline identify 5 representative lesions (including oral and visceral lesions) to be reproducibly measured on follow up and indicate their location on the schematic below.

If present visceral disease should be included as at least one representative lesion.

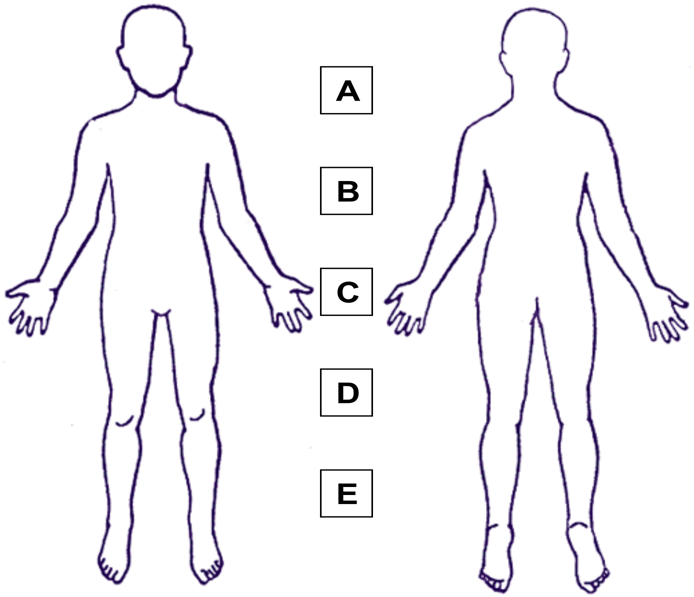

Date	☐☐ ☐☐☐ ☐☐ (d d m m m y y)	BASELINE ASSESSMENT		
Lesion	Perpendicular Diameters (mm)	Product of Diameters (mm²)	Oedema	
			Present? Y/N	Grade? Mild – 1; Mod – 2; Severe - 3
A	☐☐ x ☐☐	☐☐☐☐	☐	☐
B	☐☐ x ☐☐	☐☐☐☐	☐	☐
C	☐☐ x ☐☐	☐☐☐☐	☐	☐
D	☐☐ x ☐☐	☐☐☐☐	☐	☐
E	☐☐ x ☐☐	☐☐☐☐	☐	☐
	Sum of Products (mm²)	☐☐☐☐	Grade total number of KS lesions*	☐

*Grade number of KS lesions: 0 - None
1- >0 ≤10
2 - >10 ≤50
3 - >50

Patient Number [ ][ ][ ][ ][ ][ ][ ][ ] Institution Code [ ][ ][ ][ ]

This form is to be used to document KS at follow up assessments using lesions A-E identified at baseline.

Date	d d m m m y y		Cycle #
Lesion	Perpendicular Diameters (mm)	Product of Diameters (mm²)	
A	x		Evaluation of Nodularity:
B	x		
C	x		Oedema Evaluation:
D	x		
E	x		Overall Clinical Response:
	Sum of Products (mm²)		CR PR SD PD

Complete Response (CR):

The absence of any detectable residual disease, including tumour-associated oedema, persisting for at least 4 weeks.

Partial Response (PR):

A 50% or greater decrease in the number of previously existing lesions (skin, oral, measurable or evaluable visceral disease) lasting for at least 4 weeks without the appearance of new lesions or the appearance or worsening of tumour-associated oedema or effusions, or an increase of 25% or more in the product of bi-dimensional diameters of any indicator lesion.

A 50% decrease in the sum of the products of the perpendicular diameters of bi-dimensionally measurable marker lesions.

Complete flattening of at least 50% of the lesions (i.e. 50% of previously nodular or plaque-like lesions become macules). In those patients with predominantly nodular lesions, flattening to an indurated plaque of 75% or more of the nodules will also be considered a PR.

Whenever possible, responses should be documented with photographs.

Patients with residual tumour-associated oedema or effusion who otherwise meet the criteria for CR will be classified as having a PR.

Stable Disease (SD):

Any response not meeting the criteria for progression or PR.

Progressive Disease (PD):

The appearance of new lesions or new sites of disease
An increase of 25% or more in the size of previously existing lesions
A change in the character of 25% or more of the skin or oral lesions from macular to plaque-like or nodular.
The development of new or increasing tumour-associated oedema or effusion also represents disease progression.

Evaluation of nodularity (cutaneous lesions only):
1 - Greater than or equal to a 50% of raised lesions have become flat compared to baseline or best response
2 - Greater than or equal to a 25% increase in the number of raised lesions compared to baseline or best response (minimum of 5 raised lesions)
3 - None of the above

Evaluation of oedema (cutaneous lesions only):
1 - A resolution of all tumour-associated oedema
2 - No significant increase in tumour-associated oedema compared to baseline or best response
3 - Significant increase in tumour-associated oedema compared to baseline or best response
4 - New tumour-associated oedema compared to baseline or best response
5 - None of the above

20.2 Appendix 2 - WMA Declaration of Helsinki

WORLD MEDICAL ASSOCIATION DECLARATION OF HELSINKI
Recommendations guiding physicians
in biomedical research involving human subjects
Adopted by the 18th World Medical Assembly
Helsinki, Finland, June 1964
and amended by the
29th World Medical Assembly, Tokyo, Japan, October 1975
35th World Medical Assembly, Venice, Italy, October 1983
41st World Medical Assembly, Hong Kong, September 1989
and the
48th General Assembly, Somerset West, Republic of South Africa, October 1996

INTRODUCTION

It is the mission of the physician to safeguard the health of the people. His or her knowledge and conscience are dedicated to the fulfilment of this mission.

The Declaration of Geneva of the World Medical Association binds the physician with the words, "The Health of my patient will be my first consideration," and the International Code of Medical Ethics declares that, "A physician shall act only in the patient's interest when providing medical care which might have the effect of weakening the physical and mental condition of the patient."

The purpose of biomedical research involving human subjects must be to improve diagnostic, therapeutic and prophylactic procedures and the understanding of the aetiology and pathogenesis of disease.

In current medical practice most diagnostic, therapeutic or prophylactic procedures involve hazards. This applies especially to biomedical research.

Medical progress is based on research which ultimately must rest in part on experimentation involving human subjects.

In the field of biomedical research a fundamental distinction must be recognized between medical research in which the aim is essentially diagnostic or therapeutic for a patient, and medical research, the essential object of which is purely scientific and without implying direct diagnostic or therapeutic value to the person subjected to the research.

Special caution must be exercised in the conduct of research which may affect the environment, and the welfare of animals used for research must be respected.

Because it is essential that the results of laboratory experiments be applied to human beings to further scientific knowledge and to help suffering humanity, the World Medical Association has prepared the following recommendations as a guide to every physician in biomedical research involving human subjects. They should be kept under review in the future. It must be stressed that the standards as drafted are only a guide to physicians all over the world. Physicians are not relieved from criminal, civil and ethical responsibilities under the laws of their own countries.

I. BASIC PRINCIPLES

1. Biomedical research involving human subjects must conform to generally accepted scientific principles and should be based on adequately performed laboratory and animal experimentation and on a thorough knowledge of the scientific literature.

2. The design and performance of each experimental procedure involving human subjects should be clearly formulated in an experimental protocol which should be transmitted for consideration, comment and guidance to a specially appointed committee independent of the investigator and the

sponsor provided that this independent committee is in conformity with the laws and regulations of the country in which the research experiment is performed.

3. Biomedical research involving human subjects should be conducted only by scientifically qualified persons and under the supervision of a clinically competent medical person. The responsibility for the human subject must always rest with a medically qualified person and never rest on the subject of the research, even though the subject has given his or her consent.

4. Biomedical research involving human subjects cannot legitimately be carried out unless the importance of the objective is in proportion to the inherent risk to the subject.

5. Every biomedical research project involving human subjects should be preceded by careful assessment of predictable risks in comparison with foreseeable benefits to the subject or to others. Concern for the interests of the subject must always prevail over the interests of science and society.

6. The right of the research subject to safeguard his or her integrity must always be respected. Every precaution should be taken to respect the privacy of the subject and to minimize the impact of the study on the subject's physical and mental integrity and on the personality of the subject.

7. Physicians should abstain from engaging in research projects involving human subjects unless they are satisfied that the hazards involved are believed to be predictable. Physicians should cease any investigation if the hazards are found to outweigh the potential benefits.

8. In publication of the results of his or her research, the physician is obliged to preserve the accuracy of the results. Reports of experimentation not in accordance with the principles laid down in this Declaration should not be accepted for publication.

9. In any research on human beings, each potential subject must be adequately informed of the aims, methods, anticipated benefits and potential hazards of the study and the discomfort it may entail. He or she should be informed that he or she is at liberty to abstain from participation in the study and that he or she is free to withdraw his or her consent to participation at any time. The physician should then obtain the subject's freely-given informed consent, preferably in writing.

10. When obtaining informed consent for the research project the physician should be particularly cautious if the subject is in a dependent relationship to him or her or may consent under duress. In that case the informed consent should be obtained by a physician who is not engaged in the investigation and who is completely independent of this official relationship.

11. In case of legal incompetence, informed consent should be obtained from the legal guardian in accordance with national legislation. Where physical or mental incapacity makes it impossible to obtain informed consent, or when the subject is a minor, permission from the responsible relative replaces that of the subject in accordance with national legislation. Whenever the minor child is in fact able to give consent, the minor's consent must be obtained in addition to the consent of the minor's legal guardian.

12. The research protocol should always contain a statement of the ethical considerations involved and should indicate that the principles enunciated in the present Declaration are complied with.

II. MEDICAL RESEARCH COMBINED WITH PROFESSIONAL CARE (Clinical Research)

1. In the treatment of the sick person, the physician must be free to use a new diagnostic and therapeutic measure, if in his or her judgement it offers hope of saving life, re-establishing health or alleviating suffering.

2. The potential benefits, hazards and discomfort of a new method should be weighed against the advantages of the best current diagnostic and therapeutic methods.

3. In any medical study, every patient - including those of a control group, if any - should be assured of the best proven diagnostic and therapeutic method. This does not exclude the use of inert placebo in studies where no proven diagnostic or therapeutic method exists.

4. The refusal of the patient to participate in a study must never interfere with the physician-patient relationship.

5. If the physician considers it essential not to obtain informed consent, the specific reasons for this proposal should be stated in the experimental protocol for transmission to the independent committee (I, 2).

6. The physician can combine medical research with professional care, the objective being the acquisition of new medical knowledge, only to the extent that medical research is justified by its potential diagnostic or therapeutic value for the patient.

III. NON-THERAPEUTIC BIOMEDICAL RESEARCH INVOLVING HUMAN SUBJECTS (Non-Clinical Biomedical Research)

1. In the purely scientific application of medical research carried out on a human being, it is the duty of the physician to remain the protector of the life and health of that person on whom biomedical research is being carried out.

2. The subject should be volunteers - either healthy persons or patients for whom the experimental design is not related to the patient's illness.

3. The investigator or the investigating team should discontinue the research if in his/her or their judgement it may, if continued, be harmful to the individual.

4. In research on man, the interest of science and society should never take precedence over considerations related to the wellbeing of the subject.

20.3 Appendix 3 - Definition of Adverse Events

Adverse Event

Any untoward medical occurrence in a patient or clinical trial subject administered a medicinal product and which does not necessarily have a causal relationship with this treatment.

Comment:

An AE can therefore be any unfavourable and unintended sign (including abnormal laboratory findings), symptom or disease temporally associated with the use of an investigational medicinal product, whether or not related to the investigational medicinal product.

Adverse Reaction

All untoward and unintended responses to an IMP related to any dose administered.

Comment:

An AE judged by either the reporting Investigator or Sponsor as having causal relationship to the IMP qualifies as an AR. The expression reasonable causal relationship means to convey in general that there is evidence or argument to suggest a causal relationship.

Serious Adverse Event

Any untoward medical occurrence or effect that at any dose:

- Results in death
- Is life-threatening*
- Requires hospitalisation** or prolongation of existing inpatients' hospitalisation
- Results in persistent or significant disability or incapacity
- Is a congenital anomaly/birth defect
- Or is otherwise considered medically significant by the Investigator***

Comments:

The term severe is often used to describe the intensity (severity) of a specific event. This is not the same as serious, which is based on patients/event outcome or action criteria.

* Life threatening in the definition of an SAE refers to an event in which the patient was at risk of death at the time of the event; it does not refer to an event that hypothetically might have caused death if it were more severe.

**Hospitalisation is defined as an unplanned, formal inpatient admission, even if the hospitalisation is a precautionary measure for continued observation. Thus hospitalisation for protocol treatment (e.g. line insertion), elective procedures (unless brought forward because of worsening symptoms) or for social reasons (e.g. respite care) are not regarded as an SAE.

*** Medical judgment should be exercised in deciding whether an AE is serious in other situations. Important AEs that are not immediately life threatening or do not result in death or hospitalisation but may jeopardise the subject or may require intervention to prevent one of the other outcomes listed in the definition above, should be considered serious.

Serious Adverse Reaction

An Adverse Reaction which also meets the definition of a Serious Adverse Event.

Suspected Unexpected Serious Adverse Reaction

A SAR that is unexpected i.e. the nature, or severity of the event is not consistent with the applicable product information.

A SUSAR should meet the definition of an AR, UAR and SAR.

Unexpected Adverse Reaction

An AR, the nature or severity of which is not consistent with the applicable product information (e.g. Investigator Brochure for an unapproved IMP or (compendium of) Summary of Product Characteristics (SPC) for a licensed product).

When the outcome of an AR is not consistent with the applicable product information the AR should be considered unexpected.

20.4 Appendix 4 - Common Toxicity Criteria Gradings

Toxicities will be recorded according to the Common Terminology Criteria for Adverse Events (CTCAE), version 4.0. The full CTCAE document is available on the National Cancer Institute (NCI) website, the following address was correct when this version of the protocol was approved:

http://ctep.cancer.gov/protocolDevelopment/electronic_applications/ctc.htm

20.5 Appendix 5 – New York Heart Association Classification – Stages of Heart Failure

Class	Patient Symptoms
Class I (mild)	No limitation of physical activity. Ordinary physical activity does not cause undue fatigue, palpitation, or dyspnoea (shortness of breath).
Class II (mild)	Slight limitation of physical activity. Comfortable at rest, but ordinary physical activity results in fatigue, palpitation, or dyspnoea.
Class III (moderate)	Marked limitation of physical activity. Comfortable at rest, but less than ordinary activity causes fatigue, palpitation, or dyspnea.
Class IV (severe)	Unable to carry out any physical activity without discomfort. Symptoms of cardiac insufficiency at rest. If any physical activity is undertaken, discomfort is increased.

20.6 Appendix 6 – Canadian Cardiovascular Society Grading of Angina Pectoris

Grade	Description
I	Ordinary physical activity does not cause angina, such as walking and climbing stairs. Angina with strenuous or rapid or prolonged exertion at work or recreation
II	Slight limitation of ordinary activity. Walking or climbing stairs rapidly, walking uphill, walking or stair climbing after meals, or in cold, or in wind, or under emotional stress, or only during the few hours after awakening. Walking more than two blocks on the level and climbing more than one flight of ordinary stairs at a normal pace and in normal conditions
III	Marked limitation of ordinary physical activity. Walking one or two blocks on the level and climbing one flight of stairs in normal conditions and at normal pace
IV	Inability to carry on any physical activity without discomfort, anginal syndrome may be present at rest

20.7 Appendix 7 – Guidance for management of patients with a reduction in LVEF

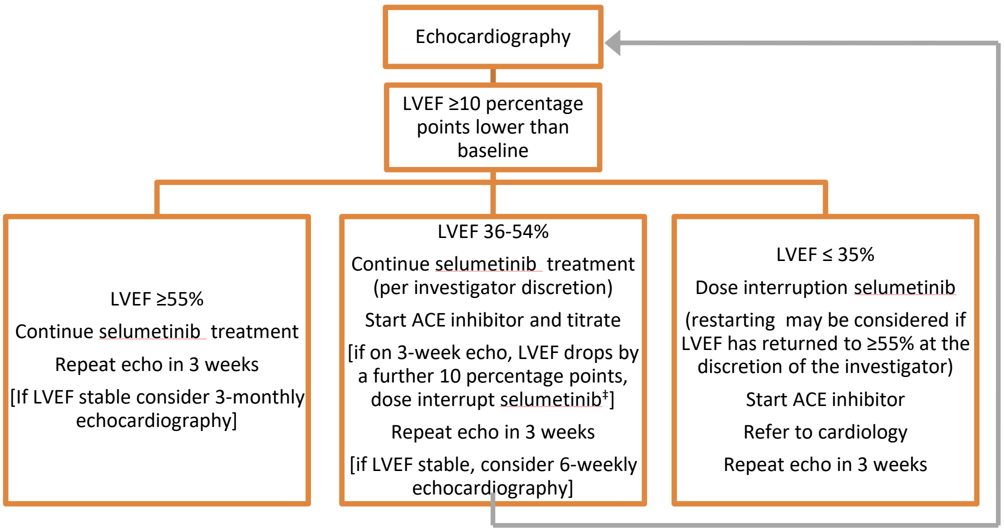

Echo: Echocardiography
LVEF: Left ventricular ejection fraction
Version: Final 2.0 28Sept 2012

20.8 Appendix 8 – Guidance for investigation of patients with new/worsening dyspnoea* (not considered related to disease under study)

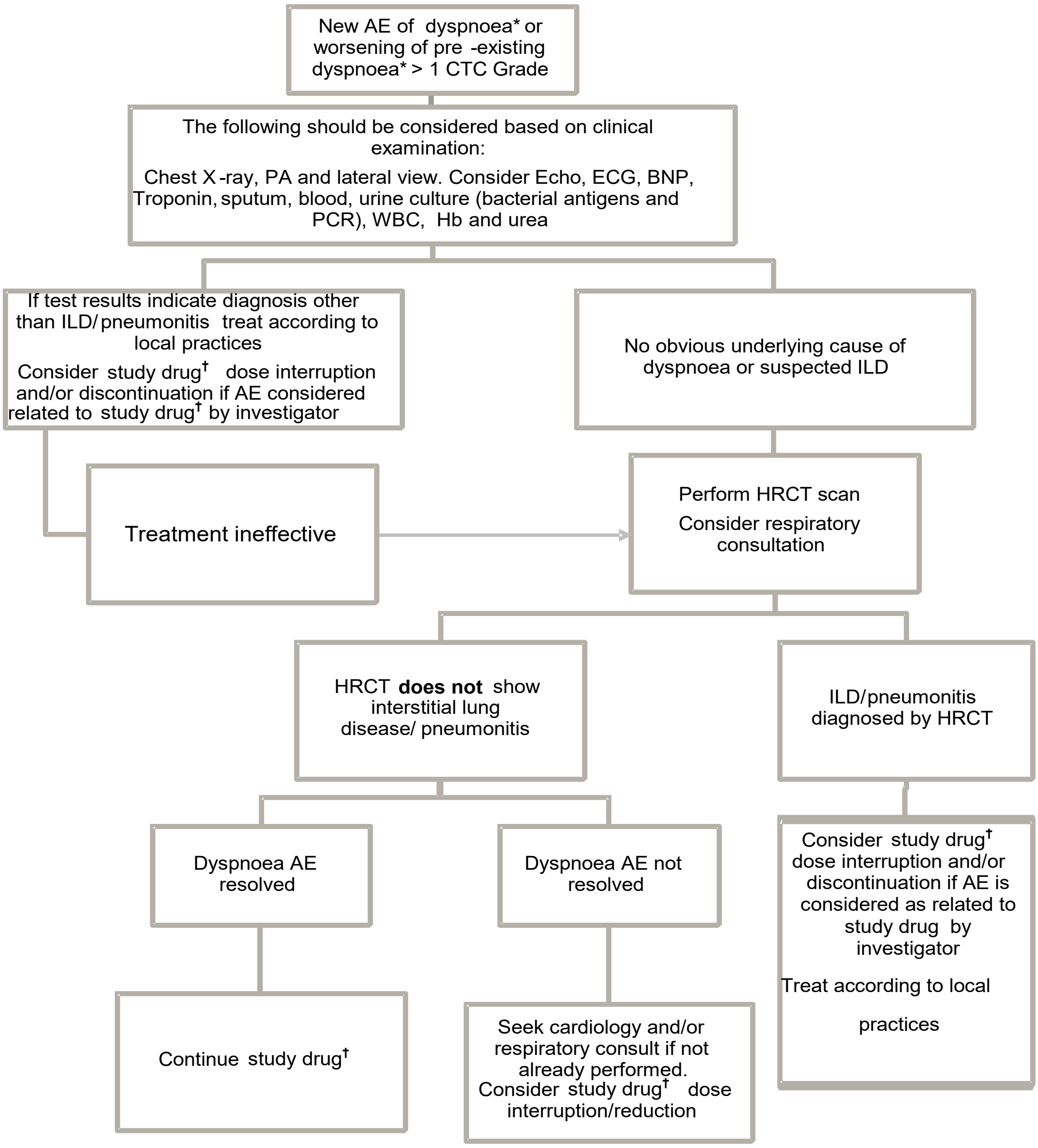

BNP: Brain Natriuretic
CTC: National Cancer Institute common toxicology
Echo: Echocardiography
Hb: Haemoglobin
HRCT: High resolution computerised tomography

ILD: Interstitial Lung
PA: posteroanterior
PCR: Polymerase chain
WBC: White blood cells
Version: Final 3.0 23-Jul-2013

* Not considered related to disease under study

† Includes selumetinib

20.9 Appendix 9 – Guidance for management of patients with visual symptoms

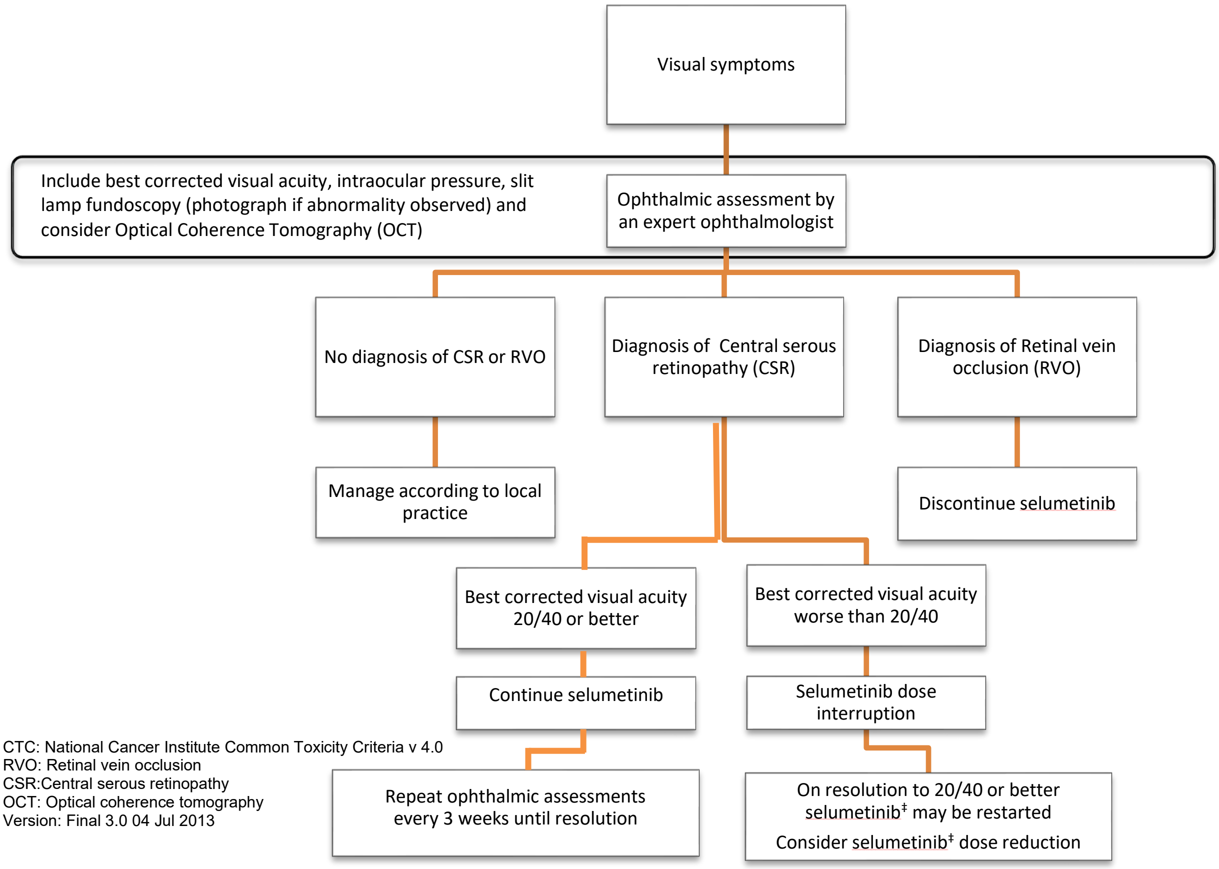

CTC: National Cancer Institute Common Toxicity Criteria v 4.0
RVO: Retinal vein occlusion
CSR:Central serous retinopathy
OCT: Optical coherence tomography
Version: Final 3.0 04 Jul 2013

20.10 Appendix 10 – Guidance for management of creatine kinase elevation

To reduce possible consequences associated with creatine kinase (creatine phosphokinase, CK) elevation that may be arising from a muscular source, the management guideline described below should be followed:

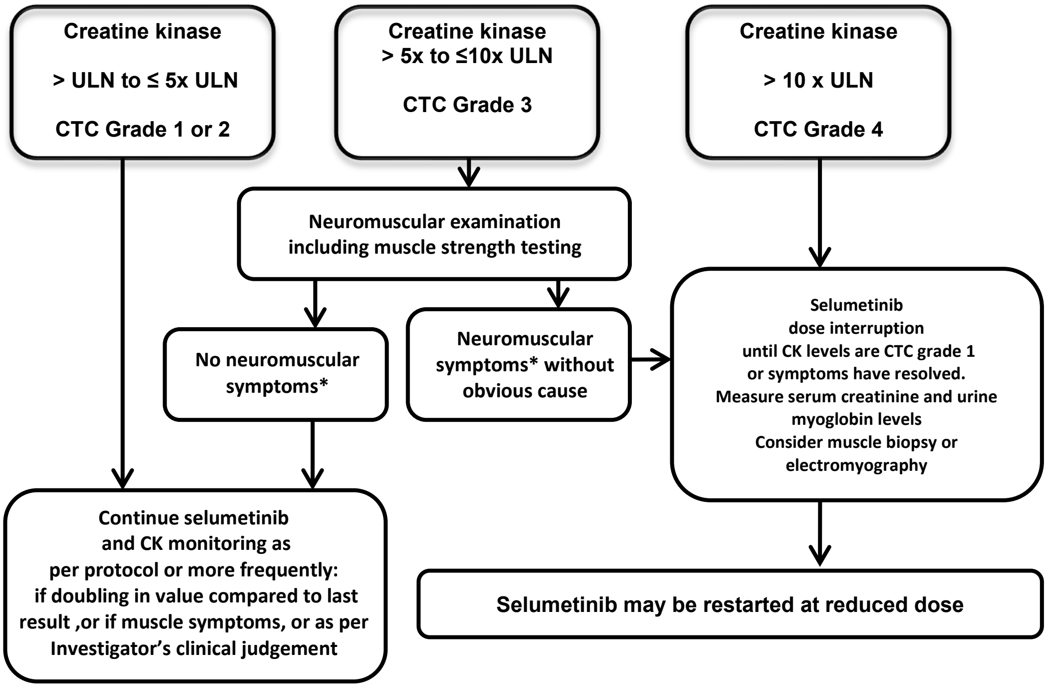

* Muscular weakness, pain or tenderness

Version 3.0
12-Sept-2013

20.11 Appendix 11 – Guidance for the management of patients with diarrhoea

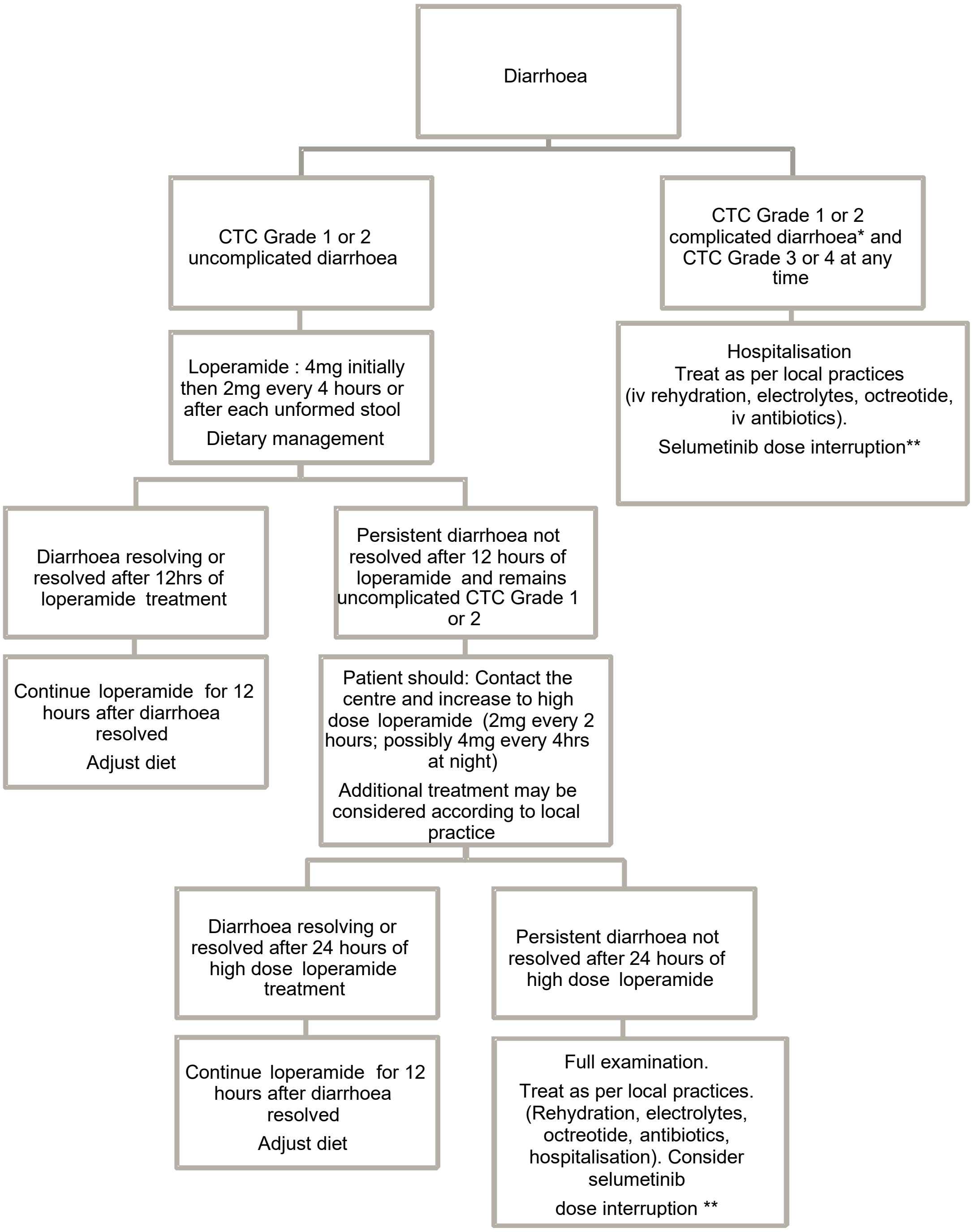

*Diarrhoea becomes complicated by associated vomiting or inability to take oral fluids; marked abdominal distension or cramping; bloody stools, fever or symptoms of hypotension

****Consider interruption or delay of combination anticancer agent if applicable**

Document version: Final 2.0 28Sept2012

20.12 Appendix 12 – Guidance for the management of patients with rash

Recommendations to start on day 1 of treatment with selumetinib and for the duration of treatment

- Use skin moisturiser (thick, alcohol-free) at bedtime
- Avoid excessive exposure to sunlight
- Use sunglasses/sunscreen (PABA-free, SPF ≥15; UVA and UVB protection) as needed
- Use of topical retinoids or benzoyl peroxide is not recommended

CTC Grade 1 rashes

Mild or moderate strength topical steroid

and/or topical antibiotic

CTC Grade 2 rashes

Moderate strength topical steroid

and oral antibiotic

CTC grade ≥3 rashes
CTC grade 2 rashes considered by the patient to be intolerable

Moderate strength topical steroid

and oral antibiotic
(consider broad spectrum/gram negative cover if infection suspected)

Consider referral to a dermatologist: manage rash per recommendation

Interrupt selumetinib until rash improves to grade 2 or less

Selumetinib may be restarted at original dose or reduced at the discretion of the

Table 1: Example topical steroids and antibiotics (use according to local guidelines)

Topical steroids moderate strength	Triamcinolone acetonide 0.025%<br>Fluticasone proprionate 0.05%	Desonide 0.05%<br>Aclometasone 0.05%
Topical antibiotics	Clindamycin 1 - 2%<br>Metronidazole 1%	Erythromycin 1% - 2%<br>Silver sulphadiazine 1%

Oral antibiotics	Doxycycline 100 mg bd mg bd	Minocycline 100 mg bd Oxytetracycline 500
