# Supplementary material for: Selumetinib in Combination with Anti Retroviral Therapy in HIV-associated Kaposi sarcoma (SCART): an open-label, multicentre, phase I/II trial
Source: BMC Cancer. 2025 Mar 19;25:505. doi: 10.1186/s12885-025-13890-x (PMC11921695; doi:10.1186/s12885-025-13890-x)
Supplement: Supplementary file 2 — Supplementary appendix 2. Patients’ antiretroviral therapy at time of trial registration [file 12885_2025_13890_MOESM2_ESM.docx]

# Supplementary appendix 2 – Antiretroviral therapy at time of trial registration for patients who received any study treatment

| **Selumetinib starting dose**  **(mg bd)** | **cART Drug Name** | **Drug Start Date** | **Frequency** |
| --- | --- | --- | --- |
| 50 | Emtricitabine/Tenofovir  Efavirenz | 16-Feb-10  16-Feb-10 | OD  OD |
| 50 | Darunavir | 21-Dec-11 | OD |
|  | Emtricitabine/Tenofovir | 21-Dec-11 | OD |
|  | Ritonavir | 21-Dec-11 | OD |
| 50 | Lopinavir | 4-Aug-08 | BD |
|  | Ritonavir | 4-Aug-08 | Missing |
| 50 | Darunavir | 11-Oct-11 | OD |
|  | Ritonavir | 11-Oct-11 | OD |
|  | Emtricitabine/Tenofovir | 11-Oct-11 | OD |
| 75 | Efavirenz | Missing | OD |
|  | Abacavir/Lamivudine | Missing | OD |
| 75 | Emtricitabine/Tenofovir/  Efavirenz | 19-Apr-11 | OD |
| 75 | Lopinavir/Ritonavir | 7-Sep-07 | BD |
|  | Emtricitabine/Tenofovir | 7-Sep-07 | OD |
| 75 | Emtricitabine/Tenofovir | 3-Aug-13 | OD |
|  | Raltegravir | 3-Aug-13 | BD |
| 75 | Nevirapine | 10-Feb-10 | OD |
|  | Tenofovir | 10-Feb-10 | OD |
|  | Emtricitabine | 10-Feb-10 | OD |
| 75 | Ritonavir | 15-Aug-10 | OD |
|  | Darunavir | 15-Aug-10 | OD |
|  | Emtricitabine | 15-Aug-10 | OD |
|  | Tenofovir | 15-Aug-10 | OD |
| 75 | Efavirenz | 30-Jun-10 | OD |
|  | Tenofovir | 30-Jun-10 | OD |
|  | Emtricitabine | 30-Jun-10 | OD |
| 75 | Emtricitabine/Tenofovir | 15-Oct-12 | OD |
|  | Maraviroc | 15-Oct-12 | OD |
| 75 | Efavirenz | 12-May-14 | OD |
|  | Emtricitabine | 12-May-14 | OD |
|  | Tenofovir | 12-May-14 | OD |
| 75 | Maraviroc | 24-Feb-14 | BD |
|  | Tenofovir | 10-Jul-08 | OD |
|  | Emtricitabine | 10-Jul-08 | OD |
|  | Efavirenz | 10-Jul-08 | OD |
| 75 | Emtricitabine/Tenofovir | 15-Aug-09 | OD |
|  | Atazanavir | 15-Aug-09 | OD |
|  | Ritonavir | 15-Aug-09 | OD |
| 75 | Darunavir | 10-Jan-11 | OD |
|  | Emtricitabine/Tenofovir | 10-Jan-11 | OD |
|  | Ritonavir | 10-Jan-11 | OD |

BD, twice daily; cART, combination antiretroviral therapy; OD, once daily.
