# Supplementary material for: Selumetinib in Combination with Anti Retroviral Therapy in HIV-associated Kaposi sarcoma (SCART): an open-label, multicentre, phase I/II trial
Source: BMC Cancer. 2025 Mar 19;25:505. doi: 10.1186/s12885-025-13890-x (PMC11921695; doi:10.1186/s12885-025-13890-x)
Supplement: Supplementary file 3 — Supplementary appendix 3. The AIDS Clinical Trials Group (ACTG) criteria [file 12885_2025_13890_MOESM3_ESM.docx]

# Supplementary appendix 3 – AIDS Clinical Trials Group (ACTG) Criteria

Adapted from Krown SE, Metroka C, Wernz JC. Kaposi’s sarcoma in the acquired immune deficiency syndrome: a proposal for uniform evaluation, response, and staging criteria. AIDS Clinical Trials Group Oncology Committee. J Clin Oncol. 1989;7(9):1201-7.

## Documentation of Disease

Accurate documentation of the number of lesions may be difficult. An estimate only is required:

None; > 0 ≤ 10; > 10 ≤ 50; or > 50.

Documentation of tumour-associated oedema and tumour nodularity is also important.

The location of three to five body markers and bi-dimensional measurements, where possible, should be drawn on standard body diagrams showing their relationship to body landmarks.

Photographs are an essential part of the evaluation including photographs of uninvolved areas of skin.

## Evaluation of Response

**Complete Response (CR):**

The absence of any detectable residual disease, including tumour-associated oedema, persisting for at least 4 weeks.

**Partial Response (PR):**

A 50% or greater decrease in the number and/or size of previously existing lesions (skin, oral, measurable or evaluable visceral disease) lasting for at least 4 weeks without the appearance of new lesions or the appearance or worsening of tumour-associated oedema or effusions, or an increase of 25% or more in the product of bi-dimensional diameters of any indicator lesion.

A 50% decrease in the sum of the products of perpendicular diameters of bi-dimensionally measurable marker lesions.

Complete flattening of at least 50% of the lesions (i.e., 50% of previously nodular or plaque-like lesions become macules). In those patients with predominantly nodular lesions, flattening to an indurated plaque of 75% or more of the nodules will also be considered a PR.

Whenever possible, responses should be documented with photographs.

Patients with residual tumour-associated oedema or effusion who otherwise meet the criteria for CR will be classified as having a PR.

**Stable disease (SD):**

Any response not meeting the criteria for progression or PR.

**Progressive Disease (PD):**

The appearance of new lesions or new sites of disease.

An increase of 25% or more in the size of previously existing lesions.

A change in the character of 25% or more of the skin or oral lesions from macular to plaque-like or nodular.

The development of new or increasing tumour-associated oedema or effusion also represents disease progression.
