# Supplementary material for: Selumetinib in Combination with Anti Retroviral Therapy in HIV-associated Kaposi sarcoma (SCART): an open-label, multicentre, phase I/II trial
Source: BMC Cancer. 2025 Mar 19;25:505. doi: 10.1186/s12885-025-13890-x (PMC11921695; doi:10.1186/s12885-025-13890-x)
Supplement: Supplementary file 4 — Supplementary appendix 4. Description of the pharmacokinetic and pharmacodynamic assessments, and sample collection and processing methods [file 12885_2025_13890_MOESM4_ESM.docx]

# Supplementary appendix 4 – Pharmacokinetic and pharmacodynamic methods

Pharmacokinetic and pharmacodynamic assessments

The following blood samples were taken to analyse pharmacokinetics and pharmacodynamics during the SCART trial:

- Selumetinib and N-desmethyl selumetinib plasma levels on cycle 1 day 1 at 2- and 6-hours post-treatment, and cycle 1 day 15 pre-treatment.
- Trough cART plasma levels on cycle 1 day 1 pre-selumetinib treatment, cycle 1 day 15, and end of cycle 4
- HIV-1 viral load on cycle 1 day 1 pre-treatment, cycle 2 day 1, cycle 3 day 1, cycle 5 day 1 and end of cycle 6, then at the end of every subsequent three cycles in patients continuing on selumetinib beyond 6 cycles (all ±1 day except cycle 1 day 1 pre-treatment)
- CD4 count on cycle 1 day 1 pre-treatment, cycle 4 day 1 and end of cycle 6, then at the end of every subsequent three cycles in patients continuing on selumetinib beyond 6 cycles (all ±1 day except cycle 1 day 1 pre-treatment)
- Circulating angiogenic biomarkers on cycle 1 day 1 pre-treatment, cycle 2 day 1, cycle 3 day 1, cycle 5 day 1 and end of cycle 6 (all ±1 day except cycle 1 day 1 pre-treatment).

In addition, paired tumour biopsies (fresh and formalin-fixed) were collected pre-treatment (archived material permitted) and end of cycle 2 (±1 day) for planned RNA-sequencing and protein expression analysis, including assessment of phosphorylated (p)ERK levels in tumour tissue and other downstream markers. Unfortunately, few tumour biopsies were performed, and the quantity and quality of tumour material collected prohibited meaningful analysis.

Sample collection and processing

Pharmacokinetic analyses

For selumetinib and N-desmethyl analysis, blood plasma was prepared from 5 ml of venous blood collected in K2-EDTA BD vacutainer tubes from kits provided by Covance. Samples were centrifuged at 2000 *g* for 20 minutes at room temperature, frozen at sites, packed in dry ice and sent to Quotient Bioresearch Ltd (Cambridge, UK) where they were stored at -20°C. Liquid chromatography-tandem mass spectrometry (LC-MS/MS) with solid-phase extraction was performed according to Quotient’s validated method (MWI0402, version 2). Samples were analysed over the calibration range of 2.00 - 2000 ng/mL for selumetinib and 2.00 – 500 ng/mL for the metabolite N-desmethyl.

Therapeutic drug monitoring of the cART therapies was undertaken by Lab 21 Ltd (Cambridge, UK). Blood plasma was prepared from 5 ml of venous blood collected in K2-EDTA BD vacutainer tubes from kits provided by Covance. Samples were centrifuged at 2000 *g* for 20 minutes at room temperature, frozen at sites, packed in dry ice and sent to Lab 21 Ltd (Cambridge, UK) where they were stored at -20°C prior to analysis.

Pharmacodynamic analyses

HIV-1 viral load and CD4 counts were measured as per standard of care at hospital sites. To measure the concentration of angiogenesis markers, 8.5 ml blood was collected in BD vacutainer no-additive serum tubes. Samples were kept at room temperature for 30 minutes before centrifugation at 2000 *g* for 20 minutes at room temperature. Serum samples were then stored at -80^o^C prior to analysis. Serum Ang-1, Ang-2 and TIE levels were assessed using commercial ELISAs (Thermo Fisher Scientifc hAng-1 (EHANGPT1), R&D DuoSet Ang-2 (DY623), eBioscience hTIE2 (BMS2042)), according to manufacturer instructions at laboratories at the University of Sheffield.
