# Supplementary material for: Selumetinib in Combination with Anti Retroviral Therapy in HIV-associated Kaposi sarcoma (SCART): an open-label, multicentre, phase I/II trial
Source: BMC Cancer. 2025 Mar 19;25:505. doi: 10.1186/s12885-025-13890-x (PMC11921695; doi:10.1186/s12885-025-13890-x)
Supplement: Supplementary file 5 — Supplementary appendix 5. Antiretroviral drug levels, as well as selumetinib and N-desmethyl selumetinib levels in plasma collected as part of the phase I pharmacokinetic analysis [file 12885_2025_13890_MOESM5_ESM.docx]

# Supplementary appendix 5 – Phase I pharmacokinetic data

Table S5A – Antiretroviral drug levels

| **Selumetinib Starting Dose (mg bd)** | **Drug** | **Time Point** | **Concentration (ng/ml)** | **% Change** |
| --- | --- | --- | --- | --- |
|  |  | Cycle 1 Day 1 | 4545 |  |
|  | Efavirenz | Cycle 1 Day 15 | 4180 | -8.0 |
|  |  | Cycle 5 Day 1 | 6257 | 37.7 |
|  |  | Cycle 1 Day 1 | 331 |  |
| 50 | Emtricitabine | Cycle 1 Day 15 | 335 | 1.2 |
|  |  | Cycle 5 Day 1 | 375 | 13.3 |
|  |  | Cycle 1 Day 1 | 90 |  |
|  | Tenofovir | Cycle 1 Day 15 | 51 | -43.3 |
|  |  | Cycle 5 Day 1 | 55 | -38.9 |
|  |  | Cycle 1 Day 1 | 7284 |  |
|  | Darunavir | Cycle 1 Day 15 | 4208 | -42.2 |
|  |  | Cycle 5 Day 1 | 7628 | 4.7 |
|  |  | Cycle 1 Day 1 | 1586 |  |
|  | Emtricitabine | Cycle 1 Day 15 | 1935 | 22.0 |
| 50 |  | Cycle 5 Day 1 | 2281 | 43.8 |
|  |  | Cycle 1 Day 1 | 516 |  |
|  | Ritonavir | Cycle 1 Day 15 | 462 | -10.5 |
|  |  | Cycle 5 Day 1 | 328 | -36.4 |
|  |  | Cycle 1 Day 1 | 133 |  |
|  | Tenofovir | Cycle 1 Day 15 | 234 | 75.9 |
|  |  | Cycle 5 Day 15 | 230 | 72.9 |
|  |  | Cycle 1 Day 1 | 8366 |  |
|  | Lopinavir | Cycle 1 Day 15 | 8715 | 4.2 |
| 50 |  | Cycle 5 Day 1 | 11555 | 38.1 |
|  |  | Cycle 1 Day 1 | Missing |  |
|  | Ritonavir | Cycle 1 Day 15 | 575 |  |
|  |  | Cycle 5 Day 1 | 566 |  |
|  | Darunavir | Cycle 1 Day 1 | 1674 |  |
|  |  | Cycle 1 Day 15 | 7690 | 359.4 |
|  | Emtricitabine | Cycle 1 Day 1 | 55 |  |
| 50 |  | Cycle 1 Day 15 | 203 | 269.1 |
|  | Ritonavir | Cycle 1 Day 1 | 94 |  |
|  |  | Cycle 1 Day 15 | 246 | 161.7 |
|  | Tenofovir | Cycle 1 Day 1 | 15 |  |
|  |  | Cycle 1 Day 15 | 43 | 186.7 |
|  | Abacavir | Cycle 1 Day 1 | 1060 |  |
|  |  | Cycle 1 Day 15 | 246 | -76.8 |
| 75 | Efavirenz | Cycle 1 Day 1 | 3378 |  |
|  |  | Cycle 1 Day 15 | 3139 | -7.1 |
|  | Lamivudine | Cycle 1 Day 1 | 817 |  |
|  |  | Cycle 1 Day 15 | 512 | -37.3 |
|  |  | Cycle 1 Day 1 | 577 |  |
|  | Emtricitabine | Cycle 1 Day 15 | 755 | 30.8 |
|  |  | Cycle 5 Day 1 | 646 | 12.0 |
|  |  | Cycle 1 Day 1 | 9586 |  |
| 75 | Lopinavir | Cycle 1 Day 15 | 8050 | -16.0 |
|  |  | Cycle 5 Day 1 | 9296 | -3.0 |
|  |  | Cycle 1 Day 1 | 84 |  |
|  | Tenofovir | Cycle 1 Day 15 | 123 | 46.4 |
|  |  | Cycle 5 Day 1 | 135 | 60.7 |
|  | Emtricitabine | Cycle 1 Day 1 | 272 |  |
|  |  | Cycle 1 Day 15 | 214 | -21.3 |
| 75 | Raltegravir | Cycle 1 Day 1 | 65 |  |
|  |  | Cycle 1 Day 15 | 1413 | 2073.8 |
|  | Tenofovir | Cycle 1 Day 1 | 30 |  |
|  |  | Cycle 1 Day 15 | 46 | 53.3 |
|  | Emtricitabine | Cycle 1 Day 1 | 16 |  |
|  |  | Cycle 5 Day 1 | 960 | 5900 |
|  |  | Cycle 1 Day 1 | 46 |  |
| 75 | Maraviroc | Cycle 1 Day 15 | 183 | 297.8 |
|  |  | Cycle 5 Day 1 | 157 | 241.3 |
|  | Tenofovir | Cycle 1 Day 1 | 60 |  |
|  |  | Cycle 5 Day 1 | 104 | 73.3 |
|  | Atazanavir | Cycle 1 Day 1 | 758 |  |
|  |  | Cycle 1 Day 15 | 5797 | 664.8 |
|  | Emtricitabine | Cycle 1 Day 1 | 437 |  |
| 75 |  | Cycle 1 Day 15 | 837 | 91.5 |
|  | Ritonavir | Cycle 1 Day 1 | 364 |  |
|  |  | Cycle 1 Day 15 | 1585 | 335.4 |
|  | Tenofovir | Cycle 1 Day 1 | 130 |  |
|  |  | Cycle 1 Day 15 | 239 | 125.4 |
|  |  | Cycle 1 Day 1 | 3487 |  |
|  | Darunavir | Cycle 1 Day 15 | 9048 | 159.5 |
|  |  | Cycle 5 Day 1 | 6164 | 76.8 |
|  |  | Cycle 1 Day 1 | 492 |  |
|  | Emtricitabine | Cycle 1 Day 15 | 489 | -0.6 |
| 75 |  | Cycle 5 Day 1 | 453 | -7.9 |
|  |  | Cycle 1 Day 1 | 342 |  |
|  | Ritonavir | Cycle1 Day 15 | 564 | 64.9 |
|  |  | Cycle 5 Day 1 | 431 | 26.0 |
|  |  | Cycle 1 Day 1 | 90 |  |
|  | Tenofovir | Cycle 1 Day 15 | 40 | -55.6 |
|  |  | Cycle 5 Day 15 | 143 | 58.9 |

Table S5B – Selumetinib plasma levels

| **Selumetinib Starting Dose**  **(mg bd)** | **Selumetinib Concentration (ng/ml)** | | | |
| --- | --- | --- | --- | --- |
|  | **Day** **1** | **2 Hours** | **6 Hours** | **Day 15** |
|  | **Pre-Dose** | **Post-Dose** | **Post-Dose** | **Pre-Dose** |
| 50 | BLQ | 201 | 26.5 | 36.7 |
| 50 | BLQ | 304 | 48.5 | 124 |
| 50 | BLQ | 238 | 47.9 | 148 |
| 50 | BLQ | 274 | 98.1 | 98.1 |
| 75 | BLQ | 1040 | Missing | 113 |
| 75 | BLQ | 1280 | 249 | 121 |
| 75 | BLQ | 1250 | 180 | 391 |
| 75 | BLQ | 361 | 284 | 43.5 |
| 75 | BLQ | 247 | 452 | 302 |
| 75 | BLQ | 504 | 66.1 | 374 |

BLQ, below the level of quantification.

**Table S5C – N-Desmethyl plasma levels**

| **Selumetinib Starting Dose (mg bd)** | **N-Desmethyl Concentration (ng/ml)** | | | |
| --- | --- | --- | --- | --- |
|  | **Day** **1** | **2 Hours** | **6 Hours** | **Day 15** |
|  | **Pre-Dose** | **Post-Dose** | **Post-Dose** | **Pre-Dose** |
| 50 | BLQ | 5.42 | 2 | 2 |
| 50 | BLQ | 31.7 | 5.7 | 12 |
| 50 | BLQ | 34.8 | 6.2 | 17.2 |
| 50 | BLQ | 15.8 | 10 | 6.5 |
| 75 | BLQ | 26.7 | Missing | 4.8 |
| 75 | BLQ | 40.6 | 6 | 2.1 |
| 75 | BLQ | 51.9 | 14.6 | 6.2 |
| 75 | BLQ | 10.5 | 9.3 | 2 |
| 75 | BLQ | 20.2 | 33.8 | 23.8 |
| 75 | BLQ | 40.8 | 5 | 17.2 |

BLQ, below the level of quantification.
