# Supplementary material for: Selumetinib in Combination with Anti Retroviral Therapy in HIV-associated Kaposi sarcoma (SCART): an open-label, multicentre, phase I/II trial
Source: BMC Cancer. 2025 Mar 19;25:505. doi: 10.1186/s12885-025-13890-x (PMC11921695; doi:10.1186/s12885-025-13890-x)

# Supplementary appendix 6 – Change in total tumour area during the course of treatment for the any-dose/safety population


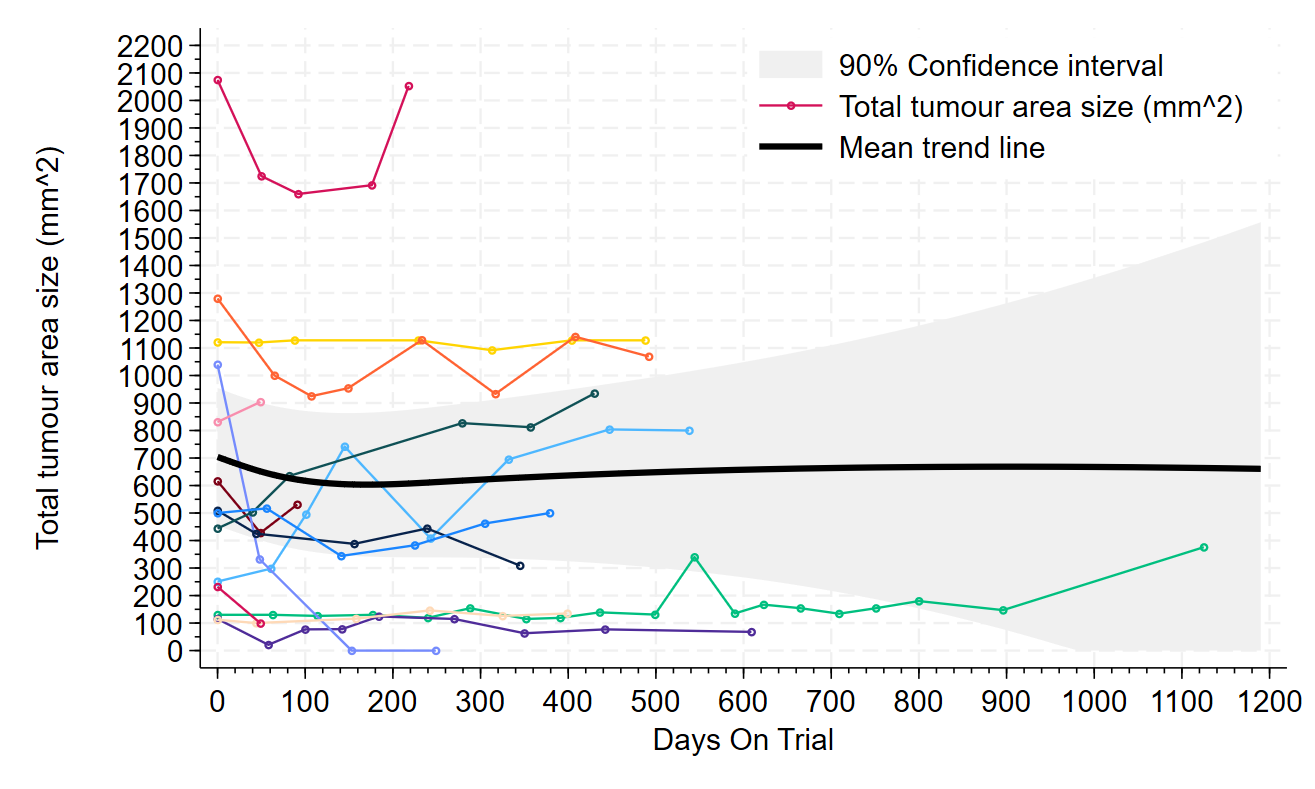

Supplement: Supplementary file 6 — Supplementary appendix 6. Change in total tumour area during the course of treatment for the any-dose/safety population [file 12885_2025_13890_MOESM6_ESM.docx]
