# Supplementary material for: Selumetinib in Combination with Anti Retroviral Therapy in HIV-associated Kaposi sarcoma (SCART): an open-label, multicentre, phase I/II trial
Source: BMC Cancer. 2025 Mar 19;25:505. doi: 10.1186/s12885-025-13890-x (PMC11921695; doi:10.1186/s12885-025-13890-x)
Supplement: Supplementary file 7 — Supplementary appendix 7. Number of patients experiencing adverse events and adverse reactions who received any dose of selumetinib [file 12885_2025_13890_MOESM7_ESM.docx]

# Supplementary appendix 7 – Number of patients experiencing adverse events and adverse reactions who received any dose of selumetinib

|  | Adverse Events | | | | | | | Adverse Reactions | | | | | | |
| --- | --- | --- | --- | --- | --- | --- | --- | --- | --- | --- | --- | --- | --- | --- |
| Category | Grade 1 | | Grade 2 | | Grade3 | | | Grade 1 | | Grade 2 | | Grade 3 | | |
|  | **N** | **E** | **N** | **E** | | **N** | **E** | **N** | **E** | **N** | **E** | | **N** | **E** |
| Blood and lymphatic system disorders | 4 | 5 | 1 | 1 | | 0 | 0 | 3 | 4 | 1 | 1 | | 0 | 0 |
| Cardiac disorders | 1 | 1 | 0 | 0 | | 0 | 0 | 0 | 0 | 0 | 0 | | 0 | 0 |
| Eye disorders | 6 | 9 | 3 | 3 | | 0 | 0 | 5 | 6 | 3 | 3 | | 0 | 0 |
| Gastrointestinal disorders | 12 | 47 | 7 | 13 | | 0 | 0 | 12 | 44 | 6 | 9 | | 0 | 0 |
| General disorders and administration site conditions | 11 | 33 | 5 | 5 | | 1 | 1 | 10 | 23 | 2 | 2 | | 0 | 0 |
| Infections and infestations | 2 | 2 | 4 | 6 | | 0 | 0 | 1 | 1 | 3 | 4 | | 0 | 0 |
| Injury, poisoning and procedural complications | 2 | 2 | 1 | 3 | | 0 | 0 | 0 | 0 | 0 | 0 | | 0 | 0 |
| Investigations | 15 | 133 | 4 | 7 | | 2 | 2 | 14 | 81 | 3 | 6 | | 0 | 0 |
| Metabolism and nutrition disorders | 5 | 13 | 0 | 0 | | 0 | 0 | 5 | 9 | 0 | 0 | | 0 | 0 |
| Musculoskeletal and connective tissue disorders | 4 | 4 | 0 | 0 | | 0 | 0 | 2 | 2 | 0 | 0 | | 0 | 0 |
| Nervous system disorders | 4 | 7 | 0 | 0 | | 0 | 0 | 4 | 5 | 0 | 0 | | 0 | 0 |
| Psychiatric disorders | 3 | 3 | 0 | 0 | | 0 | 0 | 3 | 3 | 0 | 0 | | 0 | 0 |
| Renal and urinary disorders | 2 | 2 | 0 | 0 | | 0 | 0 | 1 | 1 | 0 | 0 | | 0 | 0 |
| Respiratory, thoracic and mediastinal disorders | 4 | 11 | 1 | 2 | | 0 | 0 | 4 | 5 | 1 | 1 | | 0 | 0 |
| Skin and subcutaneous tissue disorders | 10 | 29 | 9 | 17 | | 3 | 3 | 10 | 24 | 9 | 16 | | 3 | 3 |
| Vascular disorders | 2 | 2 | 0 | 0 | | 0 | 0 | 0 | 0 | 0 | 0 | | 0 | 0 |

N: The number of individual patients that have experienced at least one adverse event at that particular grade; a patient who experiences the same adverse reaction at more than one grade will be included in each specific grade count.

E: The number of events that have been reported at that particular grade
