# Supplementary material for: Selumetinib in Combination with Anti Retroviral Therapy in HIV-associated Kaposi sarcoma (SCART): an open-label, multicentre, phase I/II trial
Source: BMC Cancer. 2025 Mar 19;25:505. doi: 10.1186/s12885-025-13890-x (PMC11921695; doi:10.1186/s12885-025-13890-x)
Supplement: Supplementary file 8 — Supplementary appendix 8. Number of grade < 2 adverse events reported [file 12885_2025_13890_MOESM8_ESM.docx]

# Supplementary appendix 8 – Grade <2 adverse events reported (where incidence at least 10 events)

| **All recorded adverse events**  **(CTCAE Grade ≤2)** | **Number of AEs reported** | **Number of patients experiencing at least one grade <2AE** |
| --- | --- | --- |
| Red Blood Cell Count Decreased | 22 | 7 (44%) |
| CPK Increased | 14 | 9 (56%) |
| ALT Increased | 13 | 5 (31%) |
| Albumin Decreased | 12 | 3 (19%) |
| AST Increased | 11 | 7 (44%) |
| Nausea | 11 | 7 (44%) |
| Vomiting | 11 | 3 (19%) |
| Fatigue | 10 | 9 (56%) |
